# Acetyl-NPKY of integrin-β1 binds KINDLIN2 to control endothelial cell proliferation and junctional integrity

**DOI:** 10.1016/j.isci.2024.110129

**Published:** 2024-05-28

**Authors:** Adama Sidibé, Vasyl V. Mykuliak, Pingfeng Zhang, Vesa P. Hytönen, Jinhua Wu, Bernhard Wehrle-Haller

**Affiliations:** 1 Department of Cell Physiology and Metabolism, University of Geneva, Centre Médical Universitaire, Rue Michel-Servet 1, CH-1211 Geneva, Switzerland; 2 Faculty of Medicine and Health Technology, Tampere University, Arvo Ylpön katu 34, FI-33520 Tampere, Finland; 3 Cancer Signaling and Microenvironment Program, Fox Chase Cancer Center, 333 Cottman Avenue, Philadelphia, PA 19111, USA; 4 Fimlab Laboratories, Biokatu 4, FI-33520 Tampere, Finland

**Keywords:** Cell biology, Cell, Structural biology

## Abstract

Integrin-dependent crosstalk between cell-matrix adhesions and cell-cell junctions is critical for controlling endothelial permeability and proliferation in cancer and inflammatory diseases but remains poorly understood. Here, we investigated how acetylation of the distal NPKY-motif of Integrin-β1 influences endothelial cell physiology and barrier function. Expression of an acetylation-mimetic β1-K794Q-GFP mutant led to the accumulation of immature cell-matrix adhesions accompanied by a transcriptomic reprograming of endothelial cells, involving genes associated with cell adhesion, proliferation, polarity, and barrier function. β1-K794Q-GFP induced constitutive MAPK signaling, junctional impairment, proliferation, and reduced contact inhibition at confluence. Structural analysis of Integrin-β1 interaction with KINDLIN2, biochemical pulldown assay, and binding energy determination by using molecular dynamics simulation showed that acetylation of K794 and the K794Q-mutant increased KINDLIN2 binding affinity to the Integrin-β1. Thus, enhanced recruitment of KINDLIN2 to Lysine-acetylated Integrin-β1 and resulting modulation of barrier function, offers new therapeutic possibilities for controlling vascular permeability and disease conditions.

## Highlights

• Crystal structures of acetylated and K794Q-mutated Integrin-β1 with KINDLIN2
• β1-K794Q induces KINDLIN2 binding and immature adhesions in endothelial cells
• β1-K794Q induces a transcriptomic change of junctional and proliferation genes
• β1-K794Q impairs endothelial barrier function and contact inhibition of proliferation

## Graphical abstract

**Figure:**
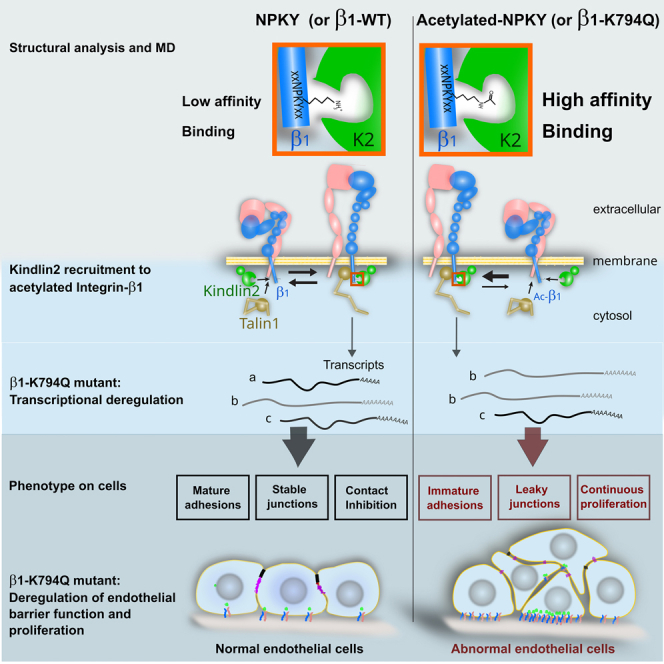

## Introduction

Changes in endothelial cell (EC) metabolism promote tumor angiogenesis by inducing excessive proliferation and permeability, leading to deterioration of drug delivery and resistance to chemotherapy.^1^^,^^2^ Although specific strategies are missing, enhancing the endothelial barrier function was proposed as an approach to improve vessel normalization in cancer.^3^ Endothelial barrier function is maintained by junctional complexes formed by the vascular endothelial (VE)-cadherin and tight junctional proteins including CLAUDIN5 as well as their cytoplasmic adapter proteins, linking the junctional structures to the actin cytoskeleton. However, improving endothelial barrier function by changing the composition and function of junctional proteins is challenging.^4^ Interestingly, EC adhesions to the extracellular matrix (ECM) mediated by the integrin-actin linkage were shown to be critical for both the establishment and disruption of cell junctions.^5^^,^^6^^,^^7^^,^^8^^,^^9^^,^^10^ It is thus primordial to understand the crosstalk between cell-ECM adhesions and cell-cell junctions, and to define the underlaying mechanisms with the aim to improve junctional control as a therapeutic strategy for vessel normalization.

Integrins are heterodimeric transmembrane receptors composed of alpha (α) and beta (β) subunits that link extracellular ligands to the actin cytoskeleton through intracellular adaptors.^11^ The Integrin-β1 subunit is highly expressed in EC and forms heterodimers with several α subunits, with critical roles in various physiological processes.^12^ Homozygous deletion of Integrin-β1 in ECs affects angiogenesis and causes embryonic lethality at E9.5 in mice.^13^ Integrin-β1 is required for EC polarity and lumen formation, as well as the correct localization of VE-cadherin to induce stability of endothelial junctions by p120-catenin.^5^^,^^6^ Several studies used genetically engineered mouse- or tissue-derived cells to reveal that Integrin-β1 controls EC survival and migration in angiogenesis.^7^^,^^8^ Although Integrin-β1-dependent signaling^7^^,^^14^^,^^15^^,^^16^^,^^17^ and transcriptomic programming^5^ were shown to be required for EC division, the mechanisms of EC proliferation remained unclear. In other experiments, Integrin-β1 was proposed to destabilize EC junctions,^6^^,^^9^^,^^10^ specifically implicating intracellular integrin adapters such as TALIN1,^9^ VINCULIN,^18^^,^^19^ and KINDLIN2.^20^^,^^21^ In addition, the intracellular Integrin-β1 adapter TENSIN1 was associated with lipopolysaccharide (LPS)-induced endothelial permeability.^10^ In contrast, TALIN1-dependent activation of Integrin-β1 fostered the integrity of EC junctions.^9^ These studies propose Integrin-β1-dependent signaling to be important for enhancing or reducing endothelial barrier functions.

Post-translational modifications, such as phosphorylation of Integrin-β1 and their adapters have been found to regulate adhesion initiation and stability.^22^^,^^23^ Furthermore, Integrin-β1 was found acetylated on Lysine 794 in a large proteomic study, however, without proposing a specific function.^24^ Particularly, the role of lysine acetylation for Integrin-β1 adapter recruitment needs to be defined. We recently found increased colocalization of KINDLIN2 with the acetylation mimetic K794Q mutant of Integrin-β1 (β1-K794Q-GFP) in focal adhesions, proposing an adhesion modulating role of Integrin-β1 acetylation.^25^ However, the influence of Integrin-β1 acetylation on EC function in general and cell-cell junctions in particular were not explored. Here we investigated the impact of the acetylation mimetic form of Integrin-β1 on ECs and their barrier functions. We found that acetylation of Integrin-β1 enhances the affinity for KINDLIN2 modulating cell adhesions, junctions and contact inhibition through transcriptional reprograming of ECs.

## Results

### Constitutive acetylation of Integrin-β1 changes the transcriptome and the structure of adhesions in endothelial cells

**Figure 1:**
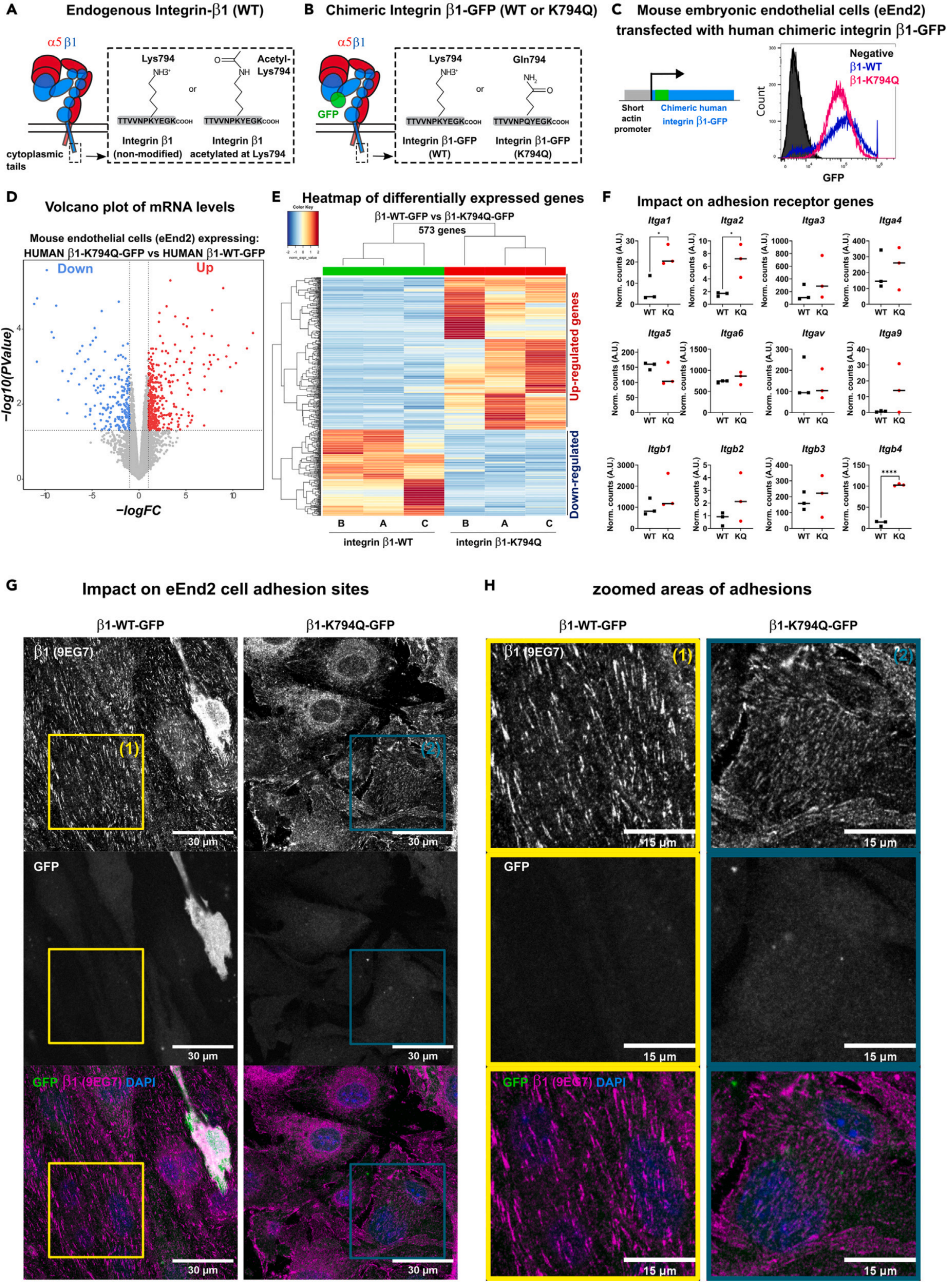
Integrin β1 acetylation-mimetic mutation alters gene expression and adhesions of mouse embryonic endothelial cells (A) Scheme of heterodimeric Integrin α5β1 (α5 in red and β1 in blue) in the inactive (bent) state. The cytoplasmic tail susceptible to Lys794 acetylation is shown in the dashed rectangle. (B) Integrin heterodimer with a chimeric β1-GFP (β1-WT: wildtype or β1-K794Q: acetylation mimetic K794Q mutant). (C) Construct for stable expression of chimeric human integrin β1-GFP (β1-WT or β1-K794Q) in mouse embryonic endothelial eEnd2 cells (left panel) and confirmed by flow cytometry (left panel). (D) Volcano plot of differentially expressed mRNA in mouse endothelial cells transfected with β1-WT or β1-K794Q constructs. Transcripts showing significant changes by RNA sequencing (RNA-seq) are shown in blue for downregulated or red for upregulated genes respectively (threshold for fold change FC ≥ 2 and *p*-value ≤0.05). (E) Hierarchical heatmap of differentially expressed genes between β1-WT and β1-K794Q cells, representing the Euclidean distance of clusters of genes in a row and sample replicates in column. Between integrin β1-WT-versus K794Q-expressing eEnd2 cells, 366 transcripts were significantly increased and 207 decreased. (F) Among adhesion receptor encoding transcripts Integrin α1 (*Itga1*), α2 (*Itga2*), and β4 (*Itgb4*) showed significant increases in response to the expression of acetylation mimetic Integrin β1-K794Q. Lines indicate medians of three replicates (dots) and the stars indicate significance (stars; *p*-value ≤0.05 in unpaired t-test). The RNA-seq was repeated once providing similar results (supporting data Figures S1 and S2, Tables S1 and S2). (G) Confocal microscopy of localization and distribution of activated Integrin β1 (9EG7 staining) and nuclei (DAPI) in eEnd2 cells expressing WT or acetylation mimetic K794Q human Integrin β1-GFP. Areas delineated by yellow (1) and green (2) squares are shown at higher magnification in (H). Pictures are representative of three independent experiments. Scalebar = 30 μm or 15 μm as indicated.

The Integrin-β1 subunit has been found acetylated in its cytoplasmic tail^24^ at Lys794 of the distal NPKY-motif as depicted in Figure 1A. This modification was recently associated with a modulation of Integrin-β1 function and enhanced fibronectin deposition in fibroblasts.^25^ However, its relevance in EC, or effects on gene transcription remain unknown. To analyze modulation of gene expression and morphological changes, we mimicked a constitutively acetylated Integrin-β1 by expressing the K794Q-mutant. To track and quantify Integrin-β1 expression, we used an extracellularly tagged human Integrin-β1-GFP chimera and expressed wildtype (β1-WT-GFP) or mutant forms (β1-K794Q-GFP) (Figure 1B), both induced by a short human β-actin promoter, allowing constitutive, but moderate expression (Figure 1C). We mutated the Lys794 to glutamine (Gln), an amino acid that was previously used to mimic acetylated Lys-residues of proteins^25^ as it provides charge neutralization and shares hydrogen-bonding capacities with the acetyl group. We stably transfected and subsequently FACS-sorted (Figure 1C) mouse embryonic endothelial eEnd2 cells expressing either version of the β1-GFP chimera. The expression levels of the two versions of β1-Integrin were similar and did not change along the passages of the cells, indicating no effect of the K794 acetylation on the protein surface expression or stability (not shown). We further analyzed the influence on the transcriptome by sequencing the total mRNA (RNA-seq). Two RNA-seq experiments were performed on the same samples providing similar results. We found more than 570 differentially expressed genes in eEnd2 cells expressing β1-WT-GFP versus β1-K794Q-GFP (Figures 1D and S1), exhibiting 366 down-regulated and 207 up-regulated genes. The analysis of ECM receptors showed a K794Q-induced increase in expression of genes encoding for Integrin subunits such as *Itga1*, *Itga2* and *Itgb4* (Figures 1F, S1, and S2, see Tables S1 and S2 respectively for Gene Ontology and the Kyoto Encyclopedia of Genes and Genomes). However, levels of mouse *Itga5*, *Itgav*, *Itgb1*, or *Itgb3* among others were not impacted by expressing K794Q mutant of β1-Integrin in eEnd2 cells. The analysis of the activated form of β1-Integrin at adhesions by using the mAb clone 9EG7 by confocal microscopy showed that the expression of the K794Q mutant of β1-Integrin in EC leads to the formation of dense and thin fibrillar adhesions covering large surfaces of the basal cell membrane (Figures 1G and 1H). The adhesions formed in β1-K794Q cells appeared chaotic and immature, failing to form peripheral focal adhesions. These results demonstrated that a constitutive acetylation of β1-Integrin, modeled here by Lys-to-Gln mutation, induces profound changes in integrin-adhesions and affected the EC transcriptome.

### Integrin-β1 acetylation enhances endothelial cell proliferation and affects contact inhibition at high cell densities

**Figure 2:**
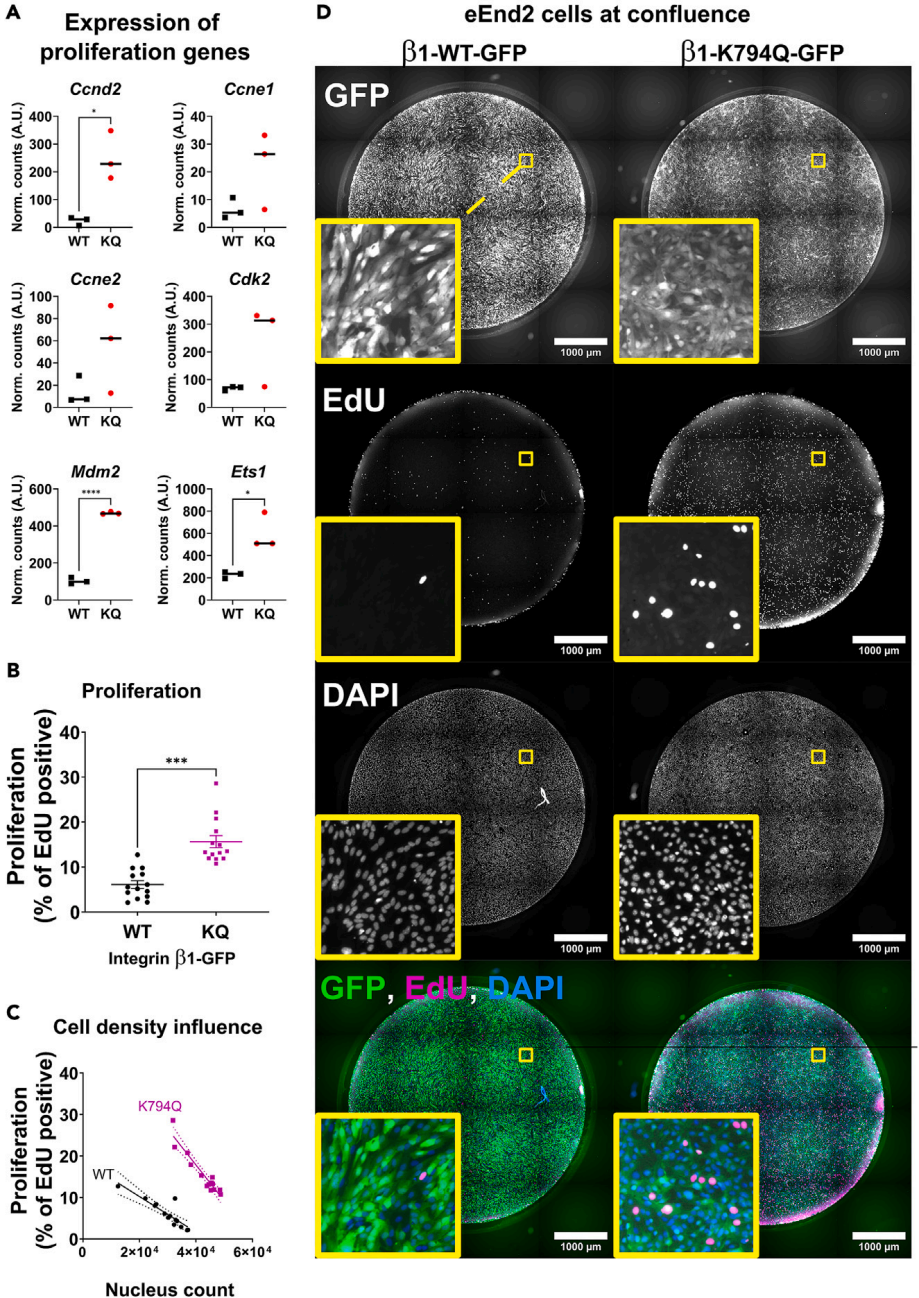
Expression of acetylation mimetic Integrin-β1 induces uncontrolled proliferation of endothelial cells (A) Mouse eEnd2 cells constitutively expressing β1-K794Q induced cell proliferation-promoting genes. Lines indicate medians of three replicates (dots) and the stars indicate significance (stars; *p*-value ≤0.05 in unpaired t-test). (B) Quantification of cell proliferation by nuclear EdU incorporation in confluent mouse eEnd2 cells, expressing WT or K794Q-mutant Integrin-β1. Each dot represents the percentage of proliferating cells in one well and their means are indicated by bars. Stars indicate significance when *p*-value ≤0.05 by unpaired t-test (14 wells per condition). Supporting data are included in Figure S3. Similar cell proliferation results were found in more than 4 independent experiments. (C) Analysis of cell proliferation in respect to cell densities. Cell proliferation rates were plotted as functions of nuclear densities. Please note that proliferation of eEnd2 cells expressing β1-WT Integrin was inhibited at high cell densities, whereas this inhibition of proliferation was abrogated in eEnd2 cells expressing the acetylation mimetic K794Q mutant. Each dot represents the percentage of proliferating cells in one well (14 wells per conditions). Linear regression lines show the proliferation-rate along different cell densities. (D) Fluorescent images of GFP, EdU and DAPI, of eEnd2 cell cultures expressing WT versus K794Q mutant Integrin-β1-GFP chimera. Stitched images allow to appreciate the entire well. Please note the absence of EdU incorporation in β1-WT, versus continuing EdU staining of nuclei in β1-K794Q expressing cells. Single channels in grayscale and the merged image are presented. GFP (green) shows the transfected human Integrin β1-GFP chimera, EdU (magenta) indicates proliferating cells and DAPI (blue) shows staining of all the nuclei. Scalebar = 1000 μm. Zoomed-in areas represent the yellow squares. Similar results were found in more than three independent experiments. See Figure S3 for supporting results.

Given the important role of integrins and matrix adhesions in cell proliferation, we analyzed the impact of a constitutive acetylation of Integrin-β1 on the expression of genes controlling cell proliferation. As shown in Figure 2A, several of those were upregulated in cells expressing β1-K794Q-GFP compared to those expressing β1-WT-GFP. These transcripts included cyclins such as *Ccnd2* and *Ccne1,* as well as cyclin-dependent kinase (*Cdk)2*, (Figures 2A, S1, and S2). The transcript encoding for Mdm2, the regulator of the tumor suppressor p53, was also upregulated by β1-K794Q-GFP. In addition, the expression of mitogenic factors such as placental growth factor (*Pgf*) was increased by β1-K794Q-GFP, suggesting an enhancement of cell proliferation by Integrin-β1 acetylation. We studied cell proliferation of eEnd2 cells using DNA incorporation of 5-ethynyl-2′-deoxyuridine (EdU) to detect dividing cells and recorded the percentage of EdU positive cells as an index of cell proliferation.^26^^,^^27^ As illustrated in Figures 2B and S3, eEnd2 cells expressing β1-K794Q-GFP showed more EdU positive nuclei and a higher proliferation compared to cells expressing the wildtype versions of the Integrin-GFP chimera. Since cell confluence is able to block EC proliferation under normal angiostatic condition,^28^^,^^29^^,^^30^ we tested the impact of different cell densities on eEnd2 cell proliferation by plotting the proliferation index as a function of the total cell count (Figure 2C). EdU incorporation in cells expressing β1-WT-GFP was inhibited by high cell density, whereas β1-K794Q-GFP-expressing cells failed to become quiescent Figure 2D). This proposes that constitutive acetylation of Integrin-β1 in EC enhances cell proliferation and an obliteration of the density-induced cell proliferation arrest at confluence.

### Acetylation of Integrin-β1 alters endothelial cell junctions

**Figure 3:**
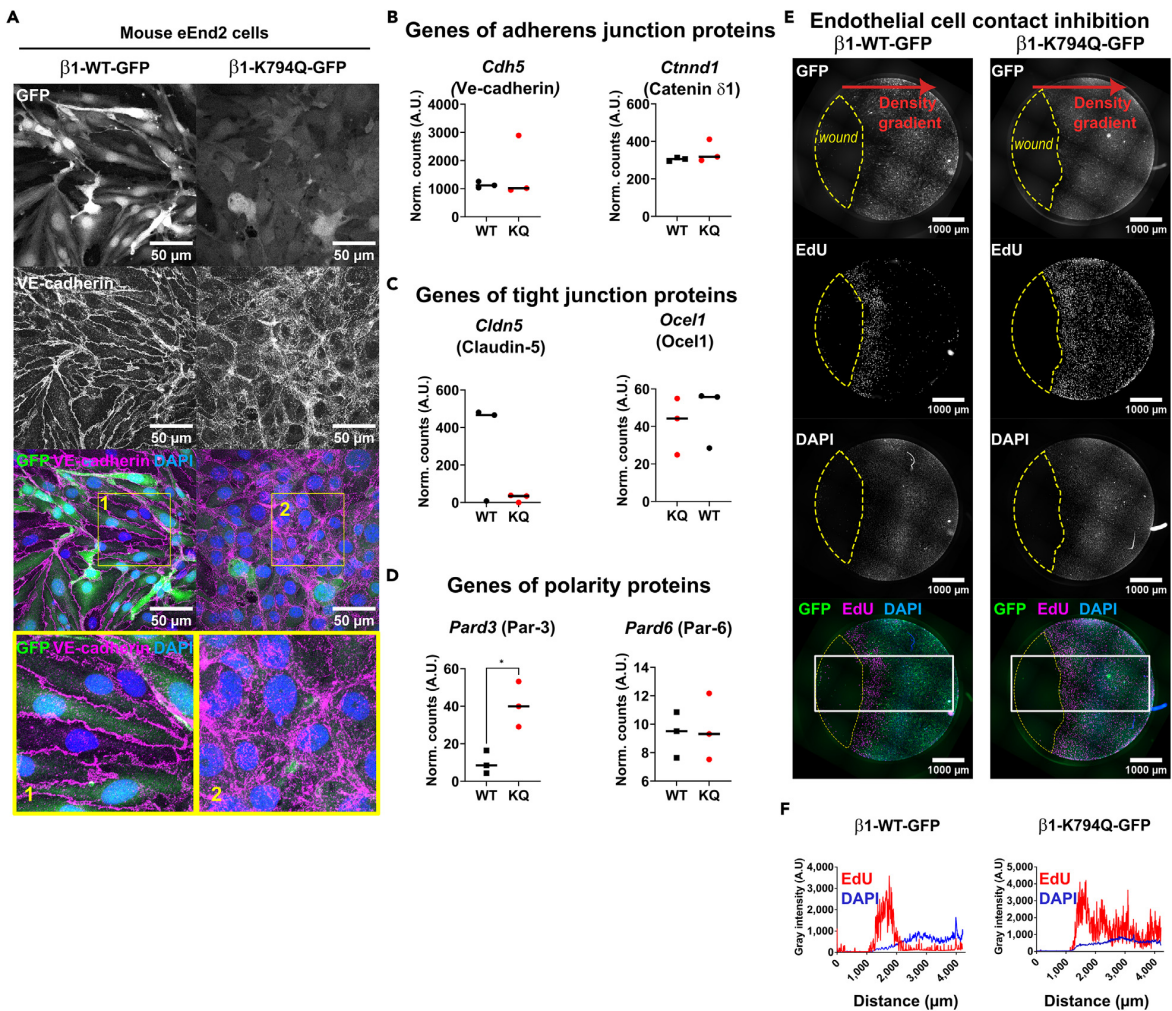
Induction of junctional alterations and perturbation of contact inhibition by expressing acetylation mimetic β1-K794Q Integrin (A) Illustration of endothelial cell junctions in mouse eEnd2 cells expressing either β1-WT or the acetylation mimetic β1-K794Q mutant. Confluent eEnd2 cells expressing either WT or K794Q-mutant Integrin-β1-GFP (green channel) were stained with an anti-VE-cadherin (VE-cad, magenta) antibody. Single channels are in grayscale and the merge in color. Scalebar = 50 μm. Nuclei are stained with DAPI (blue). Zoomed areas of the monolayers show differences in cell-cell contact formation between β1-WT and β1-K794Q expressing eEnd2 cells. The data are representative of >4 independent experiments. (B–D) Examples of specific gene expression patterns derived from the microarray dataset (RNAseq I) were presented to illustrate genes involved in the formation of adherens junction (B), tight junction (C) and cell polarity (D). (E) Cell confluence-induced contact inhibition and wound healing assay in eEnd2 cells expressing either β1-WT or β1-K794Q versions of human Integrin-β1. Stitched pictures allow to appreciate the proliferation state in the monolayer and near the zone of proliferation/migration at the wound edge. Scalebar = 1000 μm. The red arrow points toward the zone with the highest cell density. Dashed lines delineate wound areas. Please note that eEnd2 cells expressing β1-WT proliferated (EdU staining) only at the border of the wound, where cell density was the lowest, compared to the inhibition of proliferation in areas of high cell density. In contrast, eEnd2 cells expressing the K794Q mutant of Integrin-β1-GFP proliferated at the wound edge but also in areas of high cell density. (F) Fluorescence intensity profiles of EdU and DAPI in the white rectangles drawn on the merged pictures in (E). The profiles of DAPI intensity show the gradient of cell confluence with the lowest density bordering the wound and the highest far from the wound in both cells expressing β1-WT-GFP or β1-K794Q-GFP. See also Figure S4 for data supporting the finding based on two independent experiments with >10 replicates per condition.

Cell junctions are crucial for controlling cell proliferation at confluence.^28^ We analyzed the quality of cell-cell junctions established between eEnd2 cells expressing either β1-WT-GFP or the K794Q mutant. Staining VE-cadherin showed well-organized and straight junctions between EC expressing β1-WT-GFP (Figure 3A), a feature that marks the tightness of intercellular junctions. β1-WT-GFP expressing eEnd2 cells showed non-overlapping flattened cells in a well-defined monolayer, which was in contrast to β1-K794Q-GFP expressing cells, showing more diffusive and chaotic cell junctions. VE-cadherin localization showed anarchic superimpositions of peripheral regions of adjacent cells, especially in regions of high cell density (not shown). Due to these junctional alterations, we analyzed the effect of Integrin-β1 acetylation on the expression of genes encoding for junctional molecules in eEnd2 cells. While the expression of VE-cadherin (*Cdh5*) and its cytoplasmic adapters such as p120-catenin/catenin-δ1 (*Ctnnd1*) were not changed, the transcripts of Claudin5 (*Cldn5*) were decreased in β1-K794Q-GFP mutant compared to β1-WT-GFP expressing cells (Figures 3B and 3C). However, no difference was observed in the mRNA levels of the tight junction protein Occludin/ELL domain containing 1 (*Ocel1*). The altered monolayer organization indicated a cell polarity issue, potentially similar to previous observations in mouse Integrin-β1 deficient embryo.^5^ Accordingly, the expression of the polarity gene *Pard3* encoding for Partitioning-defective protein (Par)-3 and *Jam3* for junctional adhesion molecule (JAM)-C were both increased in β1-K794Q-GFP cells (Figure 3D) suggesting an attempt to balance the loss of endothelial polarity. Altogether, these results demonstrate that constitutive acetylation of Integrin-β1 alters the organization of EC junctions and polarity, consistent with the appearance of areas of superimposed cells, proposing an explanation for the deficit in contact inhibition.

### Maintenance of wound-healing response despite lack of contact inhibition

The cell ability to dynamically regulate contact inhibition was then specifically studied by analyzing β1-K794Q-GFP induced proliferation in confluent areas compared to sub-confluent ones using a wound healing assay (Figure S4A). During wound healing, the cell migrating front presents loosened junctions leading to lower cell density that induces directional cell migration and proliferation at the wound border. We developed an air-bubble-induced wound healing technique and analyzed the proliferation by recording the localization of EdU positive eEnd2 cells in respect to the wound edge (Figures 3E and S4B). With both versions of Integrin-β1, the wound edge was populated by proliferating cells. However, far away from the wound, in regions of high cell density, β1-WT-GFP cells showed a strong inhibition of proliferation, whereas cells expressing β1-K794Q-GFP presented high numbers of proliferating cells. We quantified EdU and DAPI fluorescence intensities across the wound edge and within the cell layer (Figures 3E and 3F). The eEnd2 cells expressing β1-WT-GFP presented main peaks of EdU at the wound border representing the cell migration front with lower density of DAPI labeled nuclei (lower overall DAPI intensity). Toward the region of high cell density only a few EdU intensity peaks were visible throughout the monolayer. In contrast, β1-K794Q-GFP cells presented high EdU intensity at the wound edge, as well as in the confluent monolayer, exhibiting a high DAPI intensity. These results demonstrate that β1-WT-GFP cells underwent efficient contact inhibition of cell proliferation at high cell densities and induced proliferation at the migration front during wound healing. Similarly, cells expressing acetylated Integrin-β1 were able to induce proliferation at the migrating wound edge by responding to environmental cues but failed to form mature cell junctions that would lead to contact inhibition of growth.

### Constitutive acetylation of Integrin-β1 imposes an integrin-dependent signaling state

How Integrin-β1 acetylation induced cellular signaling capable of modifying EC adhesions, junctions and proliferation remains unclear. We first hypothesized that this process would involve the induction of the Hippo-Yap signaling, a mechano-sensing pathway that can be modulated by cell-ECM interaction through Integrin-β1 function. Accordingly, we analyzed the expression of genes that were reportedly^31^^,^^32^ targeted by the transcription factor Yes-associated protein (Yap) within our transcriptomic data. As shown in Figure S5, expression of β1-K794Q-GFP had no impact on most Yap target genes as compared to β1-WT-GFP. This indicated that Yap transcription factor function was not stimulated by acetylation of Integrin-β1 in EC.

**Figure 4:**
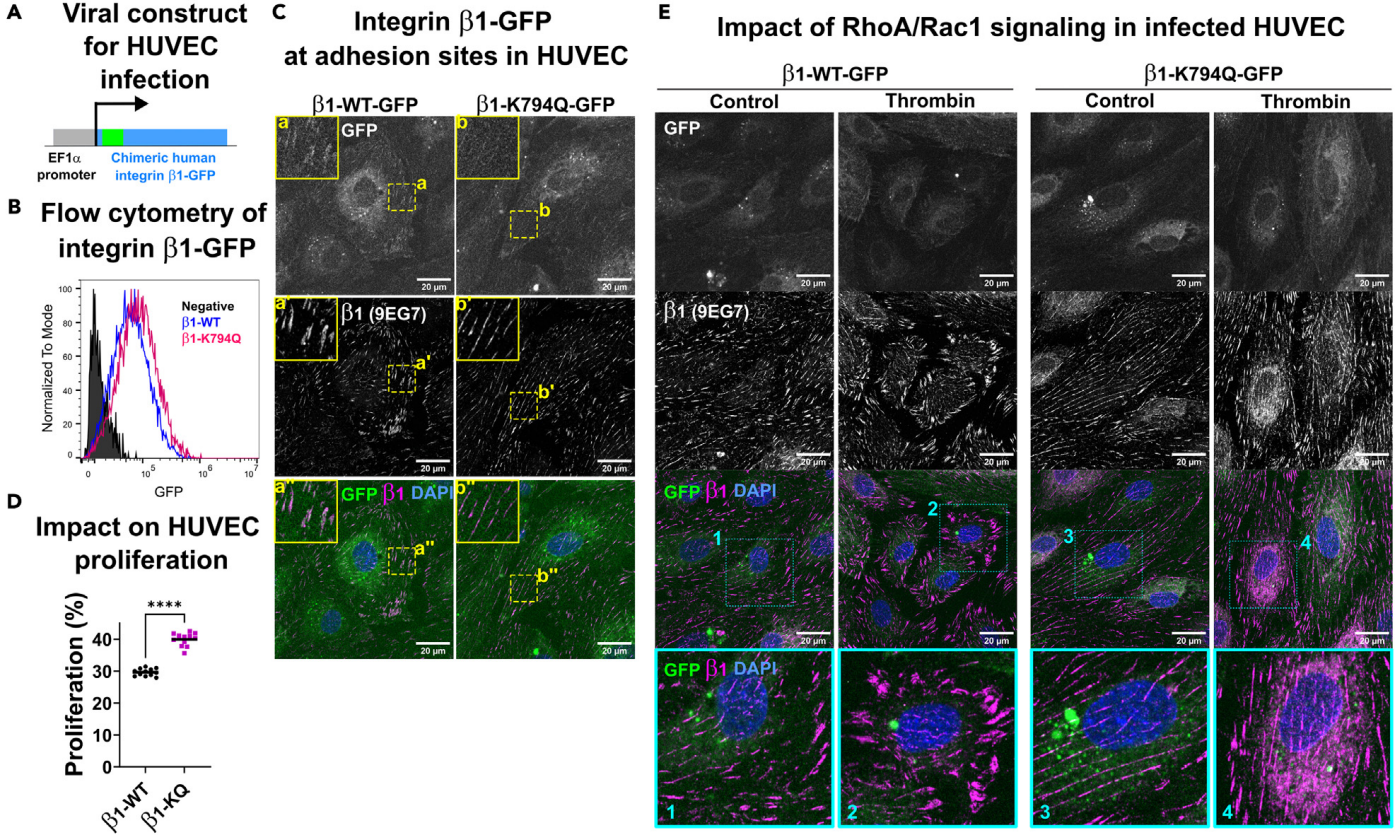
Acetylation mimetic Integrin-β1 induces MAPK signaling and resistance to RhoA-mediated adhesion remodeling in HUVEC (A) HUVEC were transfected with a lentiviral construct expressing the chimeric human Integrin β1-GFP (WT or K794Q) under the control of the EF1a promoter, and expression confirmed by flow cytometry (B). (C) Confocal microscope images of β1-GFP transfected HUVEC showing transfected Integrins in adhesion sites by GFP and staining for activated integrins by 9EG7 antibody (magenta). Greyscale of single channels and the merge in color are shown. Zoomed views of the area within the dashed yellow squares are shown in the upper right corners. Scalebar = 30 μm. (D) Relative proliferation indices (%) of HUVEC expressing β1-WT or β1-K794Q mutant Integrin-β1. Each dot represents the proliferation index of a single well and the mean is indicated by a line. The significance was shown as stars when *p*-value ≤0.05 in unpaired t-test (12 wells per conditions). Similar results were found in two independent experiments. (E) Thrombin treatment induced adhesion remodeling in HUVEC. Confluent HUVEC expressing β1-WT or β1-K794Q were treated with or without (control) thrombin and Integrin β1-containing cell adhesions were identified by GFP imaging (green) and staining with mAb 9EG7 (magenta). Single channels are shown in greyscale and the merge in color. Scalebar = 20 μm. Magnified views are shown of the areas delineated by dashed rectangles (1–4). The illustration is representative of two independent experiments. See Figure S7 for supporting western blot data.

A second mechanism that links Integrin-β1 acetylation to cell junctions could be mediated by Ras homolog (Rho) GTPases-dependent actin contractility and adhesion remodeling.^33^^,^^34^^,^^35^ To test this hypothesis, we developed a lentiviral expression system for the expression of β1-GFP (WT or K794Q) under the control of the elongation factor alpha (EF1α) promoter, allowing a constitutive expression in human umbilical vein endothelial cells (HUVEC) as outlined in Figure 4A. Efficient expression of the two versions of Integrin-β1-GFP chimeras was confirmed by flow cytometry (Figure 4B) and the analysis of cell adhesions by confocal microscopy showed localization of chimeric β1-GFP in cell-matrix adhesions exhibiting the active conformation of Integrin-β1, assessed by 9EG7 mAb staining, for both β1-WT- and β1-K794Q-GFP (Figure 4C). Consistent with the phenotypes observed in eEnd2 cells, the permanent acetylation of Integrin-β1 in HUVEC also induced the formation of dense and thin fibrillar-like adhesions that covered the basal membrane of the cells. In addition, the expression of β1-K794Q-GFP also increased HUVEC proliferation, phenocopying eEnd2 cells in several aspects (Figure 4D). To analyze the remodeling capacity of the different Integrin receptors, we induced Rho-dependent contractility by thrombin treatment,^36^ in HUVEC expressing β1-K794Q-GFP or β1-WT-GFP (Figure 4E). Thrombin-treatment led to efficient retraction of HUVEC expressing both versions of Integrin-β1-GFP. Notably, the treatment with thrombin induced a remodeling of central, thin fibrillar adhesions into thick peripheral focal adhesions in HUVEC expressing β1-WT-GFP. In contrast, the dense and thin fibrillar-like adhesions of β1-K794Q-GFP expressing HUVEC were more resistant to thrombin-induced remodeling and remained unchanged, despite cell retraction and junctional disruption. Similar results were found for Integrin-expressing eEnd2 cells. These data suggest that adhesions formed in HUVEC expressing the acetylation mimetic mutant of Integrin-β1 were maintained in an immature state.

Since αVβ3 Integrin, which is primarily localized in focal adhesions, has previously been associated with intracellular signaling, we asked whether the β1-K794Q-GFP mediated induction of cell proliferation was linked to an indirect signaling via αvβ3 Integrin. To compare Integrin-β1 dependent signaling with αvβ3 mediated signaling, we plated eEnd2 cells stably transfected with the different integrin β1 forms on substrates coated with different ECM ligands showing binding specificity for either β1 or αvβ3 Integrins and analyzed cell proliferation by EdU incorporation. As shown in Figure S6, the proliferation advantage in eEnd2 cells conferred by β1-K794Q-GFP expression, was present on all Integrin ligands, but with the highest indices for Laminin and Fibronectin, the preferred ligands of α3β1^37^ and α5β1,^38^ respectively. Proliferation was also high, when cells were plated on collagen/gelatin, the ligand for α1β1 and α2β1.^39^ Notably, the proliferation rate of eEnd2 cells was the lowest when plated on vitronectin, a preferred ligand for αVβ3.^40^ These results indicate that the contribution of Integrin-β3 activity on the eEnd2 proliferation was negligible, suggesting that ligand-engaged Integrin-β1 and even more so, acetylated Integrin-β1 induced proliferation of EC.

Another signaling pathway that depends on Integrin-β1 function and is downstream of KINDLIN2 is the mitogen-activated protein kinases (MAPK) that was previously shown to be induced by Integrin-dependent cell-matrix adhesion.^17^ To test this pathway, we analyzed the activation state of MAPK in HUVEC expressing either β1-WT-GFP or β1-K794Q-GFP. For this, we monitored the phosphorylation levels of the extracellular signal-regulated kinases (ERK1 and ERK2) in HUVEC stimulated by the vascular endothelial growth factor (VEGF), as well as treated with the combination of Tyrphostin, a potent tyrosine kinase inhibitor, and the inhibitor of phosphoinositide 3′ Kinase (PI3K) LY294002 (T + L), to efficiently inhibit MAPK signaling. In HUVEC expressing the β1-WT-GFP, a low basal level of ERK phosphorylation in the presence of DMSO (D) was increased by VEGF treatment, while it was inhibited by the inhibitors (Figures S7A and S7G). However, in HUVEC expressing β1-K794Q-GFP, the basal level of ERK phosphorylation was higher, and was not further augmented by the treatment with VEGF, although efficiently blocked by inhibitors of MAPK signaling. Integrin-β1 function can also be mediated by focal adhesion kinase (FAK) signaling. However, we did not find an increase in the inactive form of SRC (p-Y530), an activator of FAK (Figures S7C and S7I). To assure similar amounts of extracted proteins on the blot, we stained the membranes with Ponceau red directly after blotting and performed anti-Tubulin staining subsequently (Figures S7E and S7M). The protein levels of VE-CADHERIN (Figures S7B and S7H) and VINCULIN (Figures S7D and S7J) showed trends of increase in HUVEC with permanent acetylation of Integrin-β1, but this trend was variable between experiments. Altogether, these results propose that Integrin-β1 acetylation enhances the activation of the MAPK signaling pathways, which causes proliferation and reduced junctional integrity. Since enhanced Integrin-dependent signaling could be critical during tissue regeneration or inflammation, a better understanding of the underlaying mechanisms warranted further structural analysis.

### Fitting of acetyl-K794 of Integrin-β1 to the KINDLIN2 binding pocket

**Figure 5:**
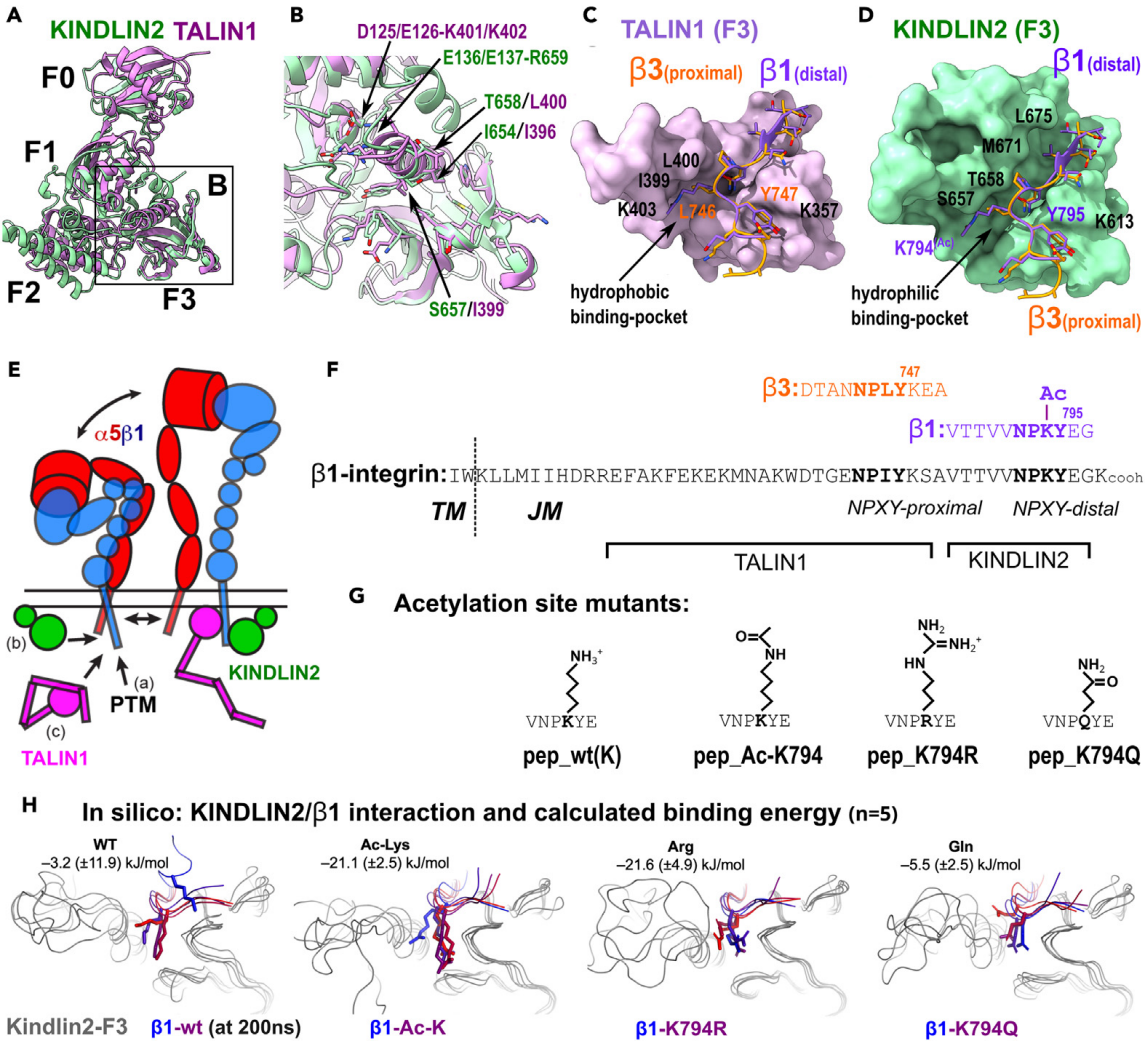
Modeling and in-silico study of the influence of Integrin-β1 acetylation on KINDLIN2 binding (A) Comparison of the structures of KINDLIN2 and TALIN1 showing the FERM folded head domain of TALIN1 superimposed on the KINDLIN2 protein, exhibiting a deleted PH-domain. (B) Zoom on the Integrin-binding pockets of TALIN1 and KINDLIN2, highlighting similarities and distinct amino acid residues involved in the adapter specific interactions with the proximal (TALIN1) and distal (KINDLIN2) NPXY-motifs. (C) Crystal structure of TALIN1 FERM-domain (PDB: 6vgu) with the proximal β3 (PDB: 6vgu), and modeled distal acetylated (Ac-K794) NPKY motif of β1. Relevant residues are indicated. (D) Crystal structure of KINDLIN2 (PDB: 5xq0) with the modeled proximal β3 and modeled distal acetylated NPKY motif of Integrin-β1. (E) Illustration of the hypothetical cascade of NPKY-acetylation (a, PTM: post-translational modifications), with subsequent binding of KINDLIN2 (b) and TALIN1 (c) to the distal and proximal portions, respectively of the activated Integrin-β1 in the α5β1 heterodimer. (F) Illustration of the primary sequence of the cytoplasmic tail of Integrin-β1 with the positions and sequences of the proximal and acetylated distal NPXY motifs represented in C and D. The transmembrane (*TM*) and Juxtamembrane (*JM*) positions are indicated. (G) The sequences of the peptides encompassing the distal NPKY-motif of Integrin β1 with or without Lys acetylation, or with Lys mutations of K794R or K794Q. (H) Final snapshots from 200 ns molecular dynamics (MD) simulations for Integrin-β1 complexed with KINDLIN2 showing position of the side chain of the residue 794 in Integrin β1, and contribution to the binding energy by non-modified (WT) K794, acetylated (Ac-Lys), K794R (Arg), and K794Q (Gln). Integrin-β1 is colored from blue to red to show the results of the five different simulations, where replica 1 is blue and replica 5 is red.

Post-translational modifications of the cytoplasmic tail of Integrins, such as phosphorylation were reported to regulate the binding of intracellular adapters.^22^ Cytoplasmic adapter proteins including KINDLIN2, Integrin-β1 binding protein 1 (ICAP1) and to a lesser extend FilaminA were shown to bind the Integrin-β1 peptide in the proximity of the K794-containing distal NPXY-motif.^41^^,^^42^^,^^43^^,^^44^^,^^45^ Thus, acetylation of K794, by neutralizing the positive charge, and enlarging the lysine side chain could modulate the recruitment of these adapters. In contrast to classic PTB-domain containing adapter proteins, such as TENSIN1 or ICAP1; TALIN1 as well as KINDLIN2 show an open but rigid binding pocket for the NPXY-motifs.^46^ The open binding pocket is maintained due to electrostatic interdomain FERM contacts at the C-terminal end of the F3 (PTB-domain) helix with conserved acidic residues in the F1-subdomain in both the KINDLIN2 and TALIN1 FERM domains (Figures 5A and 5B). This inter-domain interaction is absent in classical PTB domains allowing a more flexible positioning of the F3-helix. When considering the binding of Integrin-β1 tails to the rigid binding pockets of TALIN1 and KINDLIN2, there are specific differences between these adapter proteins in respect to the amino acids contacting the X-position of the NPXY-motif. While TALIN1 binds the membrane proximal NPL/IY-motif with hydrophobic leucine and isoleucine residues as illustrated with the β3-ligand (Figure 5C), KINDLIN2 faces the distal NPXY-motif, carrying the positively charged lysine at the X-position, with smaller, rather hydrophilic residues such as serine and threonine (Figure 5D).

While many residues are similar between the TALIN1 and KINDLIN2 Integrin-binding pockets, there are also important differences. For example, KINDLINs have an additional C-terminal α-helix that creates a larger hydrophobic cavity around the proline residue in the bound NPXY-peptide.^47^ Indeed, some Integrin-ligands have the proline replaced with a large hydrophobic side chain (i.e., Ile in KINDLIN binding motif in β3-Integrin; NITY). Another important structural difference concerns the above-mentioned residues S657 and T658 in KINDLINs (numbered according to KINDLIN2), which correspond to hydrophobic residues I399 and L400 in TALIN1 (Figures 5B–5D). These structural deviations between TALIN and KINDLIN create different binding pockets for the X-residue side chain in the NPXY-motif, being hydrophobic in TALIN1 (to accommodate Ile and Leu), and wider and hydrophilic for KINDLINs (multiple residues found in integrins (K,T,R) (compare Figure 5C with D). Thus, due to the difference in the size of the pockets and Integrin-ligand fitting, TALIN1 might bind preferentially the proximal NPLY-motif (β3-ligand in Figure 5C) while KINDLIN2 binds the distal NPKY-motif, as observed in the published structure (PDB: 5xq0).^42^

Based on the inspection of the Integrin-adapter complexes, an even better accommodation of the K794 side-chain on KINDLIN may be obtained, when the Integrin-peptide exhibits an acetylated lysine residue (Ac-K794) (Figure 5D). We decided to address this computationally, biochemically as well as structurally.

### Influence of K794 acetylation on integrin-β1-KINDLIN2 binding energy

To obtain dynamic atomistic models of the complexes between KINDLIN2 and various modified Integrin peptides, we conducted molecular dynamics (MD) simulations for KINDLIN2 complexed with β1-Integrin cytoplasmic tails. We analyzed the binding energy of KINDLIN2 with WT β1-peptide (lysine K794) or modified peptides carrying acetylated lysine (Ac-Lys), arginine (Arg or K794R) or glutamine (Gln or K794Q). Previously published crystal structure 5xq0 obtained from a KINDLIN2-Integrin-β1 fusion construct^47^ was used as a starting conformation in the simulations, and five 200-ns independent replicas were generated. As shown in Figure 5H, in 1 out of 5 MD trajectories with the non-modified control peptide (WT), the K794 dissociated from its binding pocket within the 200-ns simulation time, proposing that the non-modified Integrin-peptide does not strongly interact with KINDLIN2. We also found that the lysine side chain does not significantly contribute to the overall binding energy of the Integrin peptide to KINDLIN2. In contrast, such dissociation events were not observed when KINDLIN2 was complexed with Integrin peptides carrying Ac-Lys794 or K794R mutations. In all 5 simulations, the peptides stayed attached and the acetyl-lysine or arginine side-chains lodged deeply into the peptide binding pocket of KINDLIN2, contributing strongly to the binding energy (dG = −21.1 ± 2.5 kJ/mol and −21.6 ± 4.9 kJ/mol, respectively) as compared to the non-modified WT peptide (dG = −3.2 ± 11.9 kJ/mol). A similar behavior was found for the glutamine side-chain, although the penetration depth of the glutamine side chain was reduced, leading to a lower overall contribution to the binding energy (dG = −5.5 ± 2.5 kJ/mol) as compared to Ac-Lys and K794R peptides. These results were consistent with the static models described above and suggested an enhanced interaction of KINDLIN2 with the acetylated K794, K794R and K794Q mutant peptides compared to the wildtype peptide.

### Acetylation of Integrin-β1 at K794 enhances KINDLIN2 binding

Full activation of Integrin-β1 requires the binding of TALINs at the proximal NPXY as well as KINDLINs at the distal NPXY-motif, as indicated by structural data and previous studies^41^ (Figure 5E). We explored biochemically whether acetylated K794 of β1-Integrin can bind to KINDLIN2 and determined the selectivity of KINDLIN2 binding pocket for the modified X-residue of the NPXY-motif. Therefore, biotinylated peptides encompassing the distal NPXY-motif of Integrin-β1 (Figure S8A), the binding site for KINDLINs were synthesized with different substitutions of the X-residue (K794 in β1) (Figure S8B). The peptides included the non-modified K794 (pep_wt), acetylated K794 (pep_Ac-K794), the acetylation mimetic mutation K794Q (pep_K794Q), the positively charged acetyl-lysine mimetic K794R mutation (pep_K794R) and the scrambled acetylated peptides (pep_Scr.). These peptides were used to pulldown adapter proteins from protein extracts of HUVEC and the amount of captured endogenous KINDLIN2 was analyzed by western blotting. As shown in one out of several similar pull-down experiment Figures S8C and S8D, acetylated peptide pulled down more KINDLIN2 than non-modified peptide or the scrambled peptide. The peptide carrying the acetylation mimetic K794Q mutation also showed enhanced binding of KINDLIN2 compared to the non-modified peptide. but still had less negative dG than the peptide carrying the longer and positively charged arginine residue (K794R).

Overall, molecular modeling and biochemical analyses suggested that the elimination of the positive charge in the acetylated lysine side-chain was not a critical determinant for the tight binding of KINDLIN2 to acetylated Integrin-β1. Instead, the structure of the acetylated lysine, the aromatic nature of the N-alkylacetamide group, or the steric and hydrogen accepting properties of the carbonyl group might be important for modulation of KINDLIN2 binding.

### Acetylation of Integrin-β1 at K794 resists cell contraction-induced adhesion remodeling

**Figure 6:**
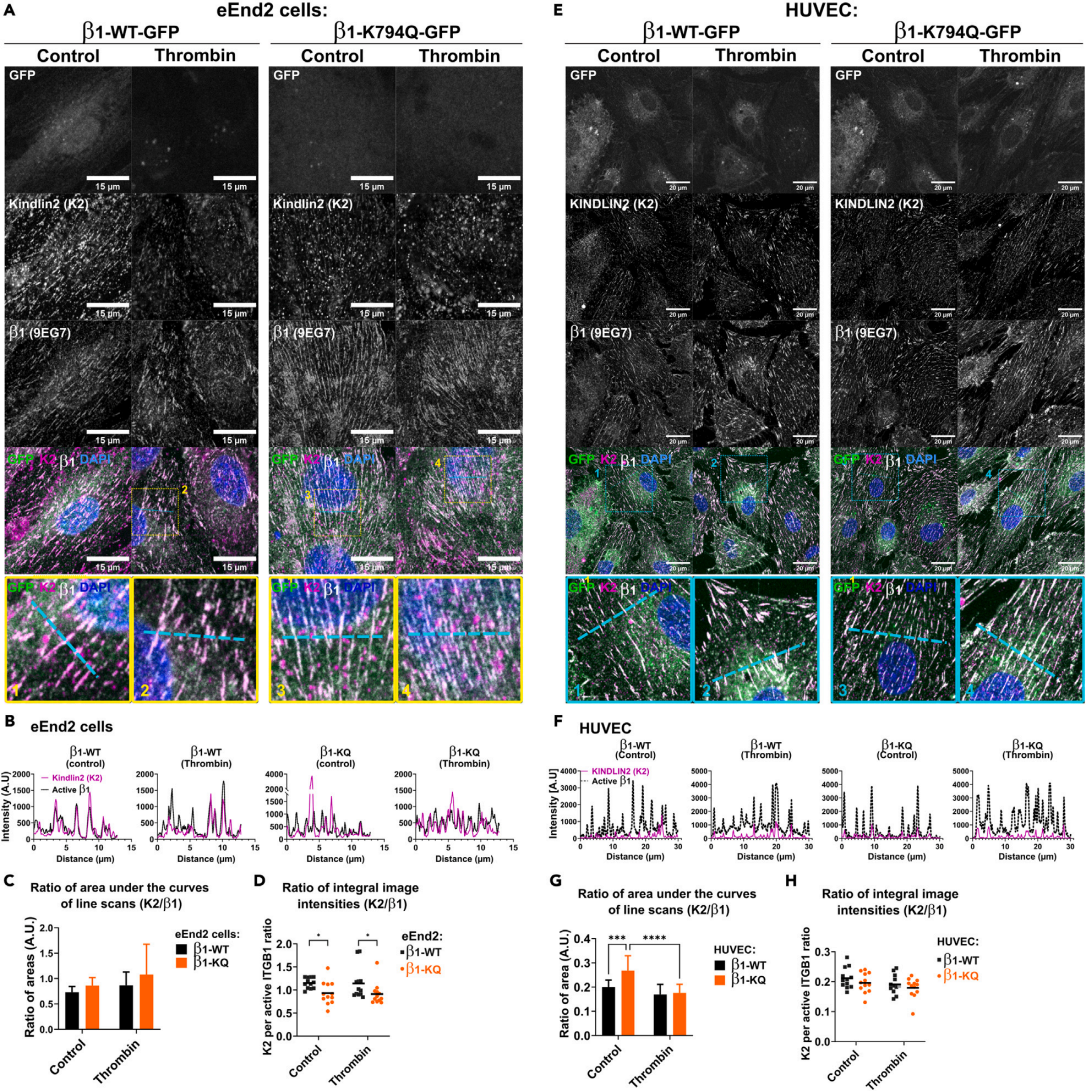
Analysis of KINDLIN2 recruitment to cell-matrix adhesions in HUVEC (A) Analysis of KINDLIN2 recruitment to cell-matrix adhesion sites and the influences of the constitutive expression of acetylation mimetic β1-K794Q, as well as Thrombin-induced retraction of mouse eEnd2 cells (A), or HUVEC (E). Cells expressing either β1-WT-GFP or β1-K794Q-GFP were stimulated or not (control) with Thrombin and KINDLIN2 recruitment to adhesion sites was analyzed by confocal microscopy. GFP indicates β1-GFP expression, staining of Integrin-β1 with the clone 9EG7 shows Integrin-β1 adhesions and KINDLIN2 (K2) was detected with a specific antibody. Single channel images (greyscale) of each condition are shown and their merge in color (Green: GFP, Magenta: K2, Gray: activated β1-Integrin and Blue: DAPI). Scalebar = 15 μm. Areas delineated by the dashed squares (1–4, yellow for eEnd2 and cyan for HUVEC) in merge pictures are zoomed and shown below the specific image to highlight KINDLIN2 at adhesions. A transvers dashed line in each merge was used to show the K2 and β1 intensity profile [(B) for eEnd2 and (F) for HUVEC)]. The intensity profile is shown as function of the distance (μm). Ratio of areas under the curves of KINDLIN2/active β1 at adhesions and the impact of Thrombin treatment are shown in (C) for eEnd2 and (G) for HUVEC. The mean ± sd, *n* = 12 ratio of area under the curve. The difference was indicated with stars when significant with *p*-values <0.05 in two-way ANOVA test. Ratio of K2/active Integrin-β1 (9EG7) intensities from the entire confocal images comprising adhesions and the basal membrane of eEnd2 cells (D) or HUVEC (H) expressing β1-GFP (WT vs. K794Q) and under Thrombin-treatment or not as indicated. Each dot represents the ratio of an image and the line show the mean of a given condition. The significance is indicated with stars when *p*-value ≤0.05 in unpaired t-test. The results are representative of two independent experiments. Related data are included in Figures S7 and S9.

Since KINDLIN2 binding to Integrins was modified by Integrin acetylation, we asked whether rapid changes in local mechanical tension could be absorbed by this linkage. Thus, we analyzed recruitment of KINDLIN2 to cell-matrix adhesions of HUVEC and eEnd2 cells expressing wildtype and β1-K79Q-GFP mutant Integrin, before and after Thrombin-treatment. As shown in Figure 6A for eEnd2 cells, KINDLIN2 staining colocalized with active β1-Integrin at adhesions in both types of β1-GFP expressing cells. Thrombin treatment of cells expressing β1-WT-GFP induced the remodeling of fibrillar adhesions into peripheral focal adhesions. In contrast, Thrombin treatment did not induce remodeling of dense and thin adhesions in cells expressing β1-K794Q-GFP. In order to determine the amount of KINDLIN2 recruited into the cell-matrix adhesion sites, we analyzed the intensity profiles of KINDLIN2 and the active Integrin-β1 along a line crossing several adhesions. These profiles confirmed the overlap of KINDLIN2 staining with that of β1-WT, and β1-K794Q in the absence or presence of Thrombin (Figure 6B). To further evaluate the amount of KINDLIN2 per active Integrin-β1, we established the ratio of the area under each of the intensity curves and obtained similar values between Integrin-β1 and β1-K794Q expressing cells (Figure 6C). Interestingly, in HUVEC stably expressing β1-K794Q, a higher intensity of KINDLIN2 was detected in 9EG7 positive adhesions compared to cells expressing β1-WT (Figures 6E–6G). The calculation of the ratio of the total fluorescence intensities of KINDLIN2/β1 of the confocal images also revealed a similar ratio of KINDLIN2 with activated Integrin-β1 indicating the consistency of the KINDLIN2 recruitment to cell-matrix-bound Integrin-β1 (Figures 6D and 6H).

Interestingly, eEnd2 cells expressing β1-K794Q-GFP also presented accumulation of KINDLIN2 and active Integrin-β1 next to cell-cell junctions (Figure S9). Thus, an enhanced KINDLIN2 recruitment to constitutively acetylated Integrin-β1 appears to increase basal, but potentially immature adhesions, but induces also laterally positioned Integrin-adhesions in proximity to cell-cell junctions. These latter adhesions may contribute to the modified junctional organization of EC, expressing acetylated Integrins with enhanced KINDLIN2 binding affinity.

### Structural basis for the enhanced KINDLIN2 binding of acetylated-β1 and its mimetic mutations

**Figure 7:**
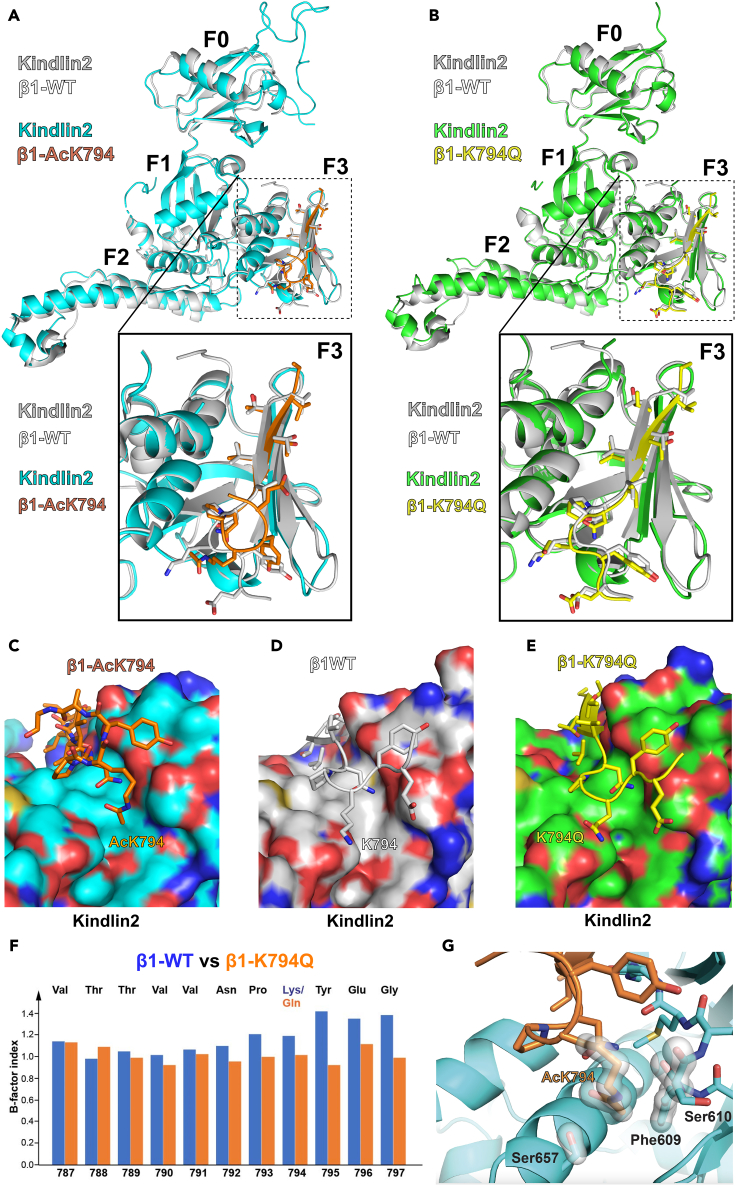
Structural analysis of Integrin-peptide/Kindlin2 complexes (A) Superimposition of the structures of the mouse Kindlin2 fusion protein with the mouse β1-Integrin peptide (PDB: 5xq0; Kindlin2 and β1 in gray), and the co-crystalized structure of the acetylated Integrin β1 peptide (orange) together with Kindlin2 (cyan). An enlarged view of the F3 domain, shows the similar Kindlin2 association of the Integrin peptide prior to the NPKY-motif and the upward turned Lys-acetylated β1-Integrin peptide (orange). (B) Superimposition of the structure of the Kindlin2-β1-peptide fusion protein (PDB: 5xq0, gray) and that of the fusion protein of Kindlin2 with the β1-peptide carrying the K794Q-mutation (Kindlin2 in green, β1-K794Q in yellow). The enlarged view of the insert in B, shows that compared to the β1-WT peptide, the β1-K794Q peptide (yellow) is positioned closer to the Kindlin2 NPXY binding pocket, therefore explaining the enhanced affinity of the β1-K794Q peptide for Kindlin2. (C–E) Comparison of the peptide and side-chain conformation of the acetylated Integrin peptide (NP^Ac^KY)(C), with the non-modified β1-Integrin peptide (NPKY)(D), and the acetyl-mimetic form of the β1-Integrin peptide (NPQY)(E). (F) B-factor index for the respective B-factors of the wildtype and K794Q-mutant Integrin sequences demonstrating stabilization of the c-terminal KYEG-peptide sequence by the introduction of the acetyl-mimetic mutation (QYEG). (G) Analysis of side-chain interactions of the acetylated lysine residue with the Integrin-binding pocket of Kindlin2. Please note the close contacts of the methyl-group with Phe609 and Ser610 at the base of the peptide binding pocket, as well as H-bond formation between the carbonyl-group of the acetyl-lysine with the Ser657 sidechain. Due to the lateral shift of the acetyl-lysine side chain, and tyr-side chain anchorage to Kindlin2, the integrin peptide curls upwards.

To elucidate the structural basis of the enhanced binding of KINDLIN2 with either acetylated Integrin-β1 or its mimetic mutations, we attempted the co-crystallization of mouse Kindlin2 with a free mouse Integrin-β1 peptide bearing an acetylated K794 (AcK794) residue. Despite previous unsuccessful attempts when co-crystallizing Kindlin2 with wildtype Integrin-peptides,^42^ this strategy resulted in co-crystals of Kindlin2 and the acetylated Integrin-β1 peptide that diffracted to 2.04 Å (Figures 7A and Table 1). This is consistent with the enhanced interaction with Kindlin2 when K794 is acetylated in the Integrin-β1 peptide (Figure 6C). The structure reveals that acetylated K794 of Integrin-β1 is making close contacts with the β4-β5 loop and α1 of the F3 domain of Kindlin2. Moreover, the C-terminal end of the Integrin-peptide curls upwards, owing to a lateral shift of the acetyl-lysine side chain, and repositioning of the succeeding Tyr795-side chain anchorage to Kindlin2 as a result (Figure 7C). We also determined the crystal structures of Kindlin2 fused to the β1-K794Q peptide at 2.49 Å resolution (Figure 7B and Table 1). The structure revealed that the backbone of the β1-K794Q peptide is positioned closer to Kindlin2 compared with the wildtype β1 peptide (Figure 7B),^47^ which is consistent with the enhanced binding observed in the pulldown assay.

The surface representation of Kindlin2 indicated that the β1-K794Q peptide (Figure 7E) is bound closer to the Kindlin2 F3 subdomain than the wildtype β1 peptide (Figure 7D), and that the β1-AcK794 peptide fits the Integrin binding groove of Kindlin2 more snugly than the wildtype peptide (Figure 7C).

Moreover, we analyzed the bound peptides for B-factors of the residues in wildtype versus K794Q peptides from the Kindlin2/integrin fusion structure^47^ While residues at the amino-terminal half of the two peptides exhibit similar B-factors, residues near the K794Q site at the carboxyl-end of the mutant peptide exhibit lower B-factors compared with the corresponding residues in the wild-type peptide (Figure 7G). Since B factors represent the atomic displacement and mobility of the side chain, this result further supports the notion that the K794Q mutation in β1 peptide enhances its binding with KINDLIN2. In addition, proximity analysis confirms that the Acetyl-K794 side chain makes contacts with side chains of F609 and S657, and the backbone of S610 (Figure 7H). It is worth noting that the helical conformation at the free carboxyl-end of the β1-AcK794 peptide is similar to that of a previously reported PIP5K-derived peptide bound to the TALIN1 F3 domain,^43^ wherein the two modified peptides exhibit higher affinity than the wildtype peptides.

Our analyses revealed the structural basis for the enhancement of KINDLIN2 binding to acetylated-β1 and its mimetic mutants. The KINDLIN2-dependent signaling triggered by the enhanced binding of KINDLIN2 to acetylated Integrin-β1, may thus provide a consistent mechanistic explanation for the observed phenotype in EC expressing the acetylation-mimetic K794Q mutant of Integrin-β1.

### KINDLIN2 overexpression triggers integrin-dependent pathways and alters endothelial junctions

**Figure 8:**
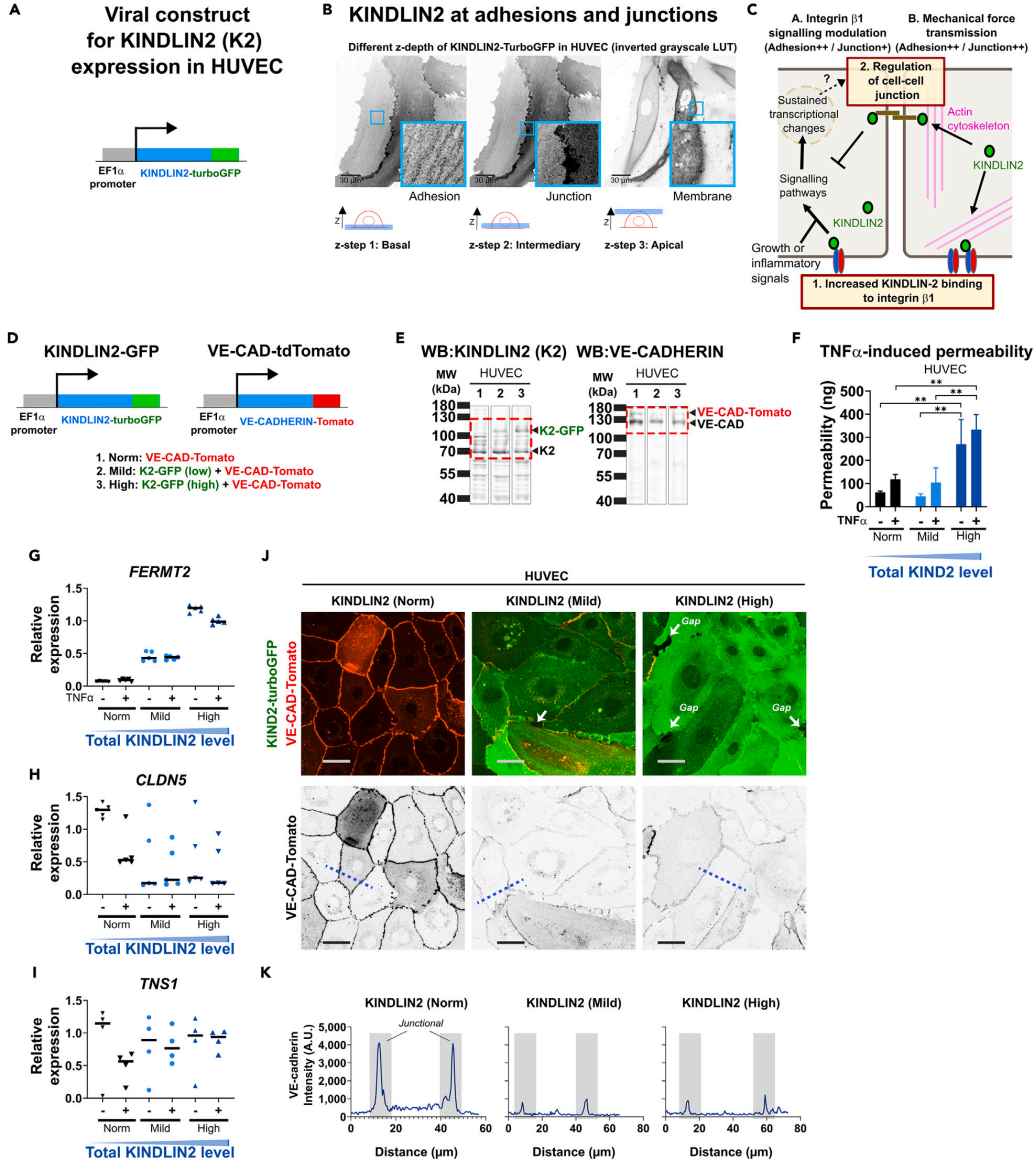
KINDLIN2 expression fosters signaling pathways dependent on Integrin-β1 function and deregulates endothelial junctions (A) The chimeric human KINDLIN2-turboGFP was expressed under the control of the EF1α promoter in HUVEC through a lentiviral construct. (B) Localizations of KINDLIN2-TurboGFP in endothelial cells. Inversed LUT pictures of different depth of z-stacks of HUVEC expressing KINDLIN2-TurboGFP showing its localization at the basal cell surface, at cell-cell junctions and dorsal plasma membrane. The scheme below the pictures indicates the approximative z-level at which the picture was taken. Scalebar = 50 μm. The area contained in the zoom-in shows the KINDLIN2-TurboGFP accumulation at adhesions, cell junctions and apical cell membrane. (C) Illustration of the hypothetic implications of KINDLIN2 in the coordinated control of cell adhesion and junctions. The model shows two functions of the cellular KINDLIN2: 1) playing a role in the mechanical force transmission between the actin cytoskeleton and adhesive complexes at cell adhesions, as well as cell junctions, positively strengthening both adhesions and junctions; 2) and a second role in enhancing Integrin-β1 adhesion and intracellular signaling pathways. In the second case, the coordination with growth or inflammatory signals may synergize or antagonize gene expression programs and barrier function. Related data are included in Figure S10. (D) HUVEC were transduced to express either the chimeric VE-CADHERIN-tdTomato alone (Norm: endogenous level of KINDLIN2) or in presence of different levels of KINDLIN2-turboGFP (Mild: endogenous KINDLIN2 plus low amount of the chimeric K2-turboGFP, High: endogenous KINDLIN2 plus high amount of K2-turboGFP), all under the transcriptional control of EF1α promoter. (E) Western blotting confirming the expression of the chimeric KINDLIN2-turboGFP (Left panel) and VE-cadherin-tdTomato (right panel), their endogenous versions are also visible on the respective blots, all bands appear at the expected sizes. Related figure at Figure S11. (F) Impact of KINDLIN2 expression level on the permeability of HUVEC upon TNFα challenge. The leakiness of HUVEC monolayer to Dextran-FITC (MW:150′000) was assayed by quantifying the amount of leaked dye (ng) from a transwell compartment to a bottom chamber. Data presented as mean ± sd. Significance indicated by stars when *p*-value ≤0.05 in a two-way ANOVA test. (G–I) Analysis by qPCR of the influence of cellular KINDLIN2 level on genes expression in HUVEC under inflammation. The mRNA levels of *FERMT2* (G), *CLDN5* (H) and *TNS1* (I) are shown. Data were combined from 4 to 5 independent qPCR experiments. Each dot represents the mean value found in an independent qPCR and a line indicates the median value of all experiments. Related data are included in Figure S12. (J) Influence of the expression levels (Norm, mild and high) of KINDLIN2 on endothelial junctions in HUVEC monolayer by confocal microscopy. Merge channels is shown in upper panel in color (green: KINDLIN2-turboGFP and red: VE-CAD-Tomato), VE-CADHERIN is shown alone in reverted greyscale. Scalebar = 35 μm. (K) Intensity profiles of VE-CAD-Tomato along the blue dashed lines drawn in the pictures of VE-CAD-Tomato alone in the panel J. In some experiments, peaks of VE-CAD-Tomato were strikingly reduced, and gaps were frequent at cell junctions of HUVEC expressing high levels of KINDLIN2-turboGFP.

Since acetylation of Integrin-β1 enhances KINDLIN2 binding and adhesion formation, permanent acetylation of Integrin-β1 could therefore affect intracellular signaling dependent on KINDLIN2. To better understand how increasing KINDLIN2 expression support Integrin-β1 signaling, we expressed KINDLIN2-turboGFP chimera under the control of EF1α in HUVEC (Figure 8A). HUVEC expressing KINDLIN2-turboGFP showed localization of KINDLIN2 in adhesions, cytoplasm, cell membrane and cell junctions (Figure 8B), consistent with previous studies.^21^ These multiple locations are in line with a direct implication of KINDLIN2 in mechanical force transmission and signaling crosstalk between cell-matrix and cell-cell junctions to control barrier functions (Figure 8C). Notably, the overexpression of KINDLIN2-turboGFP led to increased cell surface interaction with ECM manifested by larger size of the spread cells (not shown). We therefore stimulated HUVEC signaling with the inflammatory cytokine TNFα, expected to modify the balance between cell-matrices and cell-cell junctions. HUVEC expressing three different levels of KINDLIN2 (Norm: endogenous only, Mild: endogenous plus low level of KINDLIN2-GFP, and High: endogenous plus high level of KINDLIN2-GFP) were used in this experiment.

The analysis of intracellular signaling induced by KINDLIN2 overexpression showed the constitutive activation of the MAPK signaling pathway, similar to the observations with the expression of the K794Q-mutant of Integrin-β1 (Figure S10). Treatment of cells with TNFα, known to induce an inflammatory state in EC, was able to enhance MAPK signaling in control cells but not in cells expressing higher levels of KINDLIN2, which showed constitutively high level of phospho-ERKs.

To further evaluate the influence of KINDLIN2 on EC junctions and barrier formation, we analyzed VE-CADHERIN-Tomato localization in HUVEC presenting different levels of KINDLIN2 (Norm, Mild and High; Figure 8D). The expression of endogenous proteins (KINDLIN2 and VE-CADHERIN) as well as the chimeric ones (KINDLIN2-GFP and VE-CADHERIN-Tomato) was confirmed by western blotting (Figures 8E and S11). We analyzed endothelial barrier functions by quantifying the leakage of FITC-dextran (150 kDa) through HUVEC monolayer upon TNFα challenge. The basal permeability of HUVEC was high in cells with the highest level of KINDLIN2 (Figure 8F). To understand the cause for this lack of junctional organization, we analyzed the expression of several genes involved in controlling cell junctions and adhesions during inflammation by qPCR (Figures 8G–8I and S12). The principal component analysis of the data revealed a clear effect of both KINDLIN2 expression level and TNFα-treatment, as well as the relationship between different gene expression patterns (Figures S12A and S12B). The quantification of *ICAM1* levels indicated a proper inflammation response in TNFα-treated HUVEC (Figure S12C). Furthermore, qPCR confirmed the enhanced amount of transcripts of KINDLIN2 (*FERMT2*) in KINDLIN2 overexpressing cells, which also showed a reduction of CLAUDIN5 transcripts independently of TNFα-treatment (Figures 8G and 8H). Similarly, TNFα-treatment decreased *TNS1* in normal cells, the reduction was however largely prevented by KINDLIN2 expression in a dose-dependent manner (Figure 8I). In contrast, KINDLIN2 expression had no effect on TNFα-induced abrogation of *TNS2* transcripts (Figure S12H). Interestingly, the combination of the expression of KINDLIN2 and TNFα-treatment showed a synergistic increase of the ECM protein Tenascin-C (Figure S12J), further suggesting a role for KINDLIN2 in ECM remodeling during inflammation.

These results demonstrate that KINDLIN2 induces profound and sustained changes to cell-ECM adhesions, as well as cell-cell junctions leading to increased permeability in steady state and during inflammation. Consistently, the analysis of cell junctions by confocal microscopy showed a reduction of VE-CADHERIN at the junctions of KINDLIN2-overexpressing cells (Figures 8J and 8K). This was associated with the appearance of several gaps between EC. Altogether, these data demonstrated a critical contribution of KINDLIN2 in the regulation of EC junctions through modulations of Integrin-dependent signaling pathways such as MAPK, and sustained changes of transcripts coding for several adhesion and junctional proteins. The similarity of phenotypes in KINDLIN2 overexpressing cells with those of permanently acetylated Integrin-β1-expressing cells, suggest a common regulatory mechanism.

## Discussion

**Figure 9:**
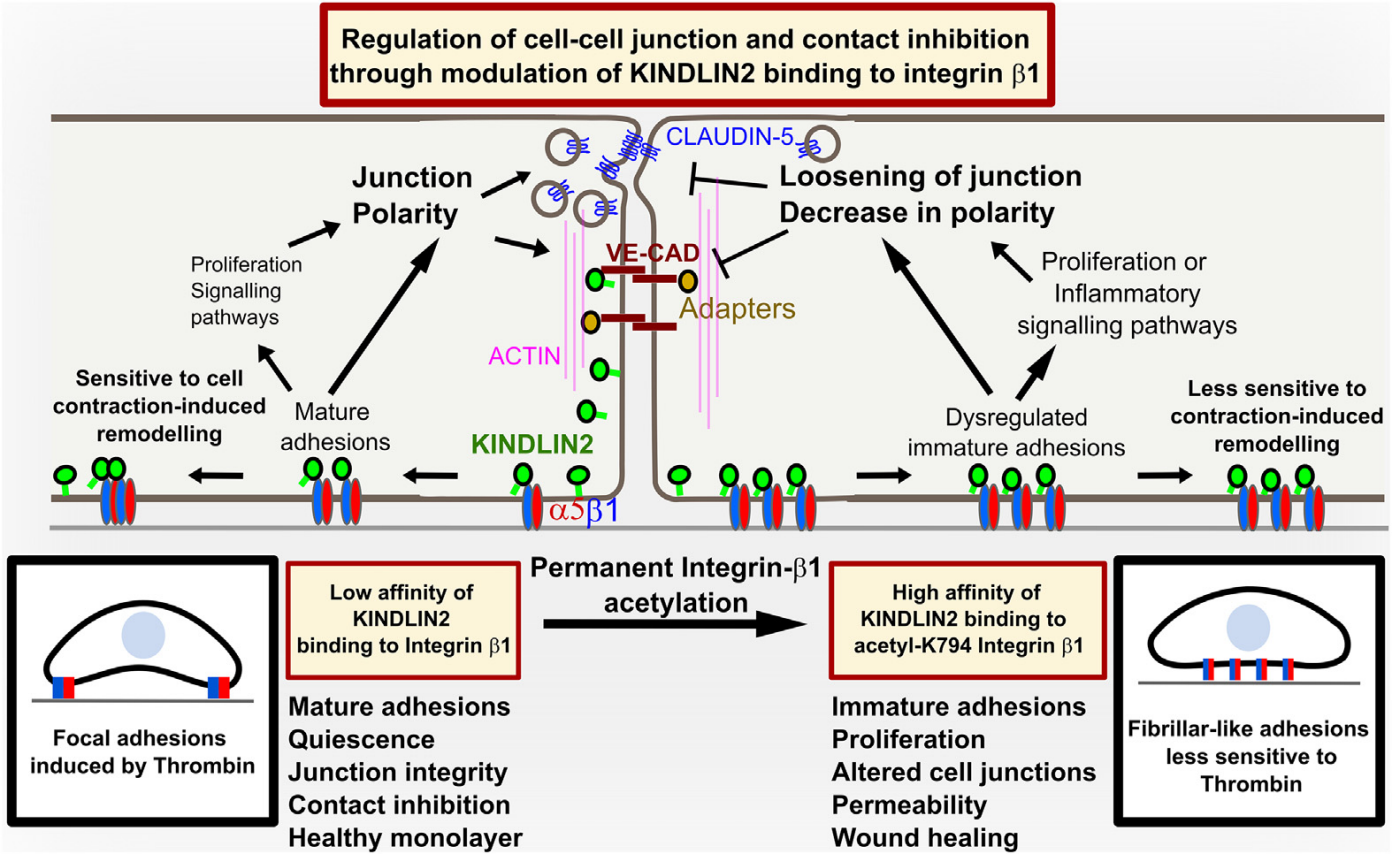
Model of the regulation of endothelial cell junctions, contact inhibition, and cell-matrix adhesion formation by Integrin-β1 acetylation: Acetylation of Integrin-β1 regulates KINDLIN2 binding to Integrin-β1, inducing transcriptomic control of the expression of adhesion, junctional and proliferation-regulating genes In endothelial cells at confluence, Integrin-β1 might not be acetylated, allowing maturation of Integrin-adhesions, which reduces Integrin β1-dependent signaling pathways such as MAPK, PI3K and SRC activities because of reduced KINDLIN2 binding to basal integrins. In turn, this reduced Integrin signaling allows higher expression levels of junctional proteins, resulting in stabilization of cell-cell interactions, enhanced polarity and integrity of the monolayer leading to enhanced barrier function. In response to Thrombin treatment and increased cell contractility, effective cell edge retraction and a remodeling of central adhesions to peripheral focal adhesions (left part of the scheme) is induced. During wound-healing, Integrin β1 acetylation may provide enhanced KINDLIN2 recruitment and Integrin-dependent signaling to induce migration and proliferation until the monolayer is re-established. In contrast, when confluent endothelial cells are stimulated with acetylation mediating pathways, KINDLIN2 is recruited and Integrin β1-dependent signaling pathways such as MAPK and PI3K activities are enhanced. In the specific experimental case of permanent acetylation mimetism by the constitutive expression of the K794Q-mutant of Integrin-β1, KINDLIN2 is recruited with higher affinity leading to the formation of dysregulated immature adhesion. This leads to the dysregulation of endothelial cell junctions and loss of contact inhibition of proliferation. This is further decreasing the expression of junctional proteins while favoring cell-matrix adhesions and proliferation through maintaining the high transcript levels of the Integrin adaptor proteins as well as cell cycle-mediating cyclins. In response to Thrombin treatment of cells expressing permanently acetylated Integrin-β1, cell-matrix adhesions fail to remodel, maintaining thin elongated central adhesions despite the disruption of cell junctions (right part of the scheme).

In this study, we show that Integrin-β1 acetylation modulates the formation of cell-ECM adhesions, barrier function, cell polarity and proliferation in EC by promoting the recruitment of KINDLIN2 to Integrin-β1. KINDLIN2 binding fosters Integrin-dependent signaling pathways and subsequently modulates the transcription of several genes involved in cell junction organization, cell-matrix adhesions, polarity and proliferation in EC (Figure 9).

Previous studies utilizing fibroblasts showed phosphorylation on Tyr- and Ser/Thr-residues in the NPXY-, or inter NPXY-region regulating Integrin-β1 activation or modulating cell adhesion, notably by affecting adapter protein binding in interphase, during mitosis (T787/T788) and during Integrin-dependent invasion (S785).^22^^,^^48^^,^^49^^,^^50^ However, the role of other post-translational modifications of the cytoplasmic tail of Integrin-β1 remains not well understood. In this study, we focused on the residue Lys794 of Integrin-β1 that was found acetylated by proteomic analysis.^24^ As Lys-acetylation prevents ubiquitination, lysine acetylation could modify protein turnover and cell surface expression. Lysine acetylation is also involved in modifying protein-protein interactions.^51^ We studied these different possibilities by using lysine-to-glutamine mutation of residue 794 of Integrin-β1 (K794Q) to structurally mimic the acetylated lysine residue, thus creating a model in which a significant proportion of Integrin-β1 in EC were in a permanently acetylated state. Previously, the expression of β1-GFP(K794Q) in β1-deficient GD25 cells enhanced fibronectin fibrillogenesis,^25^ but its effects on transcription and signaling were not analyzed. Here we show that expressing permanently acetylated Integrin-β1 imposes the formation of dense and thin fibrillar-like adhesions in the basal area of EC and modifies the transcriptomic profile of EC by increasing transcript levels of genes such as those of cyclins, CDKs or Mdm2, simultaneously promoting cell proliferation. Consistently, the β1-GFP(K794Q) expression is sufficient to increase the proliferation of EC such as eEnd2 cells and HUVEC.

In response to high cell density, EC inhibit proliferation to maintain a monolayer through mechanisms involving cell junctions that dampen the activity of growth factor receptors.^30^^,^^52^ β1-GFP(K794Q) expression induced cell proliferation that was less sensitive to EC density and contact inhibition, likely due to deficient cell polarity and anarchic organization of junctional structures. Accordingly, the expression of several polarity genes, such as *Pard3* and those encoding for junctional proteins such as CLAUDIN5 were reduced by β1-GFP(K794Q) expression. Importantly, in the presence of wound-provoked gradients of cell density, EC expressing β1-GFP(K794Q) showed similar proliferation potentials between areas of low and those of high density. This suggests that physiological acetylation of Integrin-β1 may target EC junctional structures in order to induce wound-healing responses, leading to reduced cell-cell contacts, and simultaneously modify the cell proliferation at the site of injury. In contrast, maintaining high levels of β1-Integrin acetylation at cell confluency may cause pathological consequences, negatively affecting monolayer integrity, and reducing contact inhibition of proliferation and barrier function. Our data support the notion that an altered metabolic state of EC may have a deleterious impact on EC junctions and barrier functions through constitutive acetylation of Integrin-β1, advocating the development of new tools quantifying Integrin-acetylation in pathological conditions.

Here we show that acetylation of Integrin-β1 enhances the affinity of adapter proteins such as KINDLIN2 for Integrin-β1 cytoplasmic tails, potentially relevant for the observed changes in cell behavior. Our structural study revealed that acetylation of K794 of the distal NPXY-motif of Integrin-β1 enhances interaction with the Integrin-binding pocket of KINDLIN2. Consistently, in-silico dynamic modeling of the complex as well as the structure of the KINDLIN-Integrin complex obtained with X-ray crystallization suggested tighter KINDLIN2 binding with acetyl-K794 and acetyl-lysine mimetic peptides (K794Q and K794R) as compared to the non-modified peptide. This was supported by the biochemical pulldown assays showing that acetyl-K794, K794Q and K794R peptides enhanced KINDLIN2 binding.

The similarity of KINDLIN binding between the neutral K794Q and the positively charged but bulkier K794R acetyl-lysine mimetic sidechains suggested that the charge neutralization of K794 was not the main determinant for increased KINDLIN2 affinity. Instead, the replacement of the Lys in the cytoplasmic tail of β7-Integrin by Arg (NPRF), may be a natural example of a permanent Integrin acetylation and a mechanism to enhance KINDLIN binding, and to stabilize adhesion of β7-Integrin expressing leukocytes in peripheral organs, such as the gut.^53^

Currently, we can only speculate why the expression of the β1-K794Q mutant results in enhanced fibronectin fibrillogenesis, while the K794R prevented fibronectin fibrillogenesis.^27^ It is possible that independently of increasing KINDLIN2 binding affinity, Integrin acetylation may control additional adapter binding events, affecting the fate of Integrin-dependent adhesion and signaling. Nonetheless, the biochemical affinity data validated the use of the K794Q mutation as a relevant model to recapitulate the effect of Integrin-β1 acetylation in cells.

Integrin capture by the PH-domain-dependent membrane-associated KINDLIN2 is a key event in the induction of cell adhesion, even prior to TALIN1-association.^52^ Acetylation of the distal NPKY-motif of Integrin-β1 may foster the recruitment of KINDLIN2, thereby priming the Integrins for activation and initiation of cell-matrix adhesions. Consistently, the expression of β1-GFP(K794Q) leads to the formation of dense and thin adhesions, likely reflecting increased KINDLIN2 enrichment compared to the β1-WT-GFP in EC. The high density and immature nature of adhesions in β1-K794Q-GFP-expressing cells further indicates that Integrin-β1 acetylation might be a transient event occurring in early phases during adhesion formation that might need to be reversed to avoid too dense adhesion arrays and the deleterious consequences associated with a permanent Integrin-β1 acetylation. Alternatively, constitutive and permanent acetylation of Integrin-β1 could induce KINDLIN2-mediated activation of Integrin-β1 outside of cell-ECM adhesions, which would reduce KINDLIN2 availability in peripheral adhesions. Accordingly, the amount of free KINDLIN2 available for Integrin-β1 activation in the cell edge may be reduced in β1-K794Q-GFP-expressing EC. We observed that elevating KINDLIN2 binding to acetylated Integrin-β1 will cause alteration in adhesions, initiating a dramatic dysfunction of EC.

Several studies revealed a critical role of Integrin-β1 in blood vessel development and endothelial barrier functions through sustained control of gene and protein expression patterns.^5^^,^^6^ It was recently proposed that Integrin-β1 is required for p120-catenin binding to VE-CADHERIN and for the stability of blood vessels.^6^ In contrast, other studies have shown that the function of Integrin-β1 can also negatively influence endothelial barrier and induce permeability, although the precise mechanism remained elusive.^10^^,^^54^ We propose that by enhancing KINDLIN2 binding, acetylation of Integrin-β1 can constitute the main cause that links the function of Integrin-β1 in adhesions to endothelial junction stability or destabilization, through supporting integrin-dependent signaling and orchestrating profound changes in gene expression profiles. Although KINDLIN2 interacts directly with junctional complexes,^20^^,^^21^^,^^36^ its recruitment to the Integrin-β1 tail is essential for the initiation of cell-matrix adhesions and this is proposed to control EC junctions. In our study we found that the permanent acetylation of Integrin-β1 shared several phenotypes with the overexpression of KINDLIN2 in EC. Indeed, the expression of both β1-K794Q-GFP and KINDLIN2 similarly increased cell adhesion area, caused constitutive activation of MAPK signaling, and provoked similar changes in gene expression of junctional proteins, causing a reduction in junction organization and enhanced permeability. Previously, MAPK signaling was shown to be induced by Integrin-β1 to promote cell division.^15^^,^^17^^,^^55^ In contrast, MAPK signaling needs to be dampened during formation of cell-cell junctions to favor the contact-induced arrest of EC proliferation.^30^^,^^56^ By augmenting KINDLIN2 recruitment and sustained KINDLIN2-dependent induction of MAPK signaling, EC will be responsive to Integrin-β1 acetylation mediating pathways that can cause the loss of monolayer organization and resistance to contact inhibition of proliferation.

Previously, the mechano-sensing Hippo-YAP pathways was proposed to control proliferation arrest of several cell types at confluence.^57^ Although the function of Integrin-β1 can control the Hippo-YAP pathway,^55^ the forced acetylation of Integrin-β1 did not seem to induce YAP activation as per our transcriptomic data. Indeed, most of the genes that were reported to be transcriptionally induced after YAP nuclear translocation were not changed by the expression of acetylation mimetic K794Q mutant of Integrin-β1.^32^ This proposes that Hippo and integrin-dependent signaling pathways can act independently on cell-cell junctions and the cell-matrix interface, respectively. Therefore, Integrin-β1 appears to be mainly implicated in the homeostasis of the EC monolayer by ensuring cell-ECM interaction^56^ and by modulating the expression of genes contributing to cell polarity such as *Pard3 and Jam3*.^5^^,^^58^^,^^59^

Modifying the inter- and intracellular tensions by the Rho family GTPases can drastically change the architecture and organization of cell junctions and adhesions. Consistently cell contractility was shown to suppress podosomes.^60^ Thus, altered activities of Rho GTPases could be expected to occur in EC presenting permanent integrin acetylation. However, after Thrombin-treatment, which is a strong inducer of Rho activity, EC expressing β1-K794Q-GFP showed minor modulations of cell-matrix adhesions, while junctional disruption and cell retraction were effective. This indicates that cell-matrix adhesions in β1-K794Q- GFP-expressing cells were not under tension and hence only slightly affected by Rho GTPase activity.

Our data and several recent works indicate an important role of KINDLIN2 in mediating the adhesion formation and cell junction destabilization induced by Integrin-β1 acetylation. Under inflammatory conditions, the overexpression of KINDLIN2 prevented the transcript of TENSIN1 (*TNS1*) from being downregulated by TNFα, providing a potential link to adhesion reinforcement, fibronectin fibrillogenesis and ECM-deposition.^10^^,^^38^^,^^51^ The increased TENSIN1 binding to Integrin-β1 adhesions was recently proposed to be associated with altered EC junctions and permeability although the precise mechanisms were not resolved.^10^^,^^54^ Here we found that in parallel to maintaining the amount of *TNS1* transcripts upon inflammation, the high cellular levels of KINDLIN2 abrogated the expression of CLAUDIN5 gene (*CLDN5*). This decrease of *CLDN5* by increased KINDLIN2 was consistent with *CLDN5* being down-regulated by the expression of acetylation mimetic mutant of Integrin-β1 that negatively affected EC junctions. At this point however, it is not clear whether *CLDN5* down-regulation was induced by any action directly mediated by TENSIN1. Nevertheless, it demonstrates that the acetylation of Integrin-β1 and the subsequently increased binding of KINDLIN2 might change the composition of the adapter proteins populating Integrin-β1 adhesions and thereby impact cell-cell junctions by modulating transcriptionally the levels of their components such as CLAUDIN5. Here, it is noteworthy that we used KINDLIN2 overexpression to amplify cell-ECM adhesions and Integrin-β1/KINDLIN2-dependent signaling, as an experimental approach to better understand the consequences of Integrin-β1 acetylation and KINDLIN2 recruitment. Consistently, the overexpression of KINDLIN2 increased adhesion formation and the surface of cell-ECM interaction (not shown). In addition, KINDLIN2 overexpression phenocopied several features of Integrin-β1 permanent acetylation, such as favoring adhesion formation, constitutive induction of MAPK signaling, regulation of the expression of junctional proteins such as CLAUDIN-5 and disruption of barrier function leading to increased permeability.

The importance of Integrin-β1 was established for both the maintenance and the destabilization of EC junctions in several previous studies, although conciliating mechanisms were missing. Here we propose that controlled acetylation/deacetylation of Lysine 794 of Integrin-β1 is such a determining mechanism that could modify the composition of Integrin-β1 adapter complexes leading to transcriptional modulation of components of endothelial cell-cell junctions. Therefore, the level of this post-translational modification may be a decisive factor to maintain or to destabilize EC junctions. This opens new avenues for developing novel and concrete approaches to control EC quiescence and barrier function in several pathologies through detection and targeting of Integrin-β1 acetylation.

### Limitations of the study

In this study, we analyzed the role of acetylated Integrin-β1 in the regulation of the proliferation and barrier function of EC. However, altered acetylation by metabolic changes may affect many different cell types and pathological situations such as cancer, not covered by this study. Other post-translational modifications such as malonylation or succinylation that are also modulated by the cell metabolic state were not assessed. In addition, by expressing a constitutive acetylation-mimetic mutation, we did not recapitulate the dynamic aspects of this post-translational modification. Therefore, the regulatory pathways designed to modulate the acetylated states of proteins may not be properly represented or cannot be analyzed in this study. Structural and biochemical analysis have been focused on the KINDLIN2 integrin adapter protein; however, we cannot exclude that Integrin-β1 acetylation may also influence the binding behavior of other integrin adapters.

## STAR★Methods

### Key resources table

**Table:** 

REAGENT or RESOURCE	SOURCE	IDENTIFIER
**Antibodies**		

Polyclonal rabbit anti-Kindlin2	Sigma-Aldrich	Cat# K3269, RRID:AB_10603491
monoclonal rat anti- mouse VE-cadherin (clone 11D4.1)	BD Biosciences	Cat# 555289, RRID:AB_395707
Polyclonal goat anti VE-cadherin (C19)	Santa Cruz Biotechnology	Cat# sc-6458, RRID:AB_2077955
Peroxidase-AffiniPure Goat Anti-Rabbit IgG (H + L) (min X Hu,Ms,Rat Sr Prot)	Jackson ImmunoResearch Labs	Cat# 111-035-144, RRID:AB_2307391
Horseradish peroxidase conjugated donkey anti-rabbit IgG	Santa Cruz Biotechnology	Cat# sc-2020, RRID:AB_631728
Goat anti-Rat IgG (H + L) Cross-Adsorbed Secondary Antibody, Alexa Fluor™ 555	Thermo Fisher Scientific	Cat# A-21434 (also A21434), RRID:AB_2535855
Monoclonal mouse anti-GAPDH (clone: 1E6D9)	Proteintech	Cat# 60004-1-Ig, RRID:AB_2107436
Donkey anti-Rat IgG (H + L) Cross-Adsorbed Secondary Antibody, DyLight™ 650	Thermo Fisher Scientific	Cat# SA5-10029, RRID:AB_2556609
Goat anti-Rat IgG (H + L) Cross-Adsorbed Secondary Antibody, Alexa Fluor™ 633	Thermo Fisher Scientific	Cat# A-21094, RRID:AB_2535749
Rat anti-mouse Integrin beta-1 (clone: 9EG7)	This paper	NA
Donkey anti-Rabbit IgG (H + L) Highly Cross-Adsorbed Secondary Antibody, Alexa Fluor™ 555	Thermo Fisher Scientific	Cat# A-31572 (also A31572), RRID:AB_162543
Monoclonal mouse anti-Phospho-p44/42 MAPK (Erk1/2) (Thr202/Tyr204) (E10)	Cell Signaling Technology	Cat# 9106 (also 9106L, 9106S), RRID:AB_331768
Polyclonal rabbit anti-Phospho-Src (Tyr527) Antibody	Cell Signaling Technology	Cat# 2105, RRID:AB_331034
Monoclonal mouse Anti-alpha-Tubulin antibody	Sigma-Aldrich	Cat# T5168, RRID:AB_477579
Monoclonal mouse Anti-Vinculin (Clone hVIN-1)	Sigma-Aldrich	Cat# V9131, RRID:AB_477629
Rabbit phospho-Akt (Ser473) Antibody	Cell Signaling Technology	Cat# 9271 (also 9271S, 9271L, NYUIHC-310), RRID:AB_329825
Monoclonal rabbit anti-Akt (pan) (C67E7)	Cell Signaling Technology	Cat# 4691 (also 4691L, 4691P, 4691S), RRID:AB_915783
Monoclonal rabbit anti-p44/42 MAPK (Erk1/2) (137F5)	Cell Signaling Technology	Cat# 4695 (also 4695P, 4695S), RRID:AB_390779
monoclonal rat anti- mouse VE-cadherin (clone 11D4.1)	BD Biosciences	Cat# 555289, RRID:AB_395707
Polyclonal goat anti VE-cadherin (C19)	Santa Cruz Biotechnology	Cat# sc-6458, RRID:AB_2077955
Peroxidase-AffiniPure Goat Anti-Rabbit IgG (H + L) (min X Hu,Ms,Rat Sr Prot)	Jackson ImmunoResearch Labs	Cat# 111-035-144, RRID:AB_2307391
Horseradish peroxidase conjugated donkey anti-rabbit IgG	Santa Cruz Biotechnology	Cat# sc-2020, RRID:AB_631728
Goat anti-Rat IgG (H + L) Cross-Adsorbed Secondary Antibody, Alexa Fluor™ 555	Thermo Fisher Scientific	Cat# A-21434 (also A21434), RRID:AB_2535855
Monoclonal mouse anti-GAPDH (clone: 1E6D9)	Proteintech	Cat# 60004-1-Ig, RRID:AB_2107436
Donkey anti-Rat IgG (H + L) Cross-Adsorbed Secondary Antibody, DyLight™ 650	Thermo Fisher Scientific	Cat# SA5-10029, RRID:AB_2556609
Goat anti-Rat IgG (H + L) Cross-Adsorbed Secondary Antibody, Alexa Fluor™ 633	Thermo Fisher Scientific	Cat# A-21094, RRID:AB_2535749
Rat anti-mouse Integrin beta-1 (clone: 9EG7)	This paper	NA
Donkey anti-Rabbit IgG (H + L) Highly Cross-Adsorbed Secondary Antibody, Alexa Fluor™ 555	Thermo Fisher Scientific	Cat# A-31572 (also A31572), RRID:AB_162543
Monoclonal mouse anti-Phospho-p44/42 MAPK (Erk1/2) (Thr202/Tyr204) (E10)	Cell Signaling Technology	Cat# 9106 (also 9106L, 9106S), RRID:AB_331768
Polyclonal rabbit anti-Phospho-Src (Tyr527) Antibody	Cell Signaling Technology	Cat# 2105, RRID:AB_331034
Monoclonal mouse Anti-alpha-Tubulin antibody	Sigma-Aldrich	Cat# T5168, RRID:AB_477579
Monoclonal mouse Anti-Vinculin (Clone hVIN-1)	Sigma-Aldrich	Cat# V9131, RRID:AB_477629
Rabbit phospho-Akt (Ser473) Antibody	Cell Signaling Technology	Cat# 9271 (also 9271S, 9271L, NYUIHC-310), RRID:AB_329825
Monoclonal rabbit anti-Akt (pan) (C67E7)	Cell Signaling Technology	Cat# 4691 (also 4691L, 4691P, 4691S), RRID:AB_915783
Monoclonal rabbit anti-p44/42 MAPK (Erk1/2) (137F5)	Cell Signaling Technology	Cat# 4695 (also 4695P, 4695S), RRID:AB_390779

**Bacterial and virus strains**		

NEB® 5-alpha Competent *E. coli*	New England Biolabs Inc.	Cat# C2987
DH5-alpha competent *E. coli*	This paper	NA
BL21(DE3) chemically competent *E. coli*	Thermo Fisher Scientific	Cat# C600003

**Chemicals, peptides, and recombinant proteins**		

LY294002 (IUPAC Name: 2-(4-Morpholinyl)-8-phenyl-4*H*-1-benzopyran-4-one hydrochloride)	Sigma-Aldrich	Cat# 19-142
Tyrphostin 47 (IUPAC Name: (*E*)-2-Cyano-3-(3,4-dihydroxyphenyl)-2-propenthioamide)	Calbiochem	Cat# 658405
Biotinylated Integrin beta 1 peptide pep_wt(K):Biot-GSAKSAVTTVVNPKYEGK	This paper, synthesized by PPR2P platform UNIGE	NA
Biotinylated Integrin beta 1 peptide pep_Ac-K794:Biot-GSAKSAVTTVVNP-K(Acetyl)-YEGK	This paper, synthesized by PPR2P platform UNIGE	NA
Biotinylated Integrin beta 1 peptide pep_Src. (Ac-K):Biot-GSAKNVSYVTGEAK-(Acetyl)-VTKP	This paper, synthesized by PPR2P platform UNIGE	NA
Biotinylated Integrin beta 1 peptide pep_K794R:Biot-GSAKSAVTTVVNPRYEGK	This paper, synthesized by PPR2P platform UNIGE	NA
Biotinylated Integrin beta 1 peptide pep_K794Q:Biot-GSAKSAVTTVVNPQYEGK	This paper, synthesized by PPR2P platform UNIGE	NA
Kindlin2-Integrin beta 1 (K794Q) peptide fusion protein: Kindlin2-LVPRGSGSGSGS-KSAVTTVVNPQYEGK	This paper	NA
Acetylated-Integrin beta 1 peptide KSAVTTVVNPK(Acetyl)YEGK	This paper, synthesized by GeneScript	NA
Mouse VEGF-165 Recombinant Protein, PeproTech®	Thermo Fisher Scientific	Cat# 450-32
Human TNF-alpha Recombinant Protein, PeproTech®	Thermo Fisher Scientific	Cat# 300-01A
Thrombin	Sigma-Aldrich	Cat# T7009
Collagen G	Biochrom GmbH	Cat# L7213
Gelatin	Sigma-Aldrich	Cat# G1393
Fibronectin	EMD Millipore	Cat# FC010
Vitronectin	Vesa Hytönen, Tampere	Pinon et al. JCB 2014 (https://doi.org/10.1083/jcb.201308136)
Laminin-1	Jürgen Engel, Biozentrum Basel	
Endothelial cell growth supplement	Merck Millipore	Cat# 02-102
Heparin sodium grade I-A	Sigma-Aldrich	Cat# H3149
Hydrocortisone	Sigma-Aldrich	Cat# H6909
Ascorbic acid (vitamin C)	Sigma-Aldrich	Cat# 4544
Polybrene®	Santa Cruz Biotechnology	Cat# sc-134220
5-ethynyl-2′-deoxyuridine	Thermo Fisher Scientific	Cat# A10044
Kindlin2	Jinhua Wu	uniprot#: Q8CIB5

**Critical commercial assays**		

Click-iT™ Plus EdU Alexa Fluor™ 647	Thermo Fisher Scientific	Cat# C10635

**Deposited data**		

RNAseq data	NIH GEO platform (https://www.ncbi.nlm.nih.gov/geo/)	GEO code: GSE238019
PDB files of the crystal structures	PDB -Protein Data Bank (https://www.rcsb.org/)	PDB codes: 8TEC and 8TEE
Unprocessed raw data	University of Geneva server Yareta https://yareta.unige.ch	https://doi.org/10.26037/yareta:h3n7tiyim5ej5iitwgnultysqe

**Experimental models: Cell lines**		

HUVEC	Beat A Imhof, UNIGE, Geneva	Sidibe et al. Nature Communications 2018 (https://doi.org/10.1038/s41467-017-02610-0)
eEnd2 cells	Beat A Imhof, UNIGE, Geneva	
HEK293T cells	Beat A Imhof, UNIGE, Geneva	

**Oligonucleotides**		

pCMV6-KINDLIN2_fwd: PCTCGCGAGC CCGCATGGCTCTGGACGGG	This paper	NA
pCMV6-KINDLIN2_rev: GTACCTCGAG CACCCAACCACTGGTAAG	This paper	NA
pWPT-KINDLIN2_fwd: AACACAGGTGTCGTG ACGCGGATCCGCGGCCGCCATGGCTCTG	This paper	NA
pWPT-KINDLIN2_rev: TTATCGGAATTCCCT CGAGGTCGACGTTTAAACTCTTTCTT CACCGGCATCTGCATCC	This paper	NA
pWPT-beta1_fwd: AACACAGGTGTCGTGACG CGGATCCACCATGAATTTACAACCAATTTTC	This paper	NA
pWPT-beta1_rev: TTATCGGAATTCCCTCGAG GTCGACTCTAGACTCATTTTCCCTC	This paper	NA
Quantitative PCR primers	See Table S3	NA

**Recombinant DNA**		

pcDNA3-SAPMAR-ITGB1AK107-WT-GFP	Bernhard Wehrle-Haller	Vega et al.^25^ (https://doi.org/10.3390/cells9030655)
pcDNA3-SAPMAR-ITGB1AK107-K794Q-GFP	Bernhard Wehrle-Haller	Vega et al.^25^ (https://doi.org/10.3390/cells9030655)
pCMV-CFP-KIND2	Bernhard Wehrle-Haller	NA
pCMV6-AC-GFP	Origene	Cat# PS100010
pWPT-GFP	Didier Trono, EPFL, Lausanne	Addgene plasmid # 12255; http://n2t.net/addgene:12255; RRID:Addgene_12255
pWPT-EF1a-KIND2-turboGFP	This paper	Under deposition to Addgene
pWPT-EF1a-B1Awt-GFP	This paper	Under deposition to Addgene
pWPT-EF1a-B1AK794Q-GFP	This paper	Under deposition to Addgene
pWPT-EF1a-VE-CADHERIN-tdTomato	This paper	Under deposition to Addgene
pCAG-VSVG	Arthur Nienhuis & Patrick Salmon	Addgene plasmid # 35616; http://n2t.net/addgene:35616; RRID:Addgene_35616
psPAX2	Didier Trono, EPFL, Lausanne	Addgene plasmid # 12260; http://n2t.net/addgene:12260; RRID:Addgene_12260
pET28a-Kindlin2	Jinhua Wu	uniprot#: Q8CIB5
tdTomato-VE-Cadherin-N-10	Michael Davidson, Florida, University of Florida	Addgene plasmid # 58142; http://n2t.net/addgene:58142; RRID:Addgene_58142

**Software and algorithms**		

GraphPad Prism	GraphPad Software, LLC	v9
Fiji ImageJ software	ImageJ.org	v1.53q (https://doi.org/10.1038/nmeth.2019)
Inkscape	Inkscape developers	v1.3.2
R package edgeR	Bioconductor.org	v3.38.4 (https://doi.org/10.18129/B9.bioc.edgeR)
Gromacs	GROMACS development team	v2019
X-ray Detector Software XDS	MPI for Medical Research, Heidelberg	https://xds.mr.mpg.de/
ClustVis web tool	Metsalu, Tauno and Vilo, Jaak	https://biit.cs.ut.ee/clustvis/ (https://doi.org/10.1093/nar/gkv468)
Phenix	https://phenix-online.org/	version 1.20.1

**Other**		

Dynabeads™ M-280 Streptavidin	Thermo Fisher Scientific	Cat# 11205D

### Resource availability

#### Lead contact

Further information and requests for resources and reagents should be directed to and will be fulfilled by the lead contact, Dr Adama Sidibé (adama.m.sidibe@gmail.com)

#### Materials availability

Materials used in this study are available from the lead contact upon reasonable request.

#### Data and code availability

• The data of the RNAseq (GEO code GSE238019)^61^ was deposited to the NIH GEO platform and can be found at https://www.ncbi.nlm.nih.gov/geo/. The PDB files of the crystal structures (PDB codes: 8TEC and 8TEE) can be accessed at www.rcsb.org. Other data that support the findings of this study are available within the article and its Supplementary Information files. The unprocessed raw data was deposited to University of Geneva server Yareta and can be accessed at https://yareta.unige.ch (https://doi.org/10.26037/yareta:h3n7tiyim5ej5iitwgnultysqe).^62^
• This paper does not report original code.
• Additional data or information is available from the lead contact upon reasonable request

### Experimental model and study participant details

#### Cells

Human umbilical vein endothelial cells (HUVEC), human embryonic kidney HEK293T cells and mouse embryonic endothelial cells eEnd2 cells were gifts from Prof Beat A Imhof (University of Geneva, Geneva). HUVEC and eEnd2 cells were authenticated by profiling human and mouse endothelial cell specific antigens by flow cytometry. These cells contain lots of both male and female origins invariably. No gender influences of endothelial cell origin were established on cell adhesion or barrier functions. All cells were verified for mycoplasma contamination. HUVEC were cultured in Medium 199 (ref: 22340-020, Gibco) containing 20% Fetal Calf Serum (ref: 10270106, Gibco), 150 μg/mL endothelial cell growth supplements (ref: 02-102, Merck Millipore), 100 μg/mL Heparin sodium grade I-A (ref: H3149, Sigma Aldrich), 100 μM hydrocortisone (ref: H6909, Sigma Aldrich), 10 μg/mL ascorbic acid (vitamin C) (ref: 4544, Sigma Aldrich) and 1/1000 of PSF (penicillin, streptomycin and fungizone) (ref: 4-02F00-H, AMIMED). eEnd2 cells and HEK293T cells were cultured in Dulbecco’s Modified Eagle Medium (DMEM + GlutaMax, ref: 61965-026, Gibco), 10% of decomplemented fetal calf serum (ref: 10270106, Gibco), 1% penicillin/streptomycin (ref: 15140, Gibco) and 1% L-glutamine (ref: 25030-024, Gibco). All cells were cultured under incubation in a humidified environment with 10% CO_2_ at 37°C.

### Method details

#### Reagents, antibodies and primers

We used 96-well plate black clear half area for seeding cells in proliferation assay and ibidi μ-slides VI 0.4 for other immunofluorescence experiments. We used Transwell® (ref. 3470, Costar) of 0.4 μm pore polycarbonate membrane insert in 24-well plate and Dextran-FITC (ref 46948-100MG-F, MW:150'000, Sigma-Aldrich) to perform permeability assay. For pulldown assay with biotinylated peptides, we have used Dynabeads M-280 Streptavidin (11205D, Thermo Fisher Scientific). Biotinylated peptides of human Integrin-β1 were synthesized by the peptide synthesis core facility (PPR2P platform) of the faculty of medicine of the University of Geneva. We used the RNA and protein extraction kit (Ref: 740933.50, Macherey-Nagel) for RNA and protein extraction. We have used the EdU (5-ethynyl-2′-deoxyuridine) and the Alexa fluor 647 picolyl-azide from the staining kit (ref: C10635, Lot: 1705785, Thermo Fisher Scientific or ref. 1300-1, Click chemistry tools) and Hoechst 33342 (ref. 62249, Thermo Fisher Scientific). For immunofluorescence imaging and western blotting we have used the polyclonal rabbit anti-KINDLIN2 (ref. K3269, Sigma-Aldrich), monoclonal rat anti- mouse VE-cadherin (clone 11D4.1, BD Biosciences), Polyclonal goat anti VE-cadherin (C19) (Ref: sc6458, lot: D1614, Santa Cruz Biotec), monoclonal mouse anti-GAPDH (clone: 1E6D9) (Ref: 60004-1-Ig, lot:100021642, Proteintech), horseradish peroxidase conjugated goat anti-rabbit IgG (H + L, min X) (ref:111.035.144, lot:71776, Jackson ImmunoResearch), Horseradish peroxidase conjugated donkey anti-rabbit IgG (ref: sc-2020, lot:K0615, Santa Cruz Biotec), AF555-Goat anti rat IgG (H + L) cross-adsorbed secondary antibody (ref: A21434, lot:2272647, Thermo Fisher Scientific). For molecular biology, pCAG-VSVG was a gift from Arthur Nienhuis & Patrick Salmon (Addgene plasmid # 35616; http://n2t.net/addgene:35616; RRID:Addgene_35616), psPAX2 was a gift from Didier Trono (Addgene plasmid # 12260; http://n2t.net/addgene:12260; RRID:Addgene_12260), pWPT-GFP was a gift from Didier Trono (Addgene plasmid # 12255; http://n2t.net/addgene:12255; RRID:Addgene_12255), pCMV6-AC-GFP empty vector (ref. PS100010) was purchased from Origene and the pcDNA3-SAPMAR-ITGB1AK107-GFP (wt or K794Q mutant) were previously produced in house and reported by Vega et *al.*^25^

For quantitative polymerase chain reactions (qPCR) in order to quantify gene expression, we have used primers listed in the Table S3.

#### Cell culture

HUVEC were cultured in Medium 199 (ref: 22340-020, Gibco) containing 20% Fetal Calf Serum (ref: 10270106, Gibco), 150 μg/mL endothelial cell growth supplements (ref: 02-102, Merck Millipore), 100 μg/mL Heparin sodium grade I-A (ref: H3149, Sigma Aldrich), 100 μM hydrocortisone (ref: H6909, Sigma Aldrich), 10 μg/mL ascorbic acid (vitamin C) (ref: 4544, Sigma Aldrich) and 1/1000 of PSF (penicillin, streptomycin and fungizone) (ref: 4-02F00-H, AMIMED). eEnd2 cells and HEK293T cells were cultured in Dulbecco’s Modified Eagle Medium (DMEM + GlutaMax, ref: 61965-026, Gibco), 10% of decomplemented fetal calf serum (ref: 10270106, Gibco), 1% penicillin/streptomycin (ref: 15140, Gibco) and 1% L-glutamine (ref: 25030-024, Gibco). All cells were cultured under incubation in a humidified environment with 10% CO_2_ at 37°C.

#### Gene expression analysis by RNA sequencing

For expression of human Integrin-β1, we used the previously^25^ generated pCDNA3 plasmid with the CMV promoter replaced by a fragment of matrix attachment region of chicken lysozyme (MAR) and a small fragment of human actin beta promoter (SAP), and encoding for human Integrin-β1A containing an extracellular tagging after the K107 with the green fluorescence protein.^63^ Mouse embryonic endothelial cell line eEnd2 was transfected with this pcDNA3-SAPMAR-ITGB1AK107-GFP encoding for human Integrin-β1A (wildtype or K794Q mutant)-eGFP chimera, by using Neon nucleofector (Thermo Fisher Scientific) according to the manufacturer recommendation. GFP positive cells were sorted by fluorescence-activated cell sorting (FACS) and sub-cultured until use. Sorted cells were cultured in 10-cm dishes to confluence and the total mRNA were extracted with the RNA and protein extraction kit. Total RNA of three different cultures of each sorted cells were sequenced, aligned and the differential expression of genes analyzed at the iGE3 Genomics Platform (University of Geneva). Sequences were aligned to mouse genome as reference using the Spliced Transcripts Alignment to a Reference (STAR) software.^64^ Alignment to human genome was also performed in order to check the level of *ITGB1A-GFP*. To analyze the differential expression of genes, the data were normalized by using edgeR package of R software and the differences were analyzed by the general linear model (GLM), negative binomial distribution and a quasi-likelihood F-test. The difference in gene expression between wt and K794Q cells was considered when the threshold difference ≥ two-fold with *p*-value ≤0.05. Differentially expressed genes were hierarchically clustered and the corresponding heatmap was represented. Further analysis was performed to determine the gene ontology (GO) and the pathways in which they are involved according to the Kyoto Encyclopedia of Genes and Genomes (KEGG) database.

#### Generation of lentiviral vectors

We first transferred the full cDNA sequence of human KINDLIN2 with the following primers (fwd: CTCGCGAGCCCGCATGGCTCTGGACGGG and rev: GTACCTCGAGCACCCAACCACTGGTAAG) from a N-terminal tagging pCMV-CFP-KIND2 plasmid into a pCMV6-AC-GFP empty vector (ref. PS100010, Origene) with a C-terminal tagging turboGFP by using PrimeSTAR Max DNA polymerase (ref R045A, TAKARA). The vector and PCR products were digested by the restriction enzymes NotI and XhoI according to the manufacturer recommendation. Digestion products were assembled with T4 ligase. The ligated plasmid was used to transform competent DH5-alpha *E. coli* bacteria and positive clones were selected on agarose plates for their resistance to ampicillin. The C-terminally tagged KINDLIN2-turboGFP was then transferred to a lentiviral vector pWPT-GFP (Addgene plasmid # 12255) under the promoter of EF1alpha for expression in primary human cells. Therefore KINDLIN2-turboGFP sequence was amplified by using the following primers (fwd: AACACAGGTGTCGTGACGCGGATCCGCGGCCGCCATGGCTCTG and rev: TTATCGGAATTCCCTCGAGGTCGACGTTTAAACTCTTTCTTCACCGGCATCTGCATCC) with the PrimeSTAR Max DNA polymerase. The new lentiviral plasmid pWPT-EF1a-KIND2-turboGFP was assembled by using Gibson assembly with the HIFI DNA Assembly Master Mix (ref: E2621, New England Biolabs Inc.). Similarly, for the generation of the lentiviral vectors allowing the expression of wildtype and K794Q mutant of human Integrin-β1 tagged with GFP on the extracellular portion, we used the pcDNA3-SAPMAR-ITGB1A-GFP^27^ vectors encoding for either wt or K794Q mutant and amplified the sequence encoding for the two Integrin-β1-GFP chimeras with the following primers (fwd: AACACAGGTGTCGTGACGCGGATCCACCATGAATTTACAACCAATTTTC; rev: TTATCGGAATTCCCTCGAGGTCGACTCTAGACTCATTTTCCCTC). The lentiviral vectors pWPT-EF1a-B1A-GFP (wt or K794Q) were generated by using Gibson assembly with the HIFI DNA Assembly Master Mix. For the generation of the lentiviral vectors allowing the expression of human VE-CADHERIN with a C-terminal tagging tdTomato on the cytoplasmic tail, we used the tdTomato-VE-cadherin-N-10 vector, a gift from Michael Davidson (Addgene plasmid # 58142; http://n2t.net/addgene:58142; RRID:Addgene_58142)^65^ and amplified the sequence encoding for the human VE-CADHERIN-tdTomato chimera with the following primers (fwd: GTGTCGTGACGCGGATCCGCGCTAGCCGCCACCATGCA; rev: TTCCCTCGAGGTCGACGTTTTCTAGAGTCGCGGCCGCTTAC). The lentiviral vectors pWPT-EF1a-VE-CADHERIN-tdTomato were generated by using Gibson assembly with the HIFI DNA Assembly Master Mix. All the assembled plasmids were used to transform competent DH5-alpha *E. coli* bacteria for plasmid amplification. The new plasmids were sequenced, amplified and purified before use for lentivirus production.

#### Lentivirus production and HUVEC infection

HEK293T cells in 10-cm dishes were cultured to about 40–70% confluence in Dulbecco’s Modified Eagle Medium (DMEM + GlutaMax, ref: 61965-026, Gibco) containing 10% of decomplemented fetal calf serum (ref: 10270106, Gibco), 1% penicillin/streptomycin (Ref: 15140, Gibco) and 1% L-glutamine (ref: 25030-024, Gibco). HEK293T cells were transfected with the three plasmid pWPT-EF1a-KIND2-turboGFP or pWPT-EF1a-VE-CADHERIN-tdTomato or the pWPT-EF1a-B1A-GFP vectors (wt or K794Q), pCAG-VSVG and psPAX2 with Lipofectamine2000 (Ref 11668027, Invitrogen) according to the manufacturer instructions. The medium of HEK293T cells was changed with OptiMEM (ref: 31985-047, Gibco) before transfection with the three plasmids at the following concentrations: 13 μg of the protein of interest encoding vectors (pWPT-EF1a-KIND2-turboGFP, pWPT-EF1a-VE-CADHERIN-tdTomato, pWPT-EF1a-B1Awt-GFP or pWPT-EF1a-B1AK794Q-GFP), 2.1 μg of pCAG-VSVG and 6.3 μg of psPAX2 per transfection. After 5 days of culture, the supernatants of the cells were collected and centrifuged at 1200 rpm for 10 min in order to discard eventual floating cells. Lentivirus-containing supernatants after centrifugation were harvested leaving a volume at the bottom of the tube to avoid cell pellets. Medium containing lentivirus was aliquoted and stored at −80°C until use for cell infection.

HUVEC seeded on plate pre-coated with the mix of 0.1 mg/mL collagen G (Ref:L7213, Biochrom GmbH)/0.2% gelatin (G1393, Sigma-Aldrich) diluted in phosphate buffered saline (PBS) were cultured in HUVEC culture medium made with Medium 199 (ref: 22340-020, Gibco) containing 20% FCS, 150 μg/mL endothelial cell growth supplements (ref: 02-102, Merck Millipore), 100 μg/mL Heparin sodium grade I-A (ref: H3149, Sigma Aldrich), 100 μM hydrocortisone (ref: H6909, Sigma Aldrich), 10 μg/mL ascorbic acid (vitamin C) (ref: 4544, Sigma Aldrich) and 1/1000 penicillin/streptomycin/fungizone (ref: 4-02F00-H, AMIMED). At 50–90% confluence in 10-cm dish, HUVEC were infected with 500 μL conditioned medium containing a given lentivirus supplemented with 10 μg/mL (final) polybrene (ref: sc-134220, Santa Cruz biotech) and incubated at 37°C with 5% CO_2_ overnight before changing the medium. Infected HUVEC were cultured for at least 4–5 days before sorting positive cells by FACS, sub-culturing or use for experimentation.

#### Quantification of endothelial cell proliferation

Cell proliferation was estimated by quantifying the incorporation of EdU to the DNA of dividing cells. To analyze the proliferation of mouse embryonic endothelial cells eEnd2 expressing either the wildtype or the K794Q mutant of Integrin-β1A-GFP, cells were seeded at low density in wells of 96-wells plate coated or not with 10 μg/mL of Fibronectin (Ref: FC010, EMD Millipore), Laminin-1 (a gift from Jürgen Engel, Biozentrum Basel), Vitronectin^66^ (a gift from Vesa Hytönen, Tampere) or mix of collagenG/gelatin and cultured for 24 h in complete DMEM (DMEM + GlutaMax, ref: 61965-026, Gibco), 10% of decomplemented fetal calf serum (ref: 10270106, Gibco), 1% penicillin/streptomycin (ref: 15140, Gibco) and 1% L-glutamine (ref: 25030-024, Gibco). Cell medium was replaced by simple DMEM for starvation overnight and then cultured in complete DMEM containing 10 μM EdU (ref: C10635, Lot: 1705785, Thermo Fisher Scientific) for 6 h. Cells were washed once with PBS with magnesium and calcium before fixation with with 4% paraformaldehyde (PFA) for 10 min. Cells were permeabilized with 0.2% Triton-X-100 for 10 min and washed with PBS-Ca+Mg+ before proceeding with Click reaction in order to allow detection of incorporated EdU with AF647-pycolyl azide from the kit (ref. C10635, Lot: 1705785, Thermo Fisher Scientific) following the manufacturer instructions. Total nuclei were stained with 5 μM Hoechst 33342 for 5 min at room temperature. Cells were imaged with ImageXpress (Molecular Device). Filters of GFP, cyanine 5 and DAPI were used to image respectively human Integrin-β1A-GFP, AF647-EdU positive nuclei and total nuclei. The total surface of each well was covered by imaging 4X4 sites at 10X magnification that were finally stitched together to have a complete picture of each well in the plate. Images were analyzed by counting EdU positive and total nuclei by using nuclei count module of MetaXpress (Molecular Device). Data were exported as “.csv” file to calculate the percentage of EdU positive nuclei as an index of cell proliferation. Data analysis and graph presentations were performed by using GraphPad Prism 9 (GraphPad Software, LLC). Representative images of single or combined channels were processed with ImageJ software (v1.53q) and figures were mounted with Inkscape software (v1.3.2).

#### Analysis cell contact inhibition of proliferation

To evaluate the impact of cell density (the contact inhibition of cell proliferation), we first analyzed the proliferation of mouse eEnd2 cells expressing either the wildtype or the K794Q mutant of human Integrin-β1A-GFP cultured to confluence. The contact inhibition induced by cell confluence was analyzed by evaluating EdU incorporation to the DNA of confluent cells as described above. To specifically make a gradient of cell density in order to evaluate the efficiency of contact inhibition, air bubbles were made in one side of the well of 96-well plate with clear bottom and half area (ref: 675090, Greiner) by using P20 Pipetman (GILSON) charged with an empty tip. About 2–5 μL of air bubbles were injected to one side of the bottom of the well already containing a volume (>50 μL) of complete DMEM. Cells were delicately added at high density (∼5x10^3^ cells/well) to the well and incubated at 37°C with 5% CO_2_ overnight to allow cell spreading and contact formation. Air bubble explosion generated wounds that were partially invaded by migrating cells in direct contact with the wounds. Cells were then cultured for 6 h in complete DMEM containing 10 μM EdU and fixed with 4% PFA for 10 min. EdU detection and evaluation of cell proliferation was performed as described above.

#### Immuno-staining of endothelial cell junctions and adhesions

To analyze the cell-cell junctions and adhesions by immunofluorescence, 3x10^4^ to 10^5^ cells (eEnd2 cells or HUVEC) constitutively expressing either human β1-WT-GFP or β1-K794Q-GFP were seeded in chambers of ibidi μ-slides VI 0.4 coated or not with 10 μg/mL Laminin-1 and cultured for 3 to 5 days. Cells were then washed with PBS (Calcium+ Magnesium+), fixed with 4% PFA for 10 min and permeabilized with 0.2% Triton-X-100 for 15 min. Cells junctions were stained with the rat anti-mouse VE-cadherin (clone 11D4.1, BD Bioscience) at 1/200 dilution for 1 h followed by the AF555-conjugated goat anti rat IgG (ref: A21434, Invitrogen) at 1/400 dilution for 1 h. Sites of cell adhesion to ECM were staining with the rat anti-activated (mouse and human) Integrin-β1 (clone 9EG7) at 1/1000 for 1 h followed by the secondary antibodies (either AF555-donkey anti rat or AF650-donkey anti rat (ref: SA5-10029, Thermo Fisher Scientific)) at 1/400 dilution for 1 h at room temperature. The nuclei were stained with Hoechst 33342 (ref. 62249, Thermo Fisher Scientific) at 1/2000 dilution for 5 min. Cells were washed with PBS (Ca+Mg+) and the pictures were taken with the inverted Nikon A1r confocal microscope piloted with the NIS Element AR V4.30.02 64-bit software.

For KINDLIN2 recruitment to adhesions sites, cells were fixed with 1% PFA for 30 min and permeabilized with ice-cold methanol for 5 min. Cells were washed with gradual concentration (90%–0%) of methanol diluted in PBS. The permeabilization was completed with 0.2% Triton-X-100 for 5 min at 4°C. KINDLIN2 was stained with the rabbit anti-Kindlin2 (K3269, Sigma-Aldrich) at 1/1000. The adhesions were detected with the rat anti-Integrin β1 (9EG7) at 1/500 under this condition. Cells were incubated with the primary antibodies for 1 h followed by the secondary antibodies (AF633-goat anti rat (ref: A21094, Thermo Fisher Scientific) and AF555-donkey anti rabbit (ref: A31572, Invitrogen) both at 1/500 for 1 h. The nuclei were stained with Hoechst 33342 at 1/2000 dilution for 5 min. Cells were washed with PBS (Ca+Mg+) and the pictures were taken with the inverted Nikon A1r confocal microscope piloted with the NIS Element AR V4.30.02 64-bit software. Pictures were mounted with ImageJ and combined to figures with Inkscape software.

To investigate the influence of RhoA-induced contractility on the adhesion remodeling, cells were starved in medium without serum for 15 min and then incubated with medium containing or not 5 U/ml Thrombin (ref: T7009, Sigma-Aldrich) for 15 min at 37°C. The cells were then fixed, permeabilized and stained for the specific antibodies as described above.

To determine the cellular localization of KINDLIN2, HUVEC infected with lentivirus to express KINDLIN2-turboGFP were seeded in chambers of Ibidi μ-slides VI 0.4 and cultured for few days. Live cells were directly imaged with the Nikon A1r confocal microscope piloted with the NIS Element AR to capture stack images of GFP fluorescence along the z-axis. Pictures were mounted with ImageJ and combined to figures with Inkscape software.

#### Analysis of KINDLIN2-dependent gene expression by qPCR

We used KINDLIN2 overexpression to stimulate the signaling pathways-dependent on Integrin-β1/KINDLIN2 induced adhesions in HUVEC. HUVEC were infected with lentivirus carrying the pWPT-EF1a-KIND2-turboGFP as described above. The FACS sorting of populations with different levels of GFP positive HUVEC and their sub-culturing allowed to obtain three cell populations. The three populations of HUVEC expressing different levels of KINDLIN2 (the normal: expressing the endogenous KINDLIN2 level, mild: expressing the endogenous KINDLIN2 and a low level of KINDLIN2-turboGFP, and the high: expressing the endogenous KINDLIN2 and a high level of KINDLIN2-turboGFP) were used in different experiments. To analyze the transcription levels of genes by quantitative polymerase chain reaction (qPCR), HUVEC expressing the three levels of KINDLIN2 were stimulated or not with 25 ng/mL tumor necrosis factor (TNFalpha) in HUVEC culture medium as described above for 6 Hrs. The cells were then lysed and the total RNA as well as proteins extracted by using the RNA and protein extraction kit (ref: 740933.50, Macherey-Nagel). The transcript levels of genes were analyzed by qPCR at the genomic facility of the faculty of medicine (University of Geneva). The primers used for qPCR are listed in the Table S3. The expression of genes was normalized with the levels of transcripts of *B2M* and *EEF1* encoding respectively for β-2 microglobulin and the eukaryote elongation factor 1. The impacts of KINDLIN2 overexpression and the TNFalpha-treatment on gene expression was analyzed by performing principal component analysis (PCA) with ClustVis web tool.^67^ The difference between samples was considered significant when *p*-value< 0.05 in two-way ANOVA test.

#### Analysis of integrin-β1-dependent signaling pathways

To analyze the impact of Integrin-β1 acetylation on the mechanosensible Hippo-Yap signaling pathway, the expression levels of Yap targeting genes were analyzed from the microarray RNAseq data of the mouse embryonic endothelial cells eEnd2 expressing the wildtype or K794Q mutant of human B1A-GFP. The set of Yap-targeting genes that was previously reported by Wang et al.^32^ include *Cyr61, Ctgf, Amotl2, Ankrd1, Igfbo3, Lats1, Crim1, Gadd45a, Tgfb2, Ptpn14, Axl, Dock5, Asap1, Rbls3, Myof, Arhgef17 and Ccdc80*.

To analyze the impact of Integrin-β1 acetylation on the mitogen-activated protein kinase (MAPK) signaling pathways, HUVEC were infected with lentiviruses in order to constitutively express human β1-wt-GFP or β1-K794Q-GFP as described above. The infected cells were pre-stimulated with 1 mM Na3VO4, 1 mM NaF and either dimethylsulfoxide (DMSO) or the mix of 5 μM Tyrphostin 47 (Ref: 658405, Calbiochem)/5 μM LY294002 (Ref: 19-142, Sigma-Aldrich) for 15 min. Then the cells were stimulated or not with 50 ng/mL vascular endothelial growth factor (VEGF) in presence of the mix of the inhibitors or DMSO for 15 more minutes. Cells were lysed in non-denaturing lysis buffer supplemented with PhosSTOP (ref: 4906845001, ROCHE) and a mini complete tablet of protease inhibitors (ref: 04693159001, ROCHE). About 15 μg of protein from each lysate were resolved on polyacrylamide gel, transferred on nitrocellulose membrane. The loading and blotting of equal protein quantity was checked by the membrane incubation with Ponceau S Solution (Ref: 33427, SERVA) for 5 min and washed with distilled water before taking pictures and the membranes were sequentially blocked and immunoblotted with antibodies against phospho-p42/p44 ERK (ref: 9106S, lot: 27, Cell Signaling Technology), phospho-SRC(Y527) (ref: 2105S, lot: 9, Cell Signaling technology) mouse anti-Tubulin-alpha (ref: T5168, Sigma Aldrich), mouse anti-Vinculin (ref: V9131, Sigma Aldrich) and/or VE-cadherin (ref: sc6458, lot: D1614, Santa Cruz Biotec). Membranes were stained with the specific secondary antibodies conjugated to horseradish peroxidase and bands were revealed by using the WesternBright Sirius HRP substrate (ref. K-12043-D20, Advansta). The pictures of the membranes were photographed with the Fusion FX imager (Vilber), analyzed with ImageJ and combined to a figure with the illustration software Inkscape.

In a similar fashion, to analyze the impact of different cellular amounts of KINDLIN2 on MAPK and PI3K signaling pathways, HUVEC expressing different levels of KINDLIN2 (Norm: endogenous KINDLIN2 only, Mild: endogenous KINDLIN2 and low level of KINDLIN2-turboGFP or High: endogenous KINDLIN2 and high level of KINDLIN2-turboGFP) were stimulated or not with 25 ng/mL TNFalpha (ref: 300-01A, PeproTech) TNFalpha for 20 min and lysed with non-denaturing lysis buffer containing PhosSTOP (ref. 4906845001, ROCHE) and complete tablet of protease inhibitor (ref: 04693159001, ROCHE). The protein extracts were analyzed by western blotting after electrophoresis in polyacrylamide gel as described above. The membranes were sequentially stained with the following primary antibodies: rabbit anti-Kindlin2 (ref. K3269, Sigma-Aldrich), rabbit anti-phospho p42/p44 ERK (ref: 9106S, lot: 27, Cell Signaling Technology), rabbit anti-phospho AKT (S473) (ref: 9271S, lot: 14, Cell Signaling Technology), mouse anti-Tubulin-alpha (ref: T5168, Sigma Aldrich), mouse anti-Vinculin (ref: V9131, Sigma-Aldrich) and/or goat anti-VE-cadherin (ref: sc6458, lot: D1614, Santa Cruz Biotec). The specific secondary antibodies conjugated to the Horseradish peroxidase (see the secondary antibody list above) as well as the WesternBright Sirius HRP substrate (ref: K-12043-D20, Advansta) to reveal the bands. The membranes and the luminescence were photographed and the pictures were analyzed with ImageJ and combined to figures with the Inkscape software.

#### Endothelial permeability assay

To analyze the impacts of KINDLIN2 overexpression on cell permeability induced by TNFalpha-treatment, HUVEC (10^4^ cells per insert) expressing different levels of KINDLIN2 namely (Norm: endogenous KINDLIN2 only, Mild: endogenous KINDLIN2 and low level of KINDLIN2-turboGFP or High: endogenous KINDLIN2 and high level of KINDLIN2-turboGFP) were seeded on the insert of Transwell (ref. 3470, Costar) and cultured to confluence. The day of the experiment, the monolayer was stimulated or not with 100 ng/mL TNFalpha for 6 Hrs. The cells were cultured in 100 μL of medium in the upper compartment and 500 μL in the bottom compartment. The capacity of the fluorescein-conjugated dextran (Sigma-Aldrich 46948-100MG-F, MW:150'000) to pass through the endothelium was tested by loading 0.5 mg/mL dextran-FITC in the upper compartment of the filter. After 5 min, the amount of leaked dextran was quantified in the medium of the bottom compartment. Therefore, the fluorescence at 488 nm wavelength was measured in the medium by direct reading with the SpectraMax Paradigm microplate reader (Molecular Devices). The data were analyzed and assembled with Microsoft Excel software and analyzed with GraphPad Prism. The difference was considered as significant if *p*-value <0.05 in two-way ANOVA test.

#### Biochemical analysis of KINDLIN2/Integrin-β1 binding

To analyze KINDLIN2 binding to Integrin-β1 and the impact of K794 modification by acetylation, different biotinylated peptides of the cytoplasmic tail of Integrin-β1 carrying modifications of this lysine residue were synthesized at the protein and peptide synthesis facility of the faculty of medicine (University of Geneva). They include biotinylated peptides with non-modified (pep_wt_(K)), acetylated (pep_Ac-K794), the acetylation mimetic mutation (K794Q) and the positive charge-conserved acetylation mimetic mutation (K794R). Scrambled acetylated peptides were used as negative control of the binding assays. The peptides were used to decorate the streptavidin coupled magnetic Dynabeads M-280 (Thermo Fisher) according to the manufacturer instructions. Briefly, 0.5 mg of magnetic beads were washed with PBS containing 0.1% Tween20 (PBST) and incubated with 1 nM peptide in PBS for 30 min under shaking (150 rpm) at 4°C. The supernatant and the beads-peptides complexes were separated by using a in house made magnetic tube holder. The beads decorated with biotinylated peptides were washed with PBST for 1 h under shaking (150 rpm) at 4°C. Once the supernatant was discarded from the tube in the magnetic tube holder, the beads-peptides complexes were re-suspended in 200 μg protein extracts of HUVEC diluted in PBST. Protein binding to the beads-peptides complexes was carried out under shaking (150 rpm) overnight at 4°C. The pulled-down proteins and the interacting beads-peptides were separated from non-bound protein suspension with the magnetic tube holder. The complexes were washed twice with PBST and PBS before re-suspension with 1x Laemmli protein loading buffer. The samples were heated at 95°C for 10 min. Denatured proteins were separated from the streptavidin-beads and the peptides by using the magnetic tube holder. The supernatants containing the pulled-down proteins were loaded on polyacrylamide gel and resolved by electrophoresis. Western blotting of proteins was performed after transfer on nitrocellulose membrane by using antibody against KIND2 (ref. K3269, Sigma-Aldrich). The specific secondary antibodies conjugated to the Horseradish peroxidase and the WesternBright Sirius HRP substrate (ref: K-12043-D20, Advansta) was used to reveal the bands. The membranes and the luminescence were photographed and the pictures were analyzed with ImageJ and combined to figures with Inkscape.

#### Molecular dynamics simulations

Crystal structure 5XQ0 (kindlin2 – residues 570-680, Integrin-β1 – residues 787-797)^47^ was used as a starting structure in MD simulations. All MD simulations were performed with Gromacs ver. 2019.4^68^ using Amber14SB force field^69^ with SPC/E water model in 0.15 M KCl.^70^ The systems were energy minimized and then equilibrated using harmonic position restraints on all heavy atoms of the protein. The temperature and pressure of the system was maintained at 310 K and 1 bar using V-rescale^71^ and Parrinello-Rahman^72^ algorithms. Integration time step of 4 fs was used, enabled by the virtual sites for all hydrogen atoms. Force-field topologies for acetylated lysine was prepared using ACPYPE^73^ and antechamber tool of AmberTools19.^74^ Five independent MD trajectories were generated for each system, where each trajectory was 200 ns long. Structure snapshots at every 250 ps were used for the binding energy analysis using MM-PBSA method with g_mmpbsa tool.^75^ The trajectory time frame for the analysis was 20–200 ns, as first 20 ns for each trajectory was considered as equilibration and was not used for the analysis.

#### Protein production for crystallography

The mouse kindlin2 gene with the PH domain encoding region (137-512) deleted was cloned into a modified pET28a vector. The mouse β1 Integrin-tail (784-798, KSAVTTVVNPQYEGK) with the K794Q mutation was fused to the C-terminal end of Kindlin2 following a linker LVPRGSGSGSGS. The sequence-confirmed plasmids were transformed into E. coli BL21(DE3) cells (Thermo Fisher Scientific). The bacteria were cultured in Luria broth (LB) medium containing 25 mg/mL kanamycin at 37°C until the optical density at 600 nm reached 0.6. Protein expression was induced by supplementing 0.2 mM Isopropyl β-D-1-thiogalactopyranoside (IPTG) at 18°C overnight. Bacteria were prepared by homogenization (Emulsiflex C3) in 50 mM Tris pH 7.5 and 500 mM NaCl. Clarified lysates were applied to a 5 mL HisTrap FF affinity column (GE healthcare) using ÄKTA Purifier (GE Healthcare) and eluted with an imidazole concentration gradient (20–500 mM). Eluted fractions were analyzed by sodium dodecyl sulfate polyacrylamide gel electrophoresis (SDS/PAGE) and Coomassie staining, and appropriate fractions were pooled. The Kindlin2 protein was diluted with a 10 times volume of 50 mM Tris pH 8.5, 2 mM DTT buffer and applied onto a 6 mL Resource Q column (GE Healthcare). Elution was performed by preparing a linear NaCl gradient with 50 mM Tris pH 8.5, 1 M NaCl, 2 mM DTT buffer. Fractions containing the Kindlin-2 protein were concentrated and loaded on a Superdex 200 column (GE Healthcare) in 20 mM Tris pH 8.0, 200 mM NaCl. The peak fractions were concentrated to ∼10 mg/mL, aliquoted, flash frozen in liquid nitrogen, and stored at −80°C for further use.

#### Protein crystallography

The Kindlin2/β1-K794Q Integrin fusion protein was crystallized in 0.1 M Tris pH 8.5, 10% Isopropanol using the hanging-drop vapor diffusion method. Crystals were observed within 1 to 3 days at room temperature. X-ray diffraction data were collected at the NSLS-II AMX beamline. The data were indexed, integrated, and scaled with FastDP. Data collection and refinement statistics are listed in Table 1. The crystal structure was determined by molecular replacement using Phaser. The atomic coordinates and structure factors have been deposited in the Protein Data Bank with accession number 8TEE. The acetylated β1 Integrin-peptide (KSAVTTVVNP-K(Ac)-YEGK) was synthesized by GeneScript. The peptide was mixed with Kindlin2 protein at a molar ratio of 3:1, and then co-crystallized under the same conditions as the Kindlin2/β1-K794Q fusion protein. X-ray diffraction data were collected at the APS 24-ID-E beamline. The data were indexed, integrated, and scaled with XDS. Structure determination procedure was the same as the Kindlin2/β1-K794Q fusion protein. The crystal contained two Kindlin2 molecules in one asymmetric unit, with one acetylated peptide bound to one of the two molecules. The atomic coordinates and structure factors have been deposited in the Protein Data Bank with accession number 8TEC.

**Table 1:** Crystallographic data collection and refinement statistics

Data collection	KINDLIN2: β1-AcK794	KINDLIN2-β1(K794Q) fusion
Light Source	APS 24-IDE	NSLS-II AMX
Wavelength (Å)	0.97918	0.91975
Space group	P1 21 1	P212121
Cell dimensions		
*a*, *b*, *c* (Å)	73.49, 102.09, 76.03	72.20, 97.90, 72.20
α, β, γ (°)	90, 96.81, 90	90, 96.40, 90
Resolution (Å)	102.09–2.04 (2.09–2.04)	29.94–2.49 (2.55–2.49)
*R*merge (%)	6.9 (187.2)	12.4 (62.3)
*I*/σ	12.8 (0.90)	8.60 (1.80)
CC(1/2)	99.9 (39.4)	99.9 (87.5)
Completeness (%)	99.9 (39.4)	97.0 (72.5)
No. reflections	69860(3942)	36657(2159)
Multiplicity	6.8 (6.1)	6.8 (6.2)
Refinement		
Resolution (Å)	75.49–2.04	29.94–2.49
*R*work/*R*free (%)	19.38/23.02	22.56/27.43
No. atoms		
Protein	6725	6559
Peptide	87	170
Water	243	98
Average B factor (Å^2^)		
Protein	74.36	70.38
Peptide	115.81	79.84
R.m.s deviations		
Bond lengths (Å)	0.007	0.0025
Bond angles (°)	0.812	0.583
Ramachandran (%)		
Favored regions	95.59	94.50
Allowed regions	3.95	5.27
Outliers	0.46	0.23
PDB code	8TEC	8TEE

*R*_free_ was calculated using 5% of data and the same sums. Parentheses denoted highest resolution bin.

### Quantification and statistical analysis

The data presentation is specifically described in each of the figure legends according to the experiment. They were presented either as dot plot with median or mean, or single mean value in bar plots as indicated in the figure legend. RNAseq data were presented as volcano plots to show the extend of changes of transcript levels due to the constitutive mimetism of Integrin-β1 acetylation. The heatmap of significantly up- or down-regulated genes of the RNAseq was also shown by using clustering methods to highlight how the expression of these genes may be interconnected or coregulated by acetylation mimetism. The statistical comparisons were performed where indicated in the specific method section or figure legend. In general, comparison between two experimental groups from independent experiments (qPCR or permeability experiments) or different biological samples in RNAseq analysis was done with Student’s t test or Mann–Whitney non-parametric test as indicated in the figure legends. ANOVA test was performed with Holm-Sidak adjustment for multiple comparison. The analysis was performed by using R edgeR software or Graphpad Prism 9. An effect was considered significant when *p*-value <0.05.

## Supplemental information

Documnet S1. Figures S1–S12 and Tables S1–S3
