# Supplementary material for: Acetyl-NPKY of integrin-β1 binds KINDLIN2 to control endothelial cell proliferation and junctional integrity
Source: iScience. 2024 May 28;27(6):110129. doi: 10.1016/j.isci.2024.110129 (PMC11187247; doi:10.1016/j.isci.2024.110129)
Supplement: Documnet S1. Figures S1–S12 and Tables S1–S3 [file mmc1.pdf]

iScience, Volume *27*

Supplemental information

Acetyl-NPKY of integrin-β1 binds KINDLIN2 to control endothelial cell proliferation and junctional integrity

Adama Sidibé, Vasyl V. Mykuliak, Pingfeng Zhang, Vesa P. Hytönen, Jinhua Wu, and Bernhard Wehrle-Haller

Supplemental information

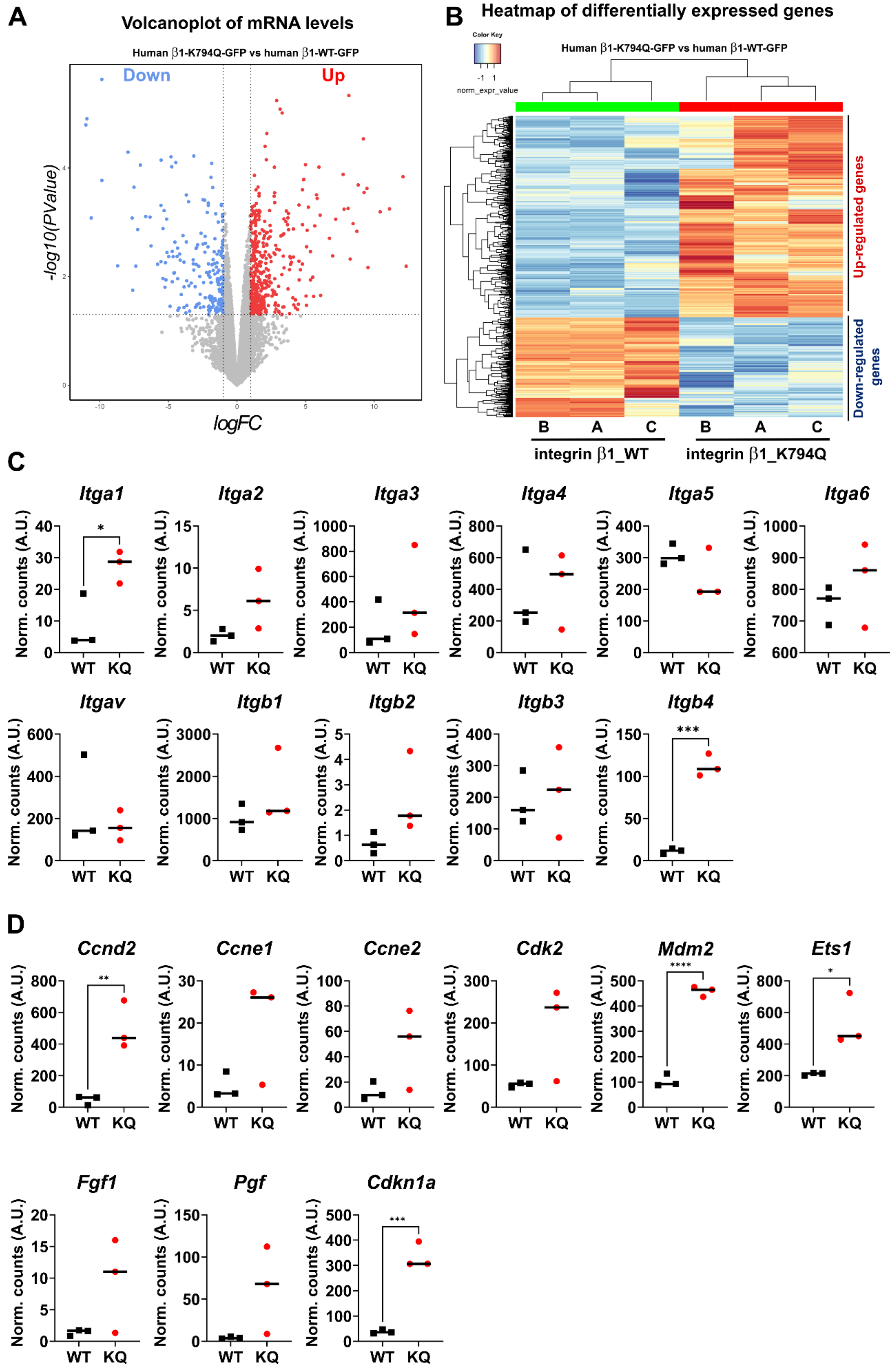

<u>Figure S1</u> : Modification of gene expression by the presence of the human, externally GFP-tagged Integrin-β1 acetylation mimetic K794Q mutant in mouse embryonic endothelial eEnd2 cells: results of a second RNA sequencing experiment (RNAseq II). To support the results presented in the main figures, a second total RNA sequencing of the samples was performed, which showed similar results of differentially expressed genes that were consistent with the first RNAseq of the same samples. A. Volcano plot of all detected genes that emphasizes significantly changed mRNA levels in blue for downregulated or red for upregulated genes, respectively. The thresholds were: fold change FC ≥ 2 and p-value ≤ 0.05. B. Hierarchical heatmap of the differentially expressed genes between mouse embryonic endothelial cells (eEnd2) expressing the human Integrin–β1-GFP (WT vs K794Q)[1]. About 596 genes were found to be differentially expressed (downregulated: 198; upregulated: 398) between β1-wt-GFP- versus β1-K794Q-GFP-expressing eEnd2 cells. This was consistent with the first RNAseq that showed 573 genes differentially expressed (downregulated: 366 and upregulated: 207). About 448 genes were found to be common differentially expressed genes in the two experiments. C. Impact of Integrin-β1 acetylation mimetism on the expression of Integrins. Consistent with the first RNAseq experiment, genes encoding for Integrin-α1 (*Itga1*), α2 (*Itga2*) and β4 (*Itgb4*) were increased by the expression of the acetylation mimetic K794Q mutant of Integrin-β1-GFP, although some changes did not reach significance in the second RNAseq. For example, transcripts of Itga9 were not detectable in the second RNAseq, which was in contrast to the first RNAseq experiment, where it was slightly increased in response to the presence of the acetylation mimetic β1-K794Q mutant. D. Examples of genes involved in cell proliferation that were affected by β1-K794Q-GFP expression. Mainly transcripts of cell cycle regulators such as Ccnd2, Ccne1 and Cdk2, as well as growth factors such as *Fgf1* and *Pgf* were induced by the expression of β1 K794Q-GFP in both RNAseq experiments. Each dot represents a biological sample, and the median is shown as a line. The significance is highlighted with stars when p-value ≤ 0.05 in unpaired t-test. This supplementary figure is related to and supports data of Figure 1 and Figure 2.

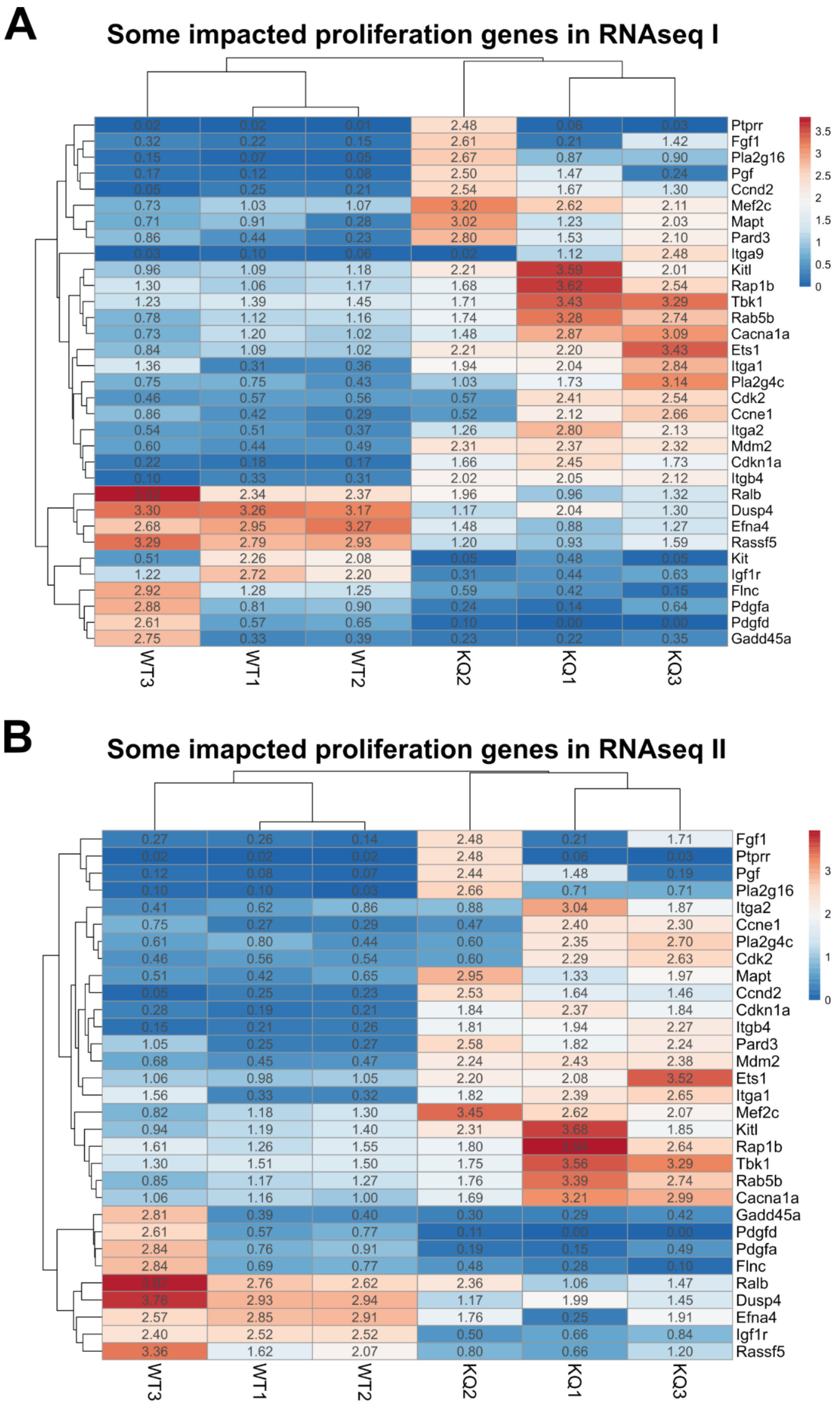
A
Some impacted proliferation genes in RNAseq I
Ptprr
Fgf1
Pla2g16
Pgf
Ccnd2
Mef2c
Mapt
Pard3
Itga9
Kitl
Rap1b
Tbk1
Rab5b
Cacna1a
Ets1
Itga1
Pla2g4c
Cdk2
Ccne1
Itga2
Mdm2
Cdkn1a
Itgb4
Ralb
Dusp4
Efna4
Rassf5
Kit
Igf1r
Flnc
Pdgfa
Pdgfd
Gadd45a
WT3
WT1
WT2
KQ2
KQ1
KQ3
3.5
3
2.5
2
1.5
1
0.5
0
B
Some imapcted proliferation genes in RNAseq II
Fgf1
Ptprr
Pgf
Pla2g16
Itga2
Ccne1
Pla2g4c
Cdk2
Mapt
Ccnd2
Cdkn1a
Itgb4
Pard3
Mdm2
Ets1
Itga1
Mef2c
Kitl
Rap1b
Tbk1
Rab5b
Cacna1a
Gadd45a
Pdgfd
Pdgfa
Flnc
Ralb
Dusp4
Efna4
Igf1r
Rassf5
WT3
WT1
WT2
KQ2
KQ1
KQ3
3
2
1
0

<u>Figure S2</u> : Heatmaps of a subgroup of genes involved in the process of cell proliferation, which were differentially regulated between mouse eEnd2 cells expressing either the WT or the K794Q versions of human Integrin-β1-GFP. The hierarchical heatmaps of the two RNAseq (A: RNAseq I and B: RNAseq II), which were performed on the same samples are shown. The expression of many genes involved in the regulation of cell division check points such cyclins (*Ccnd2* or *Ccne1*) and *Mdm2*, the gene regulator of the tumor suppressor p53, were upregulated in eEnd2 cells expressing the acetylation mimetic K794Q, when compared to cells transfected with its wildtype counterpart. This supplementary data is linked to Figure 2.

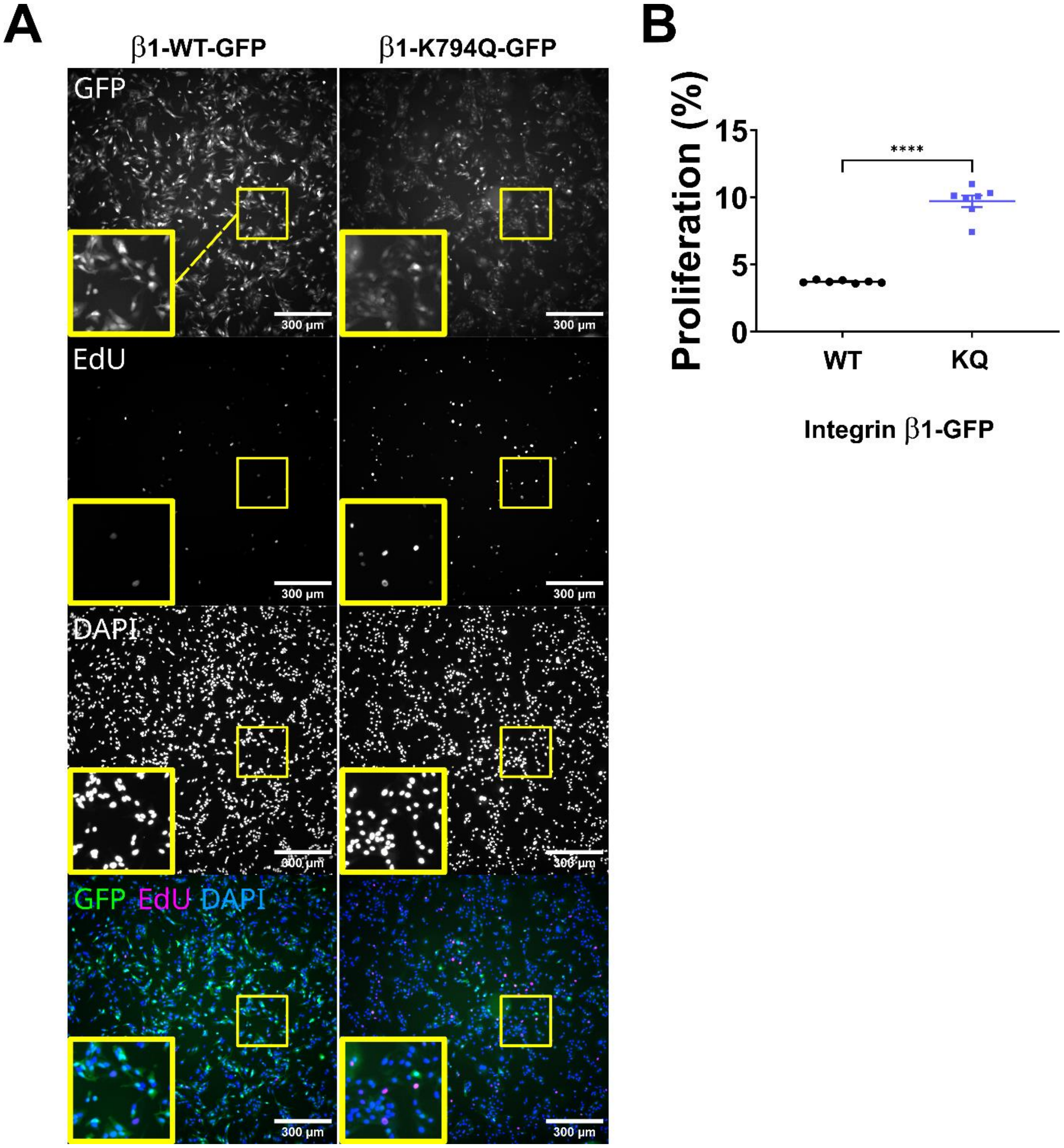

Figure S3: Integrin-β1 acetylation enhances the proliferation of sub-confluent mouse embryonic endothelial eEnd2 cells. A. Representative pictures of the proliferation test of mouse eEnd2 cells expressing either the wildtype or the acetylation mimetic mutant K794Q of human Integrin-β1-GFP. Single channels in grayscale and the merges in colours are presented. The proliferation of starved cells was monitored by the incorporation of EdU into the DNA of dividing cells. GFP (green in merge) shows expression of the human Integrin-β1 version, EdU (magenta) indicates proliferated cells and DAPI (blue) shows staining of the nuclei. Scalebar = 300 µm. The zoom of the squared area is shown in the bottom corner of each picture to better illustrate the difference in proliferating cells between β1-WT or β1-K794Q expressing eEnd2 cells. Zoomed area of the pictures shows that EdU colocalized with a subset of DAPI-stained nuclei. Increased numbers of EdU positive nuclei are visible in mouse eEnd2 cells expressing the acetylation mimetic mutant K794Q. B. Quantification of the proliferation of sub-confluent mouse eEnd2 cells expressing the two versions of human Integrin-β1-GFP (WT or K794Q). Sub-confluent cells were starved overnight and then cultured in the presence of serum-

containing medium complemented with EdU. The expression of the acetylation mimetic K794Q mutant of Integrin-β1-GFP fostered the proliferation of eEnd2 cells when compared to the wildtype counterpart. Each dot represents the percentage of EdU positive cells in a well. The difference was considered significant when p-value ≤ 0.05 in unpaired t-test. The shown result is representative of at least three independent experiments. This figure is related to and supports Figure 2.

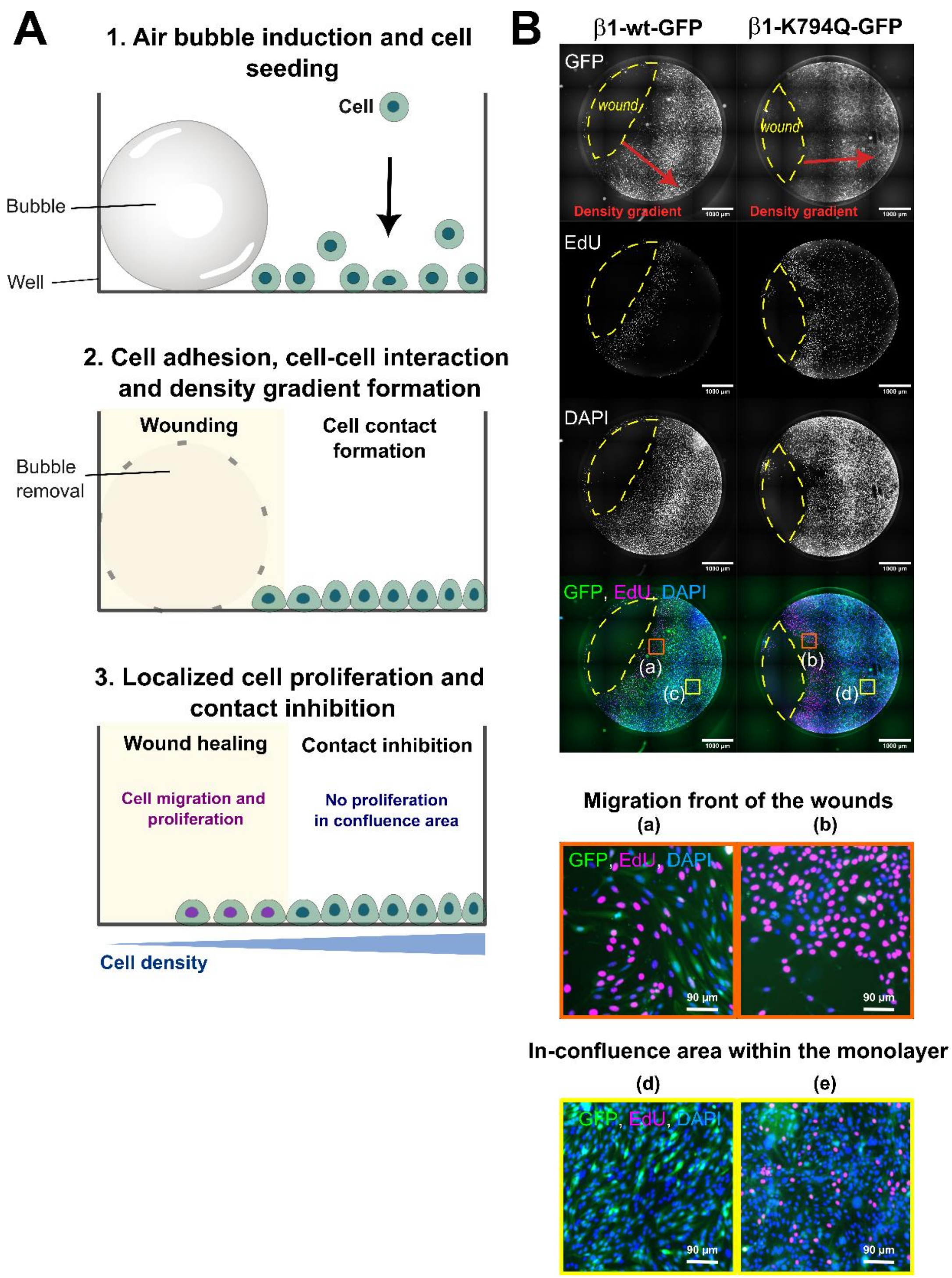

Figure S4: Air bubble-induced wound healing: A model for studying contact inhibition of endothelial cell proliferation. A. Illustration describing the method of studying endothelial cell contact inhibition of proliferation by inducing a one-sided wound with an air-bubble. An air-bubble is made at the bottom of the well and followed by seeding endothelial cells at high density (1). The seeded cells accumulate outside of the air-bubble and adhere to the bottom of the well (2). After a defined time in culture, the air-bubble is removed and endothelial cells will migrate and proliferate at the wound edge (3). The migration process creates a gradient of cell density. By investigating EdU incorporation into proliferating cells, the contact inhibition capacity of the cells can be appreciated inside the monolayer

far from the wound front. B. Example of contact inhibition analysis of eEnd2 cells expressing different forms of human Integrin-β1-GFP (WT or K794Q). Shown are the pictures of the entire wells of air-bubble-induced wound healing made by stitching images acquired in three channels. Single channel pictures are shown in grayscale and their merges are presented in colours as indicated. The wound is delineated with yellow dashed lines and red arrows indicate the gradients of cell density in the wells. In each condition, a zoomed in area of the cell migration front bordering the wound is shown (red square rectangle for a and b). Most of the cells at the migration front went through at least one cell division for all conditions. The in-confluence area within the monolayer framed by yellow rectangles are also zoomed and shown in picture c and d. An efficient contact inhibition can be noticed for eEnd2 cells expressing β1-WT-GFP as no EdU incorporation took place in the in-confluence area (d) and cells positive for EdU were exclusively found in the invading front of the monolayer (a). Please note that for eEnd2 cells expressing the acetylation mimetic mutant K794Q of Integrin-β1-GFP, the incorporation of EdU was not restricted to the cell-migration front, since the in-confluence area showed also several positive cells demonstrating a defect in contact-mediated inhibition of cell proliferation. Scalebar of pictures showing entire wells = 1000 µm and scalebar of the zoomed areas =~90 µm. This data is related to and supports figure 3.

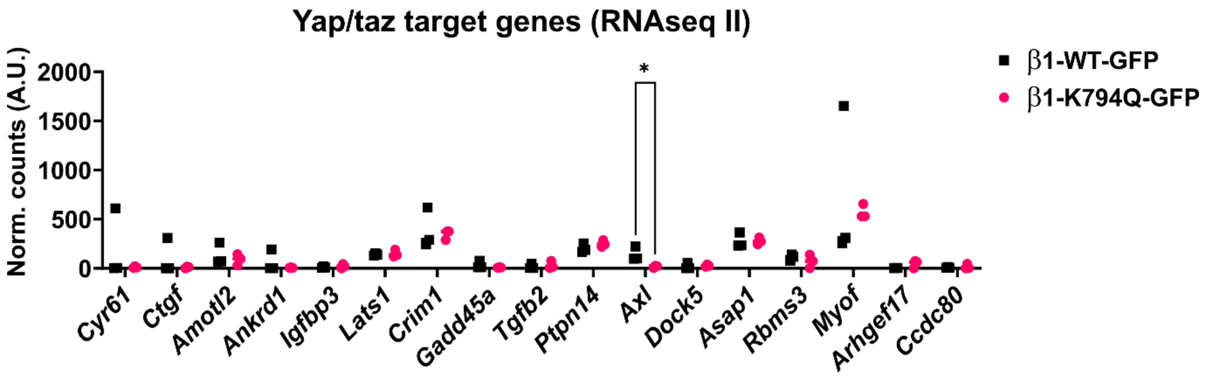

<u>Figure S5</u>: Influence of Integrin-β1 acetylation on the Hippo-Yap signalling pathway in eEnd2 cells: The expression levels of some previously reported genes that are recognized targets[2] of the transcription factors Yap/Taz in eEnd2 cells are shown in wildtype versus acetylation mimetic K794Q transfected human Integrin-β1 (RNAseq II). The same expression patterns were found between RNAseq I and RNAseq II for these Yap/Taz-targeted genes. The transcript levels of most of these genes were not changed between the conditions, suggesting a lack of impact of Integrin-β1 acetylation on the Yap/Taz signalling pathway. Each dot represents a biological sample. The significance is highlighted with stars when p-value ≤ 0.05 in unpaired t-test. This supplementary figure is related to and supports data of Figure 4.

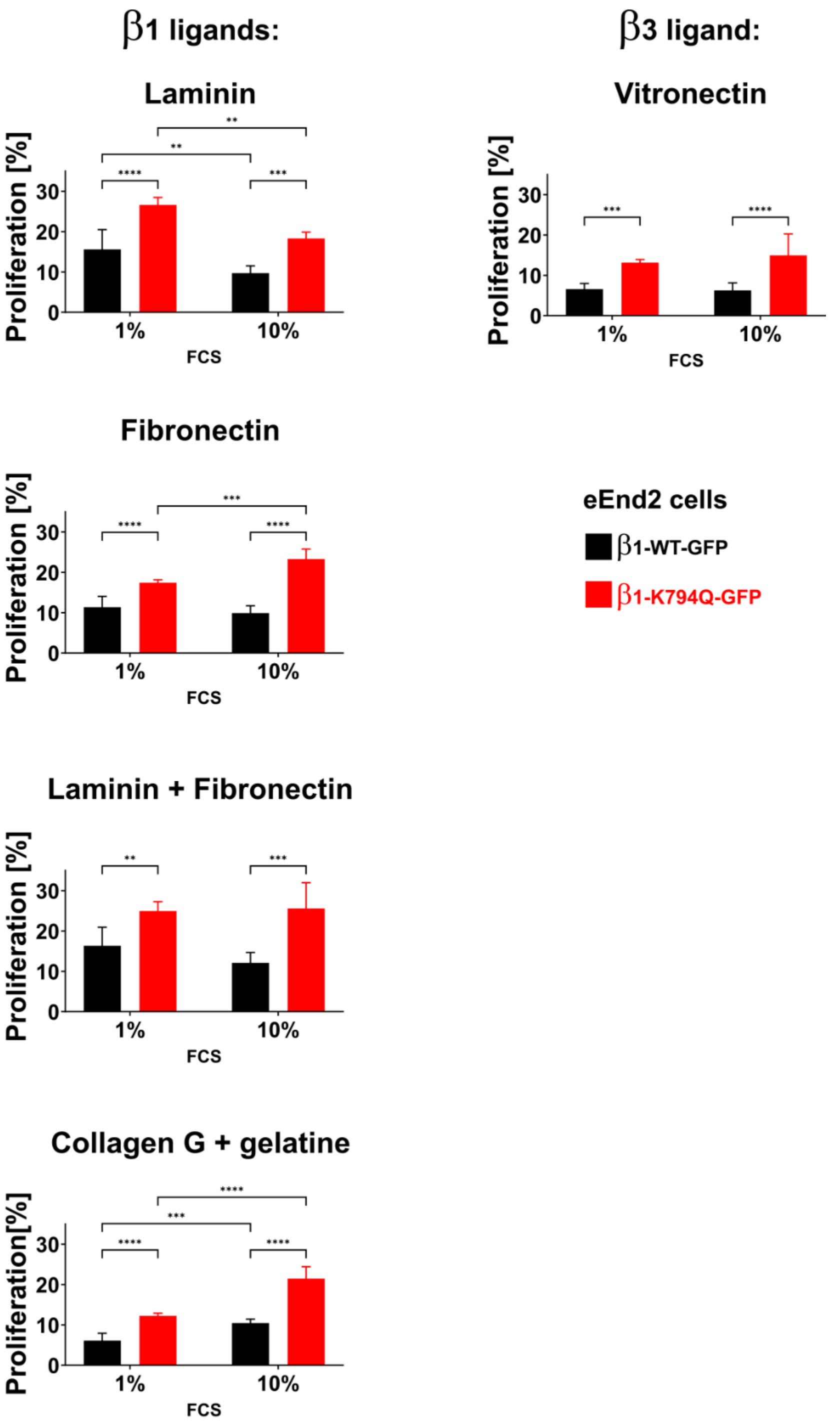

Figure S6: Contribution of Integrin-β1 versus Integrin-β3 extracellular ligands in inducing eEnd2 cell proliferation and modulation by permanent Integrin-β1 acetylation. eEnd2 cells expressing either version of the chimeric β1-GFP (WT or K794Q) were cultured on the indicated ECM substrates in the presence of the indicated amount of serum and EdU-incorporation was evaluated. The difference in proliferation due to Integrin-β1 versus β3 specific extracellular ligands were determined. Integrin-β1 preferred ligands: laminin (α3β1 ligand), fibronectin (for α5β1), collagen and the collagen by-product gelatine (for α1/β1 and α2β1); Integrin-β3 ligand: vitronectin (for αVβ3). Data are presented as mean ± sd. The significance of the difference in proliferation between Integrin-ligand induced and serum-stimulation was indicated with stars when the p-value ≤0.05 in two-away ANOVA test. The data is representative of a single experiment with 3 to 6 replicates per condition. This figure clarifies eventual contributions of Integrin-β1 / β3 crosstalk in the increase of endothelial cell proliferation induced by the permanent acetylation of Integrin-β1. The figure is related to figure 4 of the paper.

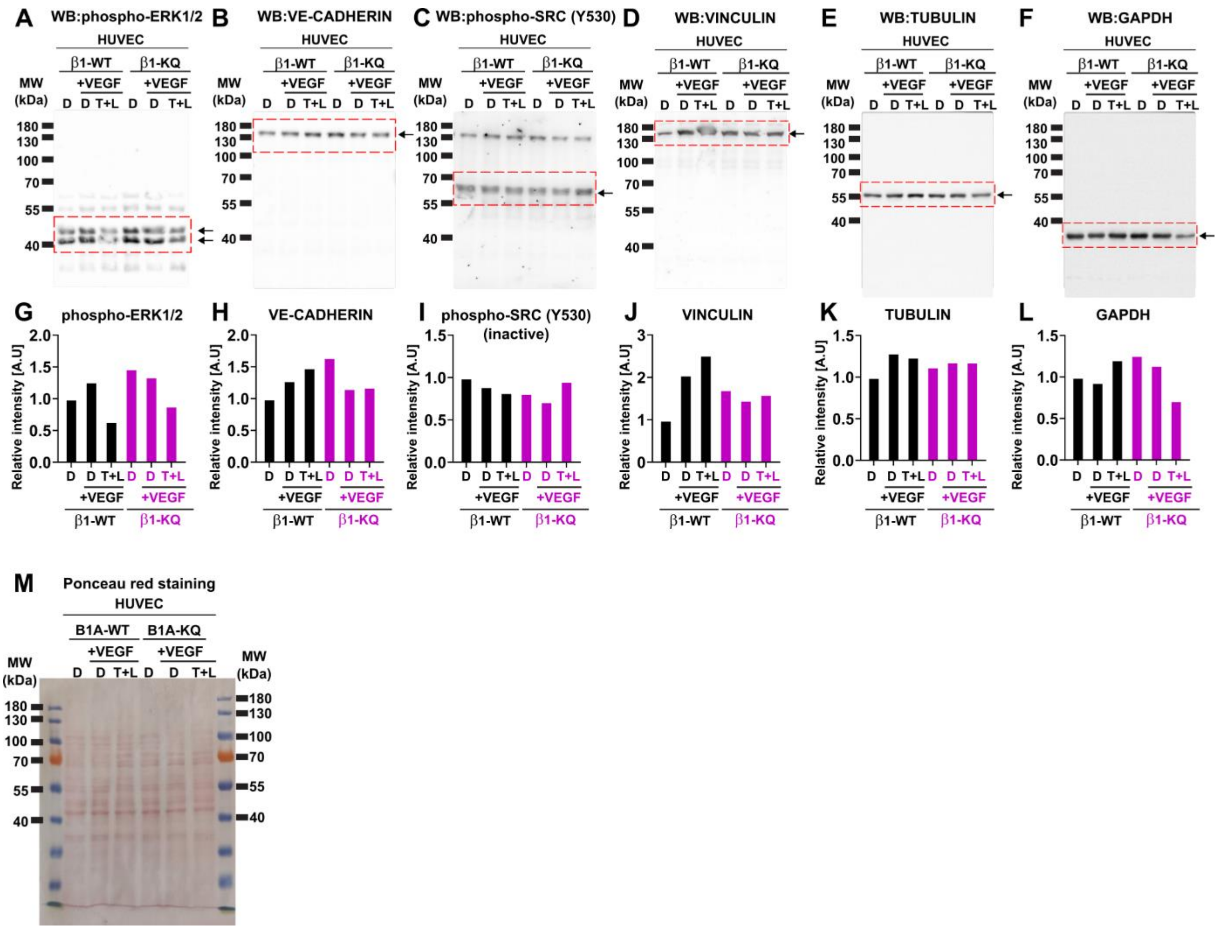

Figure S7: Western blot analysis of signaling pathways in β1-WT or β1-K794Q transfected HUVEC upon VEGF challenge. A, G, MAPK signaling pathways was monitored by staining for phospho-ERK (pERK)1/2 and relative intensity of bands quantified (E). B, H. Anti-VE-CADHERIN staining (B) and quantification in (H) was used to analyze junctional cohesion. C, I. CSK/SRC/FAK pathways (C) was analyzed and quantified in (I) by staining for phospho-Y530-SRC, which indicates inactivated SRC by CSK activity. D, J. VINCULIN protein was analyzed (D) and quantified (J) to detect changes in cell-matrix adhesion. TUBULIN-α (blot in E and quantification in K) and GAPDH (blot in F and quantification in L) content of the samples were stained to check how they evolve with protein loading. The equal amount of protein loading was checked by Ponceau red staining of the nitrocellulose membrane (M). Please note that red dashed rectangles and arrows indicate the relevant bands in the same blot after washing and exposure to a different set of antibodies. Confluent HUVEC constitutively expressing either β1-WT or β1-K794Q, were grown in control medium or stimulated with VEGF in presence or not of the mix of inhibitors (drug T+L: Tyrphostin47 and LY294002; drug D: DMSO used as control of the inhibitor mix). The shown western blots are representative of 3 independent experiments with similar results on the influence of β1-K794Q expression on the constitutive activation of MAPK signaling pathways. This figure supports data in the main figure 4 of the paper.

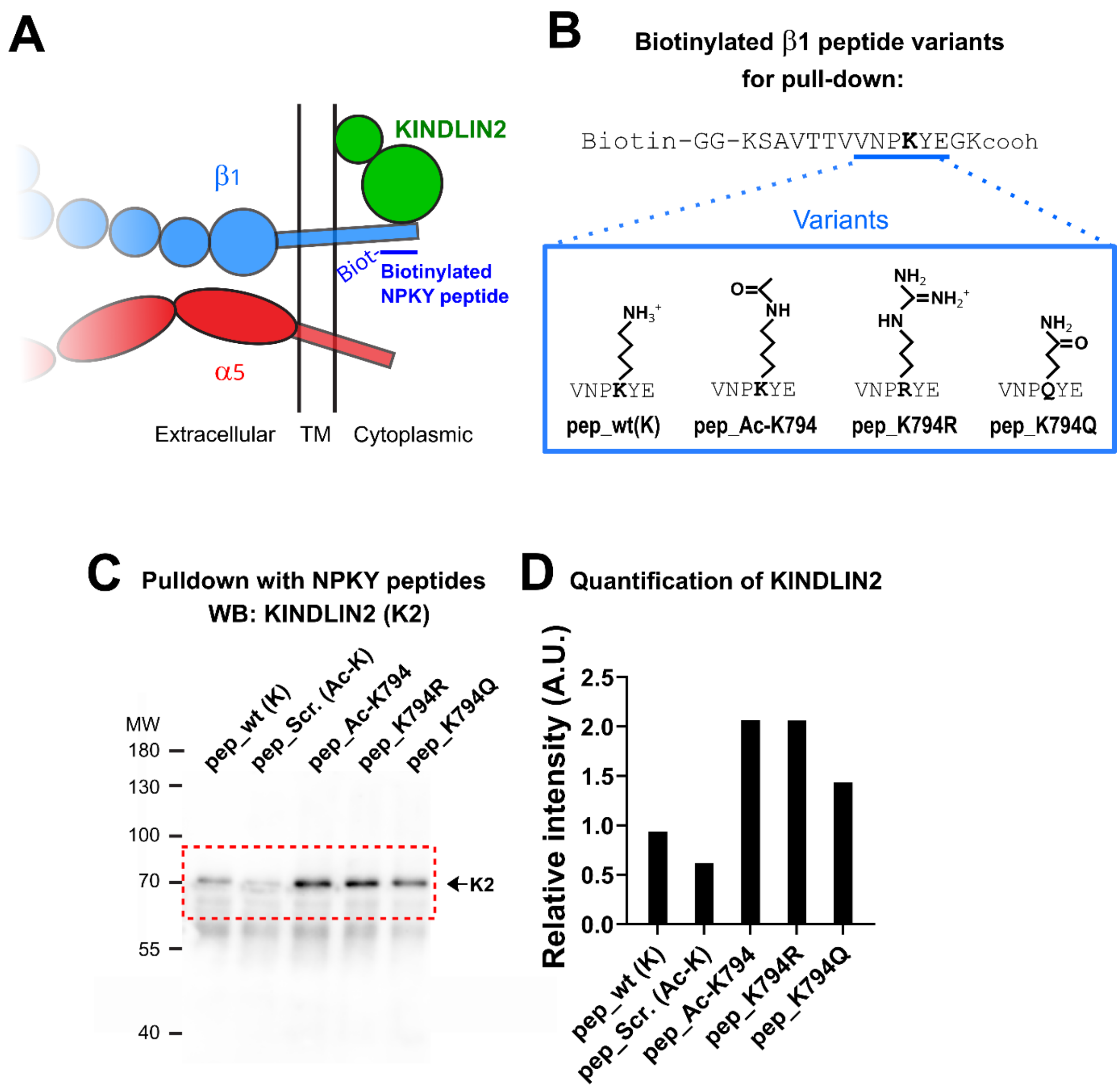

Figure S8: Effect of Integrin-β1 acetylation on KINDLIN2 binding in vitro. A. Illustration of the KINDLIN2 binding site on the distal Integrin-β1 cytoplasmic tail, and the localization of the biotinylated NPKY peptides. The cytoplasmic, transmembrane (TM) and extracellular parts of Integrin-β1 are indicated. B. Sequences of biotinylated peptides encompassing the NPKY-motif (underscored in blue) as well as their variations (in the blue rectangle) used for pulldown analysis. Lys13 of the biotinylated β1-Integrin peptide pep_wt(K) of the sequence Biot-GGKSAVTTVVNP**K**YEGK, corresponding to K794, was either mutated to Gln (K794Q; acetylation mimetic) or Arg (K794R; mimicking the size of the acetylated Lys residue, but with the conservation of the positive charge), and synthesized acetyl-lysine residue. C. Western blot of the pulldown of KINDLIN2 from HUVEC extracts by the biotinylated Integrin-β1 peptides, as illustrated in panel (B), and a scrambled peptide with the acetylated Lys at the same position. The red dashed rectangle and the arrow indicate the band corresponding to endogenous full-length KINDLIN2 (K2). D. Quantification of the KINDLIN2 band from the western blot of the pull-down experiment. The result is representative of >3 independent experiments with HUVEC protein extracts and/or reproduced with other extracts from cells overexpressing a chimeric KINDLIN2-GFP construct. Please note in A that the small green circle touching the plasma membrane of KINDLIN2 represents the inserted PH-domain. This figure support data in the main figure 6.

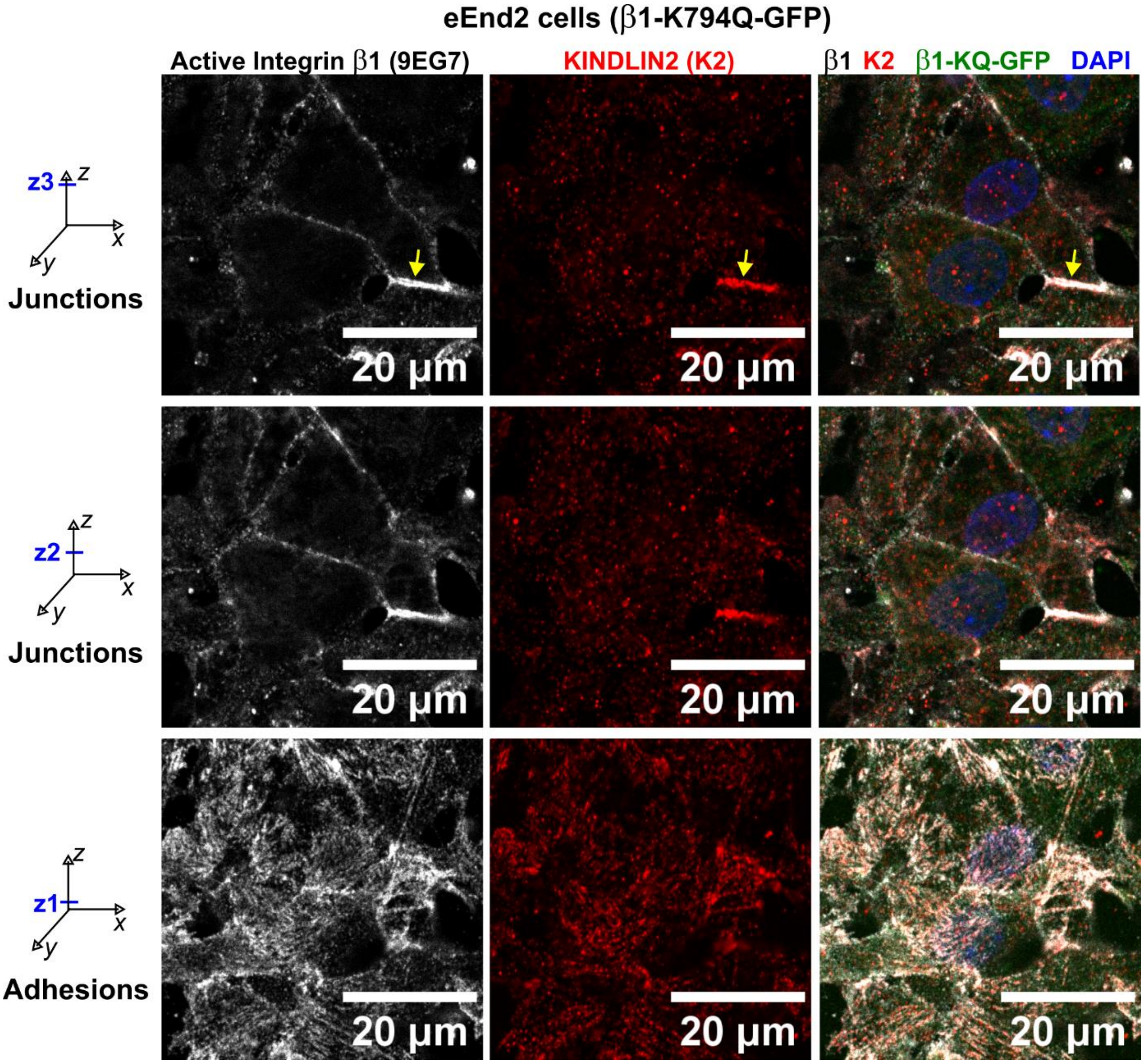

<u>Figure S9</u>: Influence of acetylation mimetic K794Q mutant of Integrin-β1 on the recruitment of KINDLIN2 at junctional and basal cell membranes. Distribution of KINDLIN2 and ligand-bound (active) Integrin-β1 (9EG7 staining) at adhesions and junctions of eEnd2 cells expressing the acetylation mimetic β1-K794Q. Representative multi-channel confocal images at different z-depth are shown to highlight the presence of both KINDLIN2 and the active form of Integrin-β1 at cell-cell junctions and cell-matrix adhesions. On the left, the confocal position (blue line) is indicated on the z-axis. Single channels of Integrin-β1 and KINDLIN2 are presented, as well as their merge including the chimeric β1-GFP and the nuclear DAPI staining. Integrin-β1 is shown in gray, KINDLIN2 in red, GFP in green and DAPI in blue. Scalebar = 20 µm. The yellow arrows highlight strong enrichment of active Integrin-β1 and KINDLIN2 at cell junctions. This demonstrates that in β1-K794Q expressing cells, KINDLIN2 is also captured outside of classical cell-matrix adhesions by Integrin-β1. This may explain the global decrease of the ratio of KINDLIN2 per active Integrin-β1 at cell-matrix adhesions at the basal plasma membrane seen for β1-K794Q cells compared to β1-WT cells. This observation was made in eEnd2 cells, as well as HUVEC. The shown data is from one representative out of 3 independent experiments. This supplementary figure is related to Figure 6.

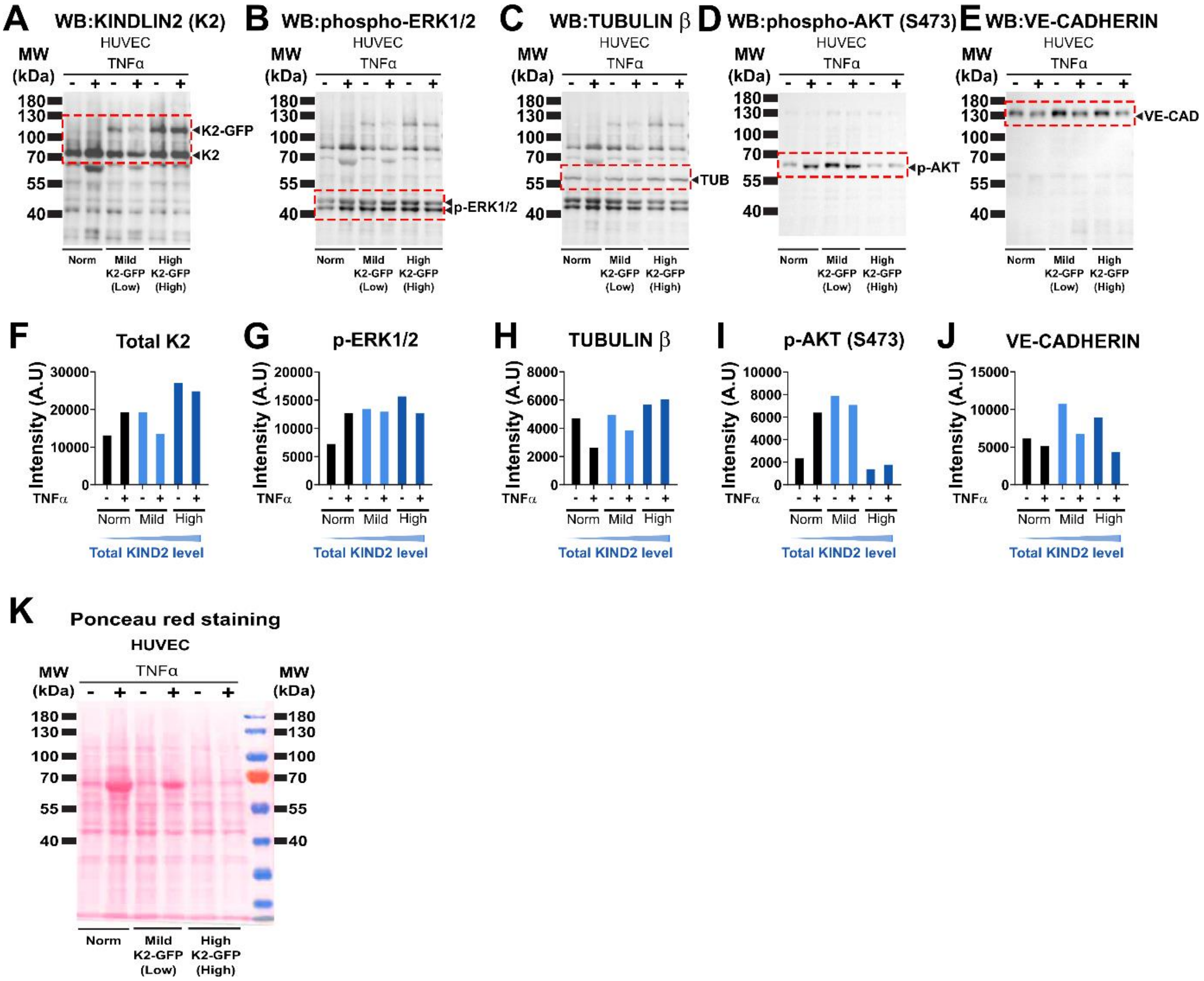

Figure S10: Western blotting to analyse the effects of cellular KINDLIN2 levels on signalling pathways in HUVEC upon TNFα challenge. HUVEC were transduced or not (Norm: endogenous level of KINDLIN2) to express different levels of KINDLIN2-turboGFP (Mild: endogenous KINDLIN2 plus low amounts of the chimeric KINLDIN2-turboGFP, High: endogenous KINDLIN2 plus high amounts of KINDLIN2-turboGFP), under the transcriptional control of the EF1α promoter. Immunoblots: KINDLIN2 (A) shows both the endogenous KINDLIN2 at ~75 KDa and the chimeric KINDLIN-2-GFP (above 100 KDa), their quantification in (F). (B) phospho-ERK1/2 appear at 42 and 44 KDa, and their intensities are quantified in (G), TUBULIN α (C) is localized above 55 KDa and quantified in (H), phospho-AKT (S473) (D) migrates at ~60 KDa and is quantified in (I), VE-CADHERIN (E) appears at ~130 KDa and is quantified in (J). The equal amount of protein loading was checked by Ponceau red staining of the nitrocellulose membrane prior to blotting (K). The blots are representative of one single experiment, repeated once for KINDLIN-2 and VE-CADHERIN. This supplementary figure is related to the main Figure 8.

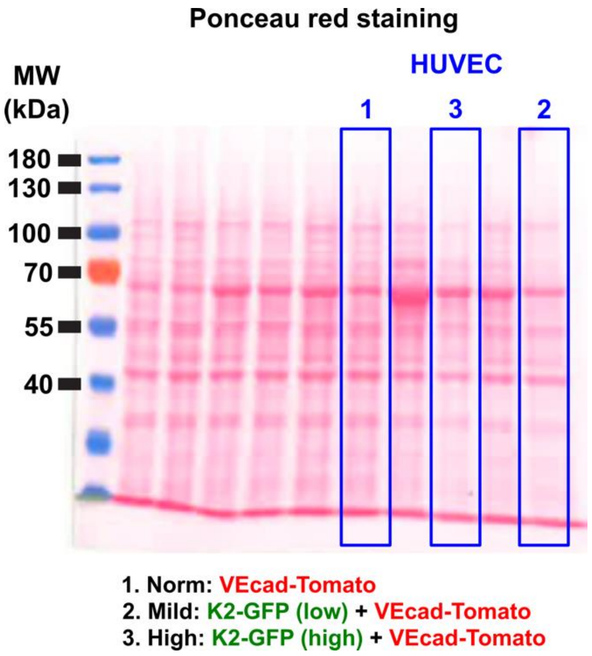

Figure S11: Control of equal amount of proteins transferred onto nitrocellulose. Ponceau staining of the samples analysed by Western blotting shown in the main Figure 8E, the blue rectangles indicate the cropped lanes used to compose the right (anti-VE-CADHERIN) and left (anti-KINDLIN2) panels of the Figure 8E.

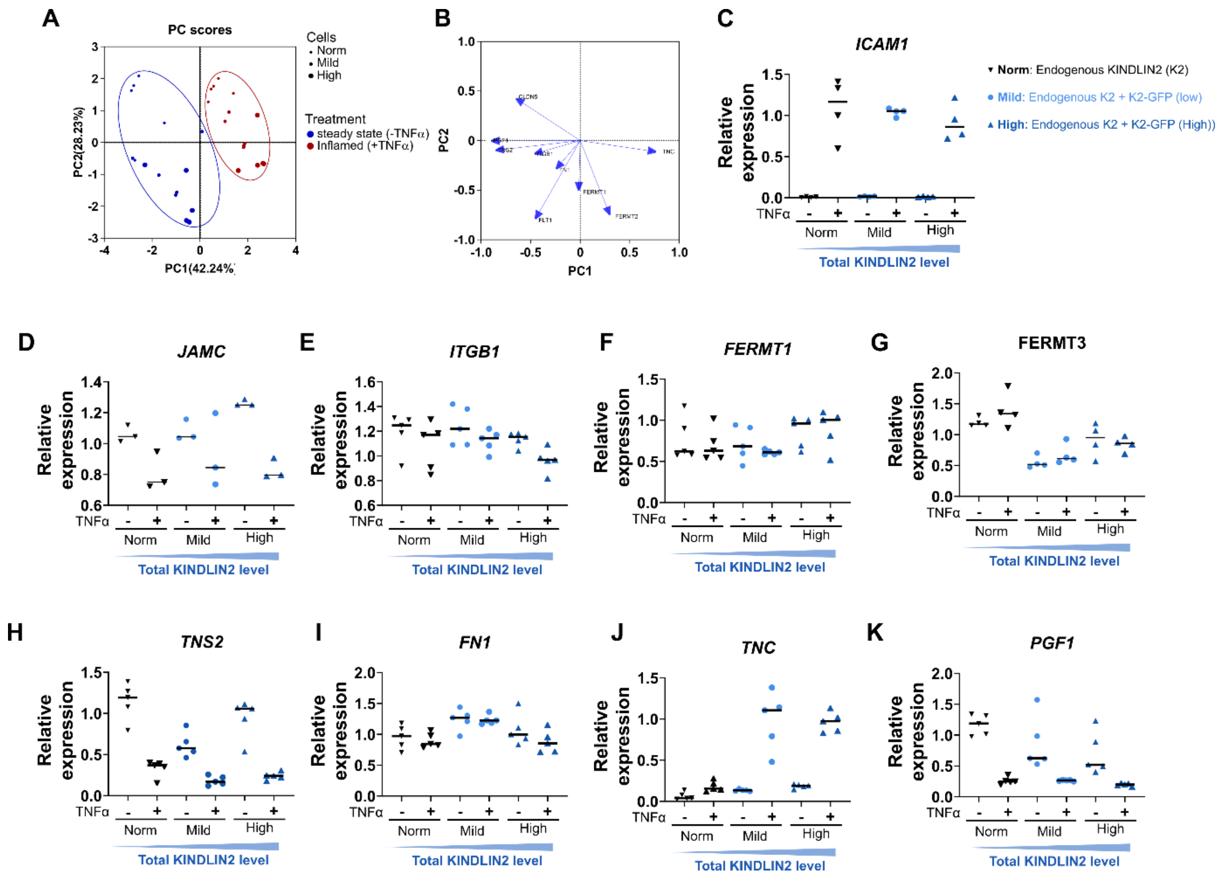

<u>Figure S12</u>: Impact of KINDLIN2 levels on the mRNA expression profiles of several genes involved in maintaining cell-cell junctions, cell-ECM adhesions and angiogenesis upon inflammation (TNFα challenge). A. Principal component analysis (PCA) scores of genes quantified by qPCR of total mRNA from HUVEC expressing different levels of total KINDLIN2 and stimulated or not with TNFα. HUVEC expressing only the endogenous KINDLIN2 (Norm), overexpressing in addition a low amount of KINDLIN2-GFP (Mild) or overexpressing a high amount of KINDLIN2-GFP (High) were used for these experiments. The actual mRNA levels of KINDLIN2 is show in figure 8 in the main manuscript. B. Analysed transcripts of genes and their trends profiles in the PCA is indicating their relationship. The arrows are indications of the trends of transcript expression induced by the combination of KINDLIN2 levels and inflammation (TNFα treatment). C-K. Analysis by qPCR of the expression of a few selected genes, as a function of KINDLIN2 levels in HUVEC upon inflammation. Three of the graphs are included in Figure 8 of the paper. The mRNA level of *ICAM1* (C) was used as a relevant control of an efficient induction of HUVEC inflammation by TNFα treatment. The other transcripts quantified are *JAMC* (D), *ITGB1* (E), *FERMT1* (F), *FERMT3* (G), *TNS2* (H), *FN1* (I), *TNC* (J) and *PGF1* (K). An impact of KINDLIN2 levels was noticeable on several genes including: *JAMC*, *FERMT1*, *FERMT3* and *PGF1*. A clear synergistic effect was induced by TNFα and KINDLIN2 levels for a strong induction of TENASCIN-C transcripts *TNC*. TNFα showed strong decreasing or increasing effects on most of the genes. Each dot spot represents the mean of biological replicates in a qPCR experiment and the lines represent the median. The data were combined from different qPCR experiments (3-5 independent experiments according to the analysed transcripts). This supplementary figure is related to Figure 8.

Table S1. Most enriched gene ontology terms, related to Figure 1.

Top of the enriched BP/CC/MF GO terms:

Thresholds: pvalue < 0.05 & FC ≥ 2

	ID	Description	Ontology	Number of significant genes
1	GO:0071944	cell periphery	CC	174
2	GO:0005886	plasma membrane	CC	168
3	GO:0048731	system development	BP	186
4	GO:0007399	nervous system development	BP	111
5	GO:0032501	multicellular organismal process	BP	236
6	GO:0044459	plasma membrane part	CC	97
7	GO:0005509	calcium ion binding	MF	36
8	GO:0005576	extracellular region	CC	73
9	GO:0005615	extracellular space	CC	53
10	GO:0022008	neurogenesis	BP	88
11	GO:0031012	extracellular matrix	CC	30
12	GO:0044421	extracellular region part	CC	62
13	GO:0048856	anatomical structure development	BP	210
14	GO:0007275	multicellular organism development	BP	197
15	GO:0006928	movement of cell or subcellular component	BP	94
16	GO:0030154	cell differentiation	BP	160
17	GO:0048513	animal organ development	BP	137
18	GO:0032879	regulation of localization	BP	123
19	GO:0009653	anatomical structure morphogenesis	BP	121
20	GO:0032502	developmental process	BP	220

Table S2. Most enriched Kyoto Encyclopedia of Genes and Genomes pathways, related to Figure 1.

Top of the enriched KEGG pathways:

Thresholds: pvalue < 0.05 & FC ≥ 2

	ID	Description	Number of significant genes
1	path:mmu04115	p53 signaling pathway	10
2	path:mmu04640	Hematopoietic cell lineage	7
3	path:mmu04810	Regulation of actin cytoskeleton	16
4	path:mmu04014	Ras signaling pathway	16
5	path:mmu04977	Vitamin digestion and absorption	4
6	path:mmu00565	Ether lipid metabolism	5
7	path:mmu04010	MAPK signaling pathway	19
8	path:mmu00591	Linoleic acid metabolism	3
9	path:mmu05200	Pathways in cancer	29
10	path:mmu04510	Focal adhesion	14
11	path:mmu05218	Melanoma	7
12	path:mmu00592	alpha-Linolenic acid metabolism	3
13	path:mmu05202	Transcriptional misregulation in cancer	12
14	path:mmu00590	Arachidonic acid metabolism	4
15	path:mmu04015	Rap1 signaling pathway	12
16	path:mmu05226	Gastric cancer	9
17	path:mmu04514	Cell adhesion molecules (CAMs)	6
18	path:mmu05414	Dilated cardiomyopathy (DCM)	5
19	path:mmu05410	Hypertrophic cardiomyopathy (HCM)	5
20	path:mmu04151	PI3K-Akt signaling pathway	17

<u>Table S3</u>. List of primers pairs used for qPCR, related to the STAR Methods.

Gene (Human)	**Forward**	**Reverse**	**Size**	**PrimerBank ID**
FERMT1	GGTGAGGTTGCGAGTCAGC	CCAGACGGCTTTAACAAGGAA	111	116686113c2
FERMT2	CAGACACCCCGAAGAACTTTC	GCCCCTCTAATTCAAGTGCCT	110	201861792c2
FERMT3	GGACTACATCGACTCGTCATGG	CTCCACAATCTTCAGGAGCAC	127	215983054c1
ICAM1	GTATGAACTGAGCAATGTGCAAG	GTTCCACCCGTTCTGGAGTC	119	167466197c3
ITGB1	ATGTGTCAGACCTGCCTTGG	GCTGGGGTAATTTGTCCCGA	151	
JAMC	CTGCTGTTCACAAGGACGAC	CGCCAATGTTCAGGTCATAG	111	
TNC	TCCCAGTGTTCGGTGGATCT	TTGATGCGATGTGTGAAGACA	131	
CLDN5	CTCTGCTGGTTCGCCAACAT	CAGCTCGTACTTCTGCGACA	75	195947371c1
FN1	CGGTGGCTGTCAGTCAAAG	AAACCTCGGCTTCCTCCATAA	130	47132556c1
TNS1	GTACGTCACAGAGAGGATCATCG	GCAGGTAGTTGCCTCCATGTT	122	156142195c1
TNS2	CTTGTATGGACTGCGGCTGG	CTGTAGTCAGGCATCGGGG	150	
GAPDH	GCACAAGAGGAAGAGAGAGACC	AGGGGAGATTCAGTGTGGTG	78	
B2M	TGCTCGCGCTACTCTCTCTTT	TCTGCTGGATGACGTGAGTAAAC	82	
EEF1A1	AGCAAAAATGACCCACCAATG	GGCCTGGATGGTTCAGGATA	70	

Supplemental references:

1. Soto-Ribeiro, M., Kastberger, B., Bachmann, M., Azizi, L., Fouad, K., Jacquier, M.C., Boettiger, D., Bouvard, D., Bastmeyer, M., Hytonen, V.P., and Wehrle-Haller, B. (2019). beta1D integrin splice variant stabilizes integrin dynamics and reduces integrin signaling by limiting paxillin recruitment. J Cell Sci *132*. 10.1242/jcs.224493.
2. Wang, Y., Xu, X., Maglic, D., Dill, M.T., Mojumdar, K., Ng, P.K.-S., Jeong, K.J., Tsang, Y.H., Moreno, D., Bhavana, V.H., et al. (2018). Comprehensive Molecular Characterization of the Hippo Signaling Pathway in Cancer. Cell reports *25*, 1304-1317.e1305. 10.1016/j.celrep.2018.10.001.
